# Identification of novel proteins affected by rotenone in mitochondria of dopaminergic cells

**DOI:** 10.1186/1471-2202-8-67

**Published:** 2007-08-16

**Authors:** Jinghua Jin, Jeanne Davis, David Zhu, Daniel T Kashima, Marc Leroueil, Catherine Pan, Kathleen S Montine, Jing Zhang

**Affiliations:** 1 Department of Pathology, University of Washington School of Medicine, Seattle, WA, USA; 2 Department of Neurobiology, Zhejiang University School of Medicine, Hangzhou, Zhejiang, China

## Abstract

**Background:**

Many studies have shown that mitochondrial dysfunction, complex I inhibition in particular, is involved in the pathogenesis of Parkinson's disease (PD). Rotenone, a specific inhibitor of mitochondrial complex I, has been shown to produce neurodegeneration in rats as well as in many cellular models that closely resemble PD. However, the mechanisms through which complex I dysfunction might produce neurotoxicity are as yet unknown. A comprehensive analysis of the mitochondrial protein expression profile affected by rotenone can provide important insight into the role of mitochondrial dysfunction in PD.

**Results:**

Here, we present our findings using a recently developed proteomic technology called SILAC (stable isotope labeling by amino acids in cell culture) combined with polyacrylamide gel electrophoresis and liquid chromatography-tandem mass spectrometry to compare the mitochondrial protein profiles of MES cells (a dopaminergic cell line) exposed to rotenone versus control. We identified 1722 proteins, 950 of which are already designated as mitochondrial proteins based on database search. Among these 950 mitochondrial proteins, 110 displayed significant changes in relative abundance after rotenone treatment. Five of these selected proteins were further validated for their cellular location and/or treatment effect of rotenone. Among them, two were confirmed by confocal microscopy for mitochondrial localization and three were confirmed by Western blotting (WB) for their regulation by rotenone.

**Conclusion:**

Our findings represent the first report of these mitochondrial proteins affected by rotenone; further characterization of these proteins may shed more light on PD pathogenesis.

## Background

Parkinson's disease (PD) is the second most common neurodegenerative disorder after Alzheimer's disease (AD) [1,2]. Pathological characteristics of PD include the progressive and relatively selective loss of nigrostriatal dopaminergic neurons and the deposit of protein aggregates in the remaining neurons called Lewy bodies (LBs) [3]. The mechanisms underlying PD development and LB formation are not fully characterized, although increasing evidence suggests that mitochondrial dysfunction, oxidative damage, excitotoxicity, and inflammation are contributing factors [4,5]. Of these potential mechanisms, mitochondrial dysfunction has been studied most extensively. It has been reported by many groups that there is a partial inhibition (20–40%) of respiratory chain complex I activity in PD patients [6-8]. The importance of complex I inhibition is further substantiated by the fact that mitochondrial toxicants, e.g. 1-methyl-4-phenyl-1,2,3,6-tetrahydropyridine (MPTP) [9], a contaminant of a synthetic heroin, and rotenone [10], a plant-derived pesticide, recapitulate parkinsonism in animals that closely resembles human PD.

Rotenone is a lipophilic compound that freely crosses cell membranes and accesses cytoplasm and mitochondria. Its application in PD research has grown exponentially over the last few years largely due to the discovery of its ability to produce many features of PD in rats, including development of α-synuclein-positive cytoplasmic inclusions similar to LBs in human PD in the remaining nigral neurons [11]. *In vitro*, rotenone appears to produce many cellular effects, e.g. caspase 3 activation (and apoptosis), change in mitochondrial membrane potential, accumulation and aggregation of α-synuclein and ubiquitin, oxidative damage, and endoplasmic reticulum stress [12-16].

Although many of the findings observed in rotenone induced cellular and animal models of PD are also present in the brains of patients with PD, the precise mechanisms through which complex I dysfunction might produce neurotoxicity are as yet unknown. One of the major issues centers on what happens after complex I is inhibited by rotenone. While this question has been approached thus far by testing one hypothesis at a time, the rapidly emerging field of proteomics offers great potential to globally identifying and characterizing mitochondrial proteins involved in these processes in a nonbiased manner. Indeed, in the last few years, we, as well as others, have already started to utilize this technology to extensively characterize the proteins interacting with α-synuclein [17] and DJ-1 [18,19].

Here, we employed a recently introduced proteomics technology called SILAC (stable isotope labeling by amino acids in cell culture) [20] combined with MudPIT (multidimensional protein identification technology) [21], including SDS-PAGE (polyacrylamide gel electrophoresis) and liquid chromatography (LC), and mass spectrometry (MS) for protein identification as well as quantification in dopaminergic (DAergic) MES cells exposed to rotenone vs. controls. MES cells are a DAergic cell line that have been demonstrated to form cytoplasmic LB-like inclusions after rotenone treatment [17]. In this study, however, only mitochondrial proteins, isolated by sucrose-gradient centrifugation, were studied. A total of 1864 proteins were identified with quantitative proteomics analysis; of those, 1722 proteins were identified as mitochondrial protein or mitochondria-associated proteins and 110 of which displayed significant changes in relative abundance after rotenone treatment. A subset of these proteins was further validated by confocal microscopy and WB to confirm their mitochondrial localization as well as regulation by rotenone. As none of these proteins have been associated with rotenone toxicity previously, our findings represent the first report of these novel mitochondrial proteins affected by rotenone.

## Results

### Evaluation of the purity of mitochondrial isolation

**Figure 1:**
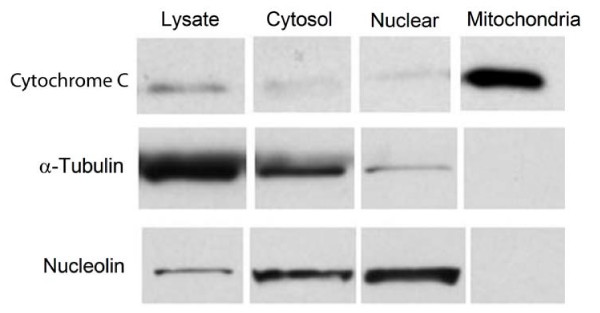
**Purity evaluation of isolated mitochondria by WB**. Equal amounts of protein (20 μg for cytochrome C and nucleolin, 5 μg for tubulin) were loaded onto an 8–16% SDS-PAGE and analyzed by WB with indicated antibodies against marker proteins from mitochondria, cytosol, or nucleus. Antibodies against α-tubulin and nucleolin were used as markers for cytosolic and nuclear fractions, respectively. Antibodies directed against cytochrome C were used as markers for mitochondrial fraction.

To minimize false positive results in identifying mitochondrial proteins, it is critical to isolate mitochondria with minimal contamination of other organelles. To achieve this goal, we first used an optimized purification procedure, allowing for production of high-purity mitochondria from MES cells. This procedure involved three differential centrifugations followed by sucrose density gradient centrifugation using three sucrose densities. The purity of mitochondria was assessed by following the relative distribution of various cellular markers, including cytochrome C for mitochondria, α-tubulin for cytosol, and nucleolin for nucleus. The results, shown in Figure 1, demonstrate that mitochondrial protein cytochrome C was highly enriched in the purified mitochondrial fraction while the cytosolic protein α-tubulin and nuclear protein nucleolin were decreased to near or below the limit of detection in the mitochondrial fraction.

### Identification of mitochondrial proteome in MES cells

Pure mitochondrial preparations were isolated from combined extracts of cells exposed to 20 nM rotenone (in L- [^13^C_6_]Arg) or vehicle (in L- [^12^C_6_]Arg) for 3 days as described previously [22]. SDS-PAGE combined with two-dimensional LC followed by tandem MS (MS/MS) analysis identified a total of 1864 proteins (error rate < 0.05) with more than 2 peptides [see Additional file 1]. These results were a combination of two independent experiments. Among the identified proteins, 228 (12.2%) were previously identified as mitochondrial proteins based on the protein names in the database. One obvious question is how many of the remaining proteins were also mitochondrial proteins? To address this, we searched all the identified proteins against the most comprehensive mitochondrial proteome database, MitoP2, which combines information regarding the genetic, functional and pathogenetic aspects of nuclear-encoded mitochondrial proteins. Besides data for proteins known to be mitochondrial in origin, MitoP2 also provides information about putative mitochondrial proteins identified by homology search tools. Each protein entry is annotated with function, chromosomal localization, subcellular localization, homologs and associated confidence values, GO (Gene Ontology) number, applicable OMIM (Online Mendelian Inheritance in Man) [23], literature references, and cross-references to external databases. Our search results showed that 950 out of 1864 (including 228 proteins that have been previously identified as mitochondrial) proteins were found in MitoP2 [see Additional file 1]. The remaining proteins were further searched against SWISS-Prot database  for subcellular localization or function. 142 proteins were "localized" to cytoplasm, nucleus, ER, golgi, lysosome, microsome, peroxisome or synaptosome and thus considered likely contaminants. The remaining 772 proteins showed unknown functions or unknown localization.

### Proteins affected by rotenone treatment

**Figure 2:**
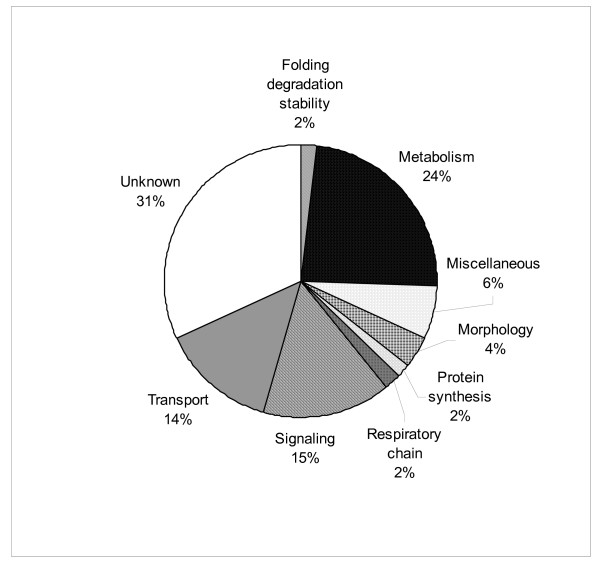
**Functional classification of mitochondrial proteins with relative abundance changes after rotenone treatment**. 110 proteins identified in the mitochondrial database MitoP2 and having 2-fold relative abundance changes after rotenone treatment were classified into the following categories: folding degradation stability, metabolism, morphology, respiratory chain, protein synthesis, signaling, transport, miscellaneous, and unknown functions. For a protein with multiple functions, it is assigned to the one that is best known. While this chart reflects the group as a whole, the distribution was the same regardless of response to rotenone. Functional classification of proteins for each of the four groups shown in Table 1 (ASAPRatio = 0, ≥ 2.0, ≤ 0.5, or = 999) resulted in distributions that were not different from each other (χ^2 ^had P > 0.05 for the four groups).

We, as well as others, have established that rotenone not only induces cell death but also replicates many cellular processes, e.g. mitochondrial dysfunction and oxidative stress [10,24], which occur in human PD. Like our previous studies [17,18,25], MES cells treated with rotenone at 20 nM demonstrated significant cellular toxicity, losing about 50% of cells by day 3 after the treatment started (Trypan blue assay and MTT assay; data not shown). We used 3-day rotenone treatment regimen because our early investigations have indicated that MES cells treated with rotenone for three days demonstrated cytoplasmic LB-like inclusions immunoreactive to anti-α-synuclein [17], a phenomenon not seen in cells exposed for 1 or 2 days. Quantitative analysis of the mitochondrial protein profiles identified 311 proteins that displayed changes in relative abundance in MES cells treated with rotenone vs. controls, using a 2-fold increase or decrease over controls as significant. The results were expressed as combined ASAPRatio (average ratio ± SE) obtained from two independent experiments. By this criterion, a total of 156 and 155 proteins showed a significant increase or decrease, respectively, after rotenone treatment. Of the 311 proteins, 110 could be found in the MitoP2 database. These proteins are listed in Table 1 where those that have been implicated in the cellular processes relevant to neurodegenerative disease or rotenone pathogenesis are noted. Figure 2 shows a pie chart summarizing the functional categories of the 110 proteins. Major classes of proteins included those related to transport, metabolism and signal transduction. Notably, almost a third of proteins listed in Table 1 are without known function.

**Table 1:** Mitochondrial proteins affected by rotenone treatment

**Increased ≥ two-fold after rotenone treatment**			
protein	Description	ASAPRatio*	Ref(s) for relevance to neurodegenerative disease

IPI00319790	Dnaj (Hsp40) homolog, subfamily C, member 7	0	
IPI00331385	Mdj11		
IPI00371053	Similar to dnaj (Hsp40) homolog, subfamily C, member 7		
IPI00128647	Crumbs-like protein 1 precursor	0	
IPI00204778 (IPI00129395)	Large neutral amino acids transporter small, subunit 1	0	
IPI00331577	Solute carrier family 7 (Cationic amino acid transporter y+ system) member 5		
IPI00223004	Peroxisomal CA-dependent solute carrier homolog	0	
IPI00208799	Solute carrier organic anion transporter family member 1A5	0	
IPI00130878	Voltage-dependent T-type calcium channel alpha-1H subunit	0	
IPI00388311	Ensembl_locations(Chr-bp):10-14622136		
IPI00189595	Voltage-dependent T-type calcium channel alpha-1H subunit		
IPI00213584	Alanine – glyoxylate aminotransferase 2 mitochondrial precursor	0	
IPI00365283	Similar to capping protein beta subunit, isoform 2	0	
IPI00406800	Splice isoform 1 of P47757 F-actin capping protein beta subunit		
IPI00113992	GDP-mannose pyrophosphorylase B homolog	0	
IPI00153603	28S ribosomal protein s18c, mitochondrial precursor	0	
IPI00372556	Similar to NG26	0	
IPI00130339	Protein BAT5		
IPI00123138	Leucyl-trna synthetase	0	
IPI00116966	Asparagine synthetase	0	
IPI00406634	Ensembl_locations(Chr-bp):8-124923273	0	[48]
IPI00170128 (IPI00327108)	Paraplegin		
IPI00134390	Microsomal dipeptidase precursor	0	
IPI00200392	SMHS2	0	
IPI00177183	RIKEN cDNA 4833427E09 gene		
IPI00116913	Laminin alpha-5 chain precursor	0	
IPI00331507 (IPI00325517)	Cullin homolog 5	0	
IPI00135708	Dual specificity mitogen-activated protein kinase kinase 2	0	[49]
IPI00407256	Mitogen activated protein kinase kinase 2		
IPI00231331	Dual specificity mitogen-activated protein kinase kinase 2		
IPI00118384	14-3-3 protein epsilon	0	
(IPI00325135)			
IPI00309516	Hedgehog-interacting protein	0	
IPI00318010	Polycystic kidney disease 1-like 2	0	
IPI00368473	Nuclear mitotic apparatus protein 1	0	
IPI00371741	Similar to zinc finger protein TZF-L	0	
IPI00408182	Tmc3 protein	0	
IPI00222063	Weakly similar to hypothetical 71.7 kda protein	0	
IPI00222264	HRIHFB2003 protein homolog	0	
IPI00366079	Similar to expressed sequence AL022641	0.02	
IPI00163011	Thioredoxin domain containing protein 5 precursor		
IPI00396730	Growth differentiation factor 10	0.1 ± 0.02	
IPI00165799	UBX domain-containing protein 2	0.12 ± 0.1	
IPI00371952	Similar to RIKEN cDNA 1300013G12		
IPI00129470	Ran gtpase-activating protein 1	0.13 ± 0.03	
IPI00319167	Hydrocephalus inducing	0.13 ± 0.05	
IPI00310128	Tissue inhibitor of metalloproteinase 2	0.15 ± 0.02	
IPI00132801	Glucagon-like peptide 1 receptor precursor	0.22 ± 0.05	
IPI00311576	Testis expressed gene 10	0.24 ± 0.08	
IPI00400137	RIKEN cDNA 1200011N24	0.27 ± 0.05	
IPI00403336	MKIAA0567 protein		
IPI00117657	Dynamin-like 120 kda protein, mitochondrial precursor		
IPI00341975	RIKEN cDNA 6430598A04 gene	0.27 ± 0.09	
IPI00317309	Annexin A5	0.27 ± 0.12	[50]
IPI00231954	6-phosphofructokinase, type C	0.31 ± 0.06	
IPI00113824	Basement membrane-specific heparan sulfate proteoglycan core protein precursor	0.33 ± 0.09	
IPI00132276 (IPI00210971)	Vesicle-associated membrane protein 3	0.34 ± 0.07	
IPI00210089	Voltage-gated sodium channel	0.35 ± 0.06	[51]
IPI00110560	Talin 1	0.36 ± 0.06	
IPI00362014	similar to talin		
IPI00191107	Similar to mitochondrial ribosomal protein S21	0.37 ± 0.16	
IPI00391281	Ensembl_locations(Chr-bp):2-190609002		
IPI00127069	Sideroflexin 2	0.38 ± 0.05	[52]
IPI00400079	Splice isoform 1 of Q9JLT4 Thioredoxin reductase 2, mitochondrial precursor	0.4 ± 0.19	
IPI00124699	Thioredoxin reductase 2		
IPI00403407	Adult male stomach cDNA, RIKEN full-length enriched library, clone:2210009O12 product:thioredoxin reductase 2, full insert sequence		
IPI00350590	Splice isoform 4 of Q9JLT4 Thioredoxin reductase 2, mitochondrial precursor		
IPI00207072	NADH-ubiquinone oxidoreductase 13 kda-A subunit, mitochondrial precursor	0.41 ± 0.13	
IPI00330862	Villin 2	0.42 ± 0.07	
IPI00111460	Williams-Beuren syndrome chromosome region 16 protein homolog	0.46 ± 0.12	
IPI00189798	Similar to Williams-Beuren syndrome chromosome region 16 homolog		
IPI00112935	Serine hydroxymethyltransferase	0.47 ± 0.06	[53]
IPI00131577	Heme oxygenase 1	0.47 ± 0.12	[54]
IPI00177038	Actin-like protein 2	0.47 ± 0.17	
IPI00400012	Similar to Actin-like protein 2 (Actin-related protein 2)		
IPI00362072	Similar to actin-related protein 2		
IPI00364385	Similar to RIKEN cDNA 5730406I15	0.5 ± 0.19	
IPI00228236	Microsomal signal peptidase 25 kda subunit		

**Decreased ≥ two-fold after rotenone treatment**			

IPI00212082	Ensembl_locations(Chr-bp):1-153887103	2.03 ± 0.62	
IPI00368435	Similar to Ten-m4		
IPI00157497	Ten-m4		
IPI00351867	Odd Oz\ten-m homolog 4		
IPI00210503	Long-chain-fatty-acid – coa ligase 4	2.04 ± 0.44	
IPI00403180 (IPI00378474)	Similar to mitochondrial isoleucine trna synthetase	2.06 ± 0.22	
IPI00408243	Similar to single-stranded DNA binding protein	2.07 ± 0.19	
IPI00124980	Prolactin regulatory element-binding protein	2.09 ± 0.48	
IPI00229040	Retinol dehydrogenase 13	2.15 ± 2.41	
IPI00198369	Sorting nexin 1	2.21 ± 0.6	
IPI00125441	Sorting nexin 1		
IPI00124120	Sacsin	2.26 ± 0.38	
IPI00373012	Similar to sacsin		
IPI00364603	Similar to RNA-binding protein EWS	2.33 ± 0.31	
IPI00191745	Similar to coproporphyrinogen oxidase	2.38 ± 0.41	
IPI00192034 (IPI00114375)	Dihydropyrimidinase related protein-2	2.46 ± 0.44	
IPI00285485	Protein kinase C-binding protein NELL2 precursor	2.57 ± 0.61	
IPI00136067	Jagged 2 precursor	2.73 ± 0.46	
IPI00365920	Jagged 2		
IPI00311509	Aladin	2.9 ± 0.64	
IPI00205182	Interleukin-6 receptor alpha chain precursor	3.03 ± 0.48	
IPI00206288	interleukin 6 receptor		
IPI00344360	Transcription termination factor, mitochondrial	4 ± 1.42	[55]
IPI00370029	Similar to NG28	4.43 ± 0.97	
IPI00378753	Similar to aminopeptidase N	4.68 ± 0.99	
IPI00405386	T-complex protein 10a	5.36 ± 0.87	
IPI00230453	Similar to VIP36-like protein precursor (Lectin, mannose-binding 2-like)	5.45 ± 0.6	
IPI00357887	Similar to lectin, mannose-binding 2-like		
IPI00123314	Sonic hedgehog protein precursor	5.47 ± 0.98	[56]
IPI00196054 (IPI00125256)	C-C chemokine receptor type 2	6.4 ± 1.67	
IPI00353420	Cytoskeletal protein	7.56 ± 1.41	
IPI00388450	Protein tyrosine phosphatase, receptor type, U	8 ± 1.03	
IPI00338565	Mutant fibrillin-1	8.39 ± 2.97	
IPI00122438	Fibrillin 1 precursor		
IPI00229434	Apoptosis stimulating of p53 protein 2	38.26 ± 2.99	
IPI00269076	Adenylate kinase 2	999	
IPI00214038 (IPI00118120)	Myosin Va	999	
IPI00393437	Ensembl_locations(Chr-bp):8-80002855		
IPI00405881	Ensembl_locations(Chr-bp):9-77611564		
IPI00331016	Similar to SEC24 related gene family, member B	999	
IPI00372727	Similar to Protein transport protein Sec24B (SEC24-related protein B)		
IPI00309437	ALDR protein	999	
IPI00387535	Fatty acid coenzyme A ligase, long chain 3	999	
IPI00169772	Long-chain-fatty-acid – coA ligase 3		
IPI00330207	Splicing factor 3B subunit 1	999	[57]
IPI00366952	Splicing factor 3b, subunit 1, 155kd		
IPI00118235	Mitochondrial 60S ribosomal protein L3	999	
IPI00115094	[3-methyl-2-oxobutanoate dehydrogenase [lipoamide]] kinase, mitochondrial precursor	999	
IPI00409229	Ensembl_locations(Chr-bp):7-120047962		
IPI00204344	[3-methyl-2-oxobutanoate dehydrogenase [lipoamide]] kinase, mitochondrial precursor		
IPI00324180	Breast cancer type 2 susceptibility protein homolog	999	
IPI00366614	Breast cancer susceptibility protein BRCA2		
IPI00118021	Gtrgeo22	999	
IPI00313998	Sulfide:quinone oxidoreductase, mitochondrial precursor	999	
IPI00357889	Similar to 28S ribosomal protein S9, mitochondrial precursor (MRP-S9)	999	
IPI00132700	39S ribosomal protein L35, mitochondrial precursor	999	
IPI00189766	Membrane associated progesterone receptor component 1	999	
IPI00208648	Disks large-associated protein 2	999	
IPI00117912	Semaphorin 5B precursor	999	
IPI00400269	Hypothetical protein		
IPI00365296	Similar to sema domain, seven thrombospondin repeats (type 1 and type 1-like), transmembrane domain (TM) and short cytoplasmic domain, (semaphorin) 5B		
IPI00403907	Sema domain, seven thrombospondin repeats (type 1 and type 1-like), transmembrane domain (TM) and short cytoplasmic domain		
IPI00127131	Osa1 nuclear protein	999	
IPI00113214	Ubiquitin carboxyl-terminal hydrolase 5	999	[58]
IPI00118333	RW1 protein	999	
IPI00362105	Similar to proteasome 26S ATPase subunit 6	999	
IPI00125971	26S protease regulatory subunit S10B		
IPI00231757	Proteasome (prosome, macropain) subunit, alpha type 2	999	[59]
IPI00404117	Proteasome subunit alpha type 2		
IPI00318970	Proteasome (Prosome, macropain) subunit, alpha type 2		
IPI00337930	RIKEN cDNA 4930432B04	999	
IPI00203604	Brain protein 44-like protein	999	
IPI00402961	Ensembl_locations(Chr-bp):12-106426680		
IPI00124292	Brain protein 44-like protein		
IPI00406403	Ensembl_locations(Chr-bp):Un_random_NT_060620-5611	999	
IPI00381837	similar to putative pheromone receptor		
IPI00372549	Similar to MHC class I cell surface glycoprotein	999	
IPI00367751	Similar to pecanex 1	999	
IPI00365997	Similar to PHD finger protein 2	999	
IPI00114424	RIKEN cDNA D930036F22 gene	999	
IPI00326141	Augmenter of liver regeneration	999	
IPI00191045	Similar to zinc finger protein 296	999	
IPI00210183	Nuclear receptor binding factor-1	999	

IPI: International protein index. ASAP Ratio: Automated Statistical Analysis of Protein abundance ratio. The results are expressed as ^12^C (DMSO)/^13^C (Rotenone). *: Ratio was expressed as 0 and 999 when only the ^13^C- and ^12^C-labeled peptides were found, respectively, in the sample. Proteins sharing the same peptides but with different protein identification numbers are listed in one cell.

### Validation of a subset of mitochondrial proteins affected by rotenone

While all proteins listed in Table 1 are considered mitochondrial proteins based on the MitoP2 database, not all of them have been validated biochemically or morphologically. Also, as proteomics identification of candidate proteins can be wrong owing to current incorrect or incomplete database, candidate proteins need to be validated with alternative means not only for their identification but also for their quantification. It is obviously impractical to validate all the proteins listed in Table 1. Thus, we chose 5 proteins from Table 1 for their mitochondrial localization as well as quantitative regulation by rotenone; these proteins are: mitogen activated protein kinase kinase 2 (MAP2K2), sacsin, sonic hedgehog (SHH), sorting nexin 1 (SNX1), and vesicle-associated membrane protein 3 (VAMP3). This selection was based on the following rationale: 1) all of these proteins were identified by multiple peptides and also have commercially available antibodies; 2) the selected proteins displayed quantitative changes in response to rotenone and were thus more likely to be biologically interesting and less likely to be nonspecific contaminating proteins; and 3) these proteins are involved in cellular processes that might be important in PD pathogenesis, even though none of them have been linked to PD directly to date.

**Figure 3:**
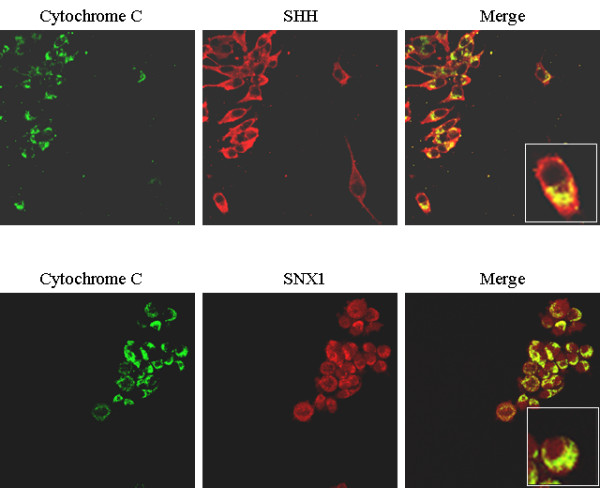
**Co-localization of SHH and SNX1 with cytochrome C in MES cells**. MES cells were fixed and double stained with antibodies against cytochome C (green) and SHH or SNX1 (red). The images were visualized with a confocal microscope. Merged images are shown in yellow when two antibodies are co-localized.

To confirm mitochondrial localization of these proteins, we performed double immunostaining of each of the candidates along with cytochrome C, followed by confocal analysis. Of the antibodies tested, only two (SHH and SNX1), shown in Figure 3, demonstrated enough sensitivity and specificity for immunocytochemistry. SHH was localized to the cytoplasm as well as in the mitochondria but excluded from the nuclei while SNX1 was diffusely localized in the cytoplasm, mitochondria and nuclei. Notably, although the staining patterns of these proteins were different from each other, both of them co-localized with cytochrome C to some extent in the mitochondria. It should be emphasized that it is not surprising to see the presence of these proteins in other cellular compartments, as neither of the candidates is synthesized within the mitochondria.

**Figure 4:**
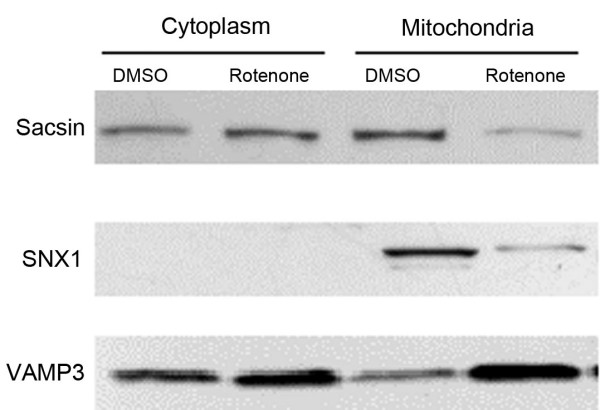
**Validation of rotenone-affected mitochondrial proteins by WB**. Purified mitochondria were isolated from MES cells treated with 20 nM rotenone or vehicle (DMSO), respectively. Equal amounts of mitochondrial proteins (20 μg) were loaded onto an 8–16% or 4–15% SDS-PAGE and analyzed by WB. The WB ratio in mitochondria and cytoplasm was calculated as DMSO/rotenone (D/R) from 3 batches of independent experiments and is shown in Table 2.

To validate quantitative proteomics results, three sets of mitochondrial proteins were isolated from three independent samples. The cytosolic fraction was also included for comparison. The results of these experiments are summarized in Table 2. In the mitochondrial fraction, sacsin and SNX1 levels were significantly decreased by rotenone exposure, while VAMP3 levels were significantly increased. These results were in good agreement with our quantitative proteomics results (Table 2). Sacsin and VAMP3 were also present in the cytosolic fraction of both rotenone and vehicle-treated cells, while SNX1 was not detectable (Figure 4). We did not observe any significant change in the relative abundance of either sacsin or VAMP3 in the cytosolic fraction following rotenone exposure. Antibody against MAP2K2 was not sensitive/specific enough when assessed by WB. In the case of SHH antibody, it responded to immunostaining but showed multiple bands in immunoblotting, which could be biological but made it impossible to compare with the proteomic results. Representative WB results for the other three proteins are shown in Figure 4.

**Table 2:** Summary of validation by WB and immunolocalization

**Protein**	**IPI**	**Function **	**ASAP Ratio**	**WB of mitochondria**	**WB of cytosol**	Co-localized with cyt C*
MAP2K2	00135708 00407256 00231331	Signaling	0	Low sensitivity	Low sensitivity	N/A
Sacsin	00124120 00373012	Folding degradation stability	2.26 ± 0.38	1.77 ± 0.09	0.82 ± 0.02	N/A
SNX1	00198369 00125441	Transport	2.21 ± 0.6	1.51 ± 0.03	Not detected	Yes
SHH	00123314	Signaling	5.47 ± 0.98	Low specificity	Low specificity	Yes
VAMP3	00132276 (00210971)	Transport	0.34 ± 0.07	0.48 ± 0.13	1.16 ± 0.15	N/A

*: Among the five candidate proteins, only antibodies against SHH and SNX1 demonstrated enough sensitivity and specificity for immunocytochemistry.

## Discussion

In this study, we combined SILAC labeling, MudPIT separation of protein/peptide, including SDS-PAGE, and tandem MS to characterize the mitochondrial proteome of MES cells as well as the alterations in the mitochondrial proteome in the presence of rotenone. Several major findings have been found from this investigation, including: 1) a total of 1722 proteins were identified as mitochondrial protein or mitochondria-associated proteins, which is the most comprehensive study of the mitochondrial proteome so far; 2) quantitative analysis of the mitochondrial protein profiles identified 311 proteins that displayed changes in relative abundance in MES cells treated with rotenone vs. controls. Among these, 110 proteins have been represented in the MitoP2 database as mitochondrial proteins; and 3) a subset of novel mitochondria-associated proteins affected by rotenone was validated by confocal microscopy and WB to assess their localization in the mitochondria as well as quantitative changes as determined by quantitative proteomics.

The first achievement of this study is the comprehensive survey of mitochondrial proteins in MES cells, a DAergic cell line widely used for the investigation of PD pathogenesis [26,27]. Not only does extensive characterization of the mitochondrial proteome expand current knowledge regarding the profiles of mitochondrial proteins, it also supplies the necessary information to appropriately interpret the mechanisms of rotenone-induced mitochondrial dysfunction in PD. Comparing the results reported in the literature [25,28-30], the current analysis is a much deeper analysis into the mitochondrial proteome in a neuronal cell line, largely owing to better sample separation by SDS-PAGE and LC, i.e. extensive fractionation prior to MS analysis. This study, using only 80–100 μg of mitochondrial proteins as the starting material, followed by simple LC-MS/MS analysis of 10 gel slices in two independent experiments, afforded the conclusive identification of 1864 proteins. Thus, this simple technology seems to be the most effective method of identifying a large number of proteins from different cell types. Furthermore, this is the first large-scale study where the mitochondrial proteome from the MES cell line was carefully characterized, providing organelle-specific proteomics information for these cells relevant to PD pathogenesis. Among those proteins identified, approximately 50% did not have an apparent orthologue in the MitoP2 database. Several reasons are likely responsible for this absence in the database. First, the current database is still far from complete and most of the missing proteins are hypothetical proteins or proteins with unknown functions. Second, although the purification protocols for mitochondria have been refined by several groups over many years, the exquisite sensitivity of modern mass spectrometers has revealed that it is very difficult, if not impossible, to purify these or any other organelles to homogeneity. In our study, a small portion of those proteins (7.5%) was considered as contaminated from cytoplasm, nucleus, ER, golgi or lysosome, based on the functional interpretation of SWISS-Prot database. While the sucrose gradient ultracentrifugation technique we used to isolate the mitochondrial fraction is widely recognized as the technique producing least contamination [30,31], it remains likely that at least some of these proteins, particularly ER proteins, were co-localized in the mitochondrial fraction as contaminants. It is also possible that some of the "contaminating" proteins are partially localized in mitochondria or interact with other mitochondrial proteins. More detailed studies such as immuno-colocalization experiments would be required to distinguish these different possibilities.

Next, to better understand the mechanisms underlying mitochondrial dysfunction induced by rotenone, we used SILAC to compare the protein profiling of mitochondria in rotenone-treated MES cells versus controls. With SILAC methods, a total of 156 and 155 proteins showed significant increase or decrease after rotenone treatment. Among these, 53 and 57 proteins have been present in the MitoP2 database as mitochondrial proteins respectively. One of the major caveats of proteomics identification of proteins relates to the fact that proteins are inferred by the peptide sequence based on databases, which are currently incomplete. Thus, any given identification, with or without quantitative changes, should be considered provisional until it can be validated via alternative means. It is obviously impractical to validate the entire list of proteins listed in Additional file 1 or even Table 1; to this end, we validated five candidate proteins (sacsin, SNX1, VAMP3, MAP2K2 and SHH) in terms of their cellular location as well as quantitative alternation after rotenone treatment. Two of these proteins, SHH and SNX1, were co-localized to mitochondria convincingly by confocal investigation. Three of these proteins, SNX1, sacsin and VAMP3, when assessed by WB analysis, showed good agreement with our SILAC results. It should be noted that there are many caveats associated with WB and immnocytochemistry, such that a negative result by these assays does not necessarily negate what was observed by quantitative proteomics.

The biological importance of the three validated proteins in PD or rotenone pathogenesis is largely unknown. A potential role of sacsin has been implicated in an early onset neurodegenerative disorder, autosomal recessive spastic ataxia of Charlevoix-Saguenay (ARSACS), which occurs with a high prevalence in the Charlevoix-Saguenay-Lac-Saint-Jean (CSLSJ) region of Quebec [32]. Sacsin contains three regions with Hsp90 subdomains that have similarity to each other and the protein product of an *Arabidopsis thaliana *ORF. The presence of heat shock domains in the sacsin protein suggests its possible function in chaperone-mediated protein folding [32,33]. SNX1 is a protein that binds to the cytoplasmic domain of plasma membrane receptors [34]. It has been proposed to be associated with early endosomes and the regulation of endocytic trafficking of plasma membrane proteins in early endosomes such as epidermal growth factor receptor (EGFR) [35] and protease-activated receptor-1 (PAR1) [36]. VAMP3, an early endosomal vesicular soluble N-ethylmaleimide-sensitive protein attachment protein receptor (v-SNARE), is a membrane trafficking protein of a constitutively recycling pathway [37]. Although neither SNX1 nor VAMP3 has been linked to PD pathogenesis thus far, it has been clearly demonstrated by others that dysfunctions of intracellular trafficking and lysosomal degradation are involved in PD pathogenesis [38,39]. Finally, it needs to be pointed out that this investigation is limited in that we have only compared the protein profiles in MES cells treated with rotenone for three days, when LB like inclusions are found [17]. It would be important in future experiments to examine earlier time points, revealing factors/pathways leading to cell death prior to formation of LB like inclusions.

## Conclusion

In summary, we have employed SILAC, combined with 1D SDS-PAGE, to characterize numerous mitochondrial proteins in MES cells, representing the most extensive profiling study of mitochondrial proteins to date. Furthermore, we identified 110 mitochondrial proteins affected by rotenone. Finally, we have confirmed 2 of these proteins localizing in the mitochondria and quantitatively validated 3 of these proteins by WB. Detailed characterization of these rotenone-responsive novel proteins will likely facilitate a clearer understanding of PD pathogenesis.

## Methods

### Reagents and antibodies

All reagents were purchased from Sigma (St. Louis, MO) unless otherwise specified. Antibodies used included: mitochondrial marker anti-cytochrome C (BD Pharmigen, San Diego, CA); cytosolic marker anti-α-tubulin (Abcam, Cambridge, MA); nuclear marker anti-nucleolin (Novus, Littleton, CO); novel mitochondrial proteins anti-sacsin (BD Pharmingen); anti-sorting nexin protein 1 (SNX1, Proteintech, Chicago, IL); anti-vesicles associated membrane protein 3 (VAMP3, Novus); anti-sonic hedgehog protein (SHH, GeneTex, San Antonio, TX); and anti-mitogen activated protein kinase kinase 2 (MAP2K2, Abgent, San Diego, CA).

### Cell culture and treatment

A DAergic neuronal cell line, MES (a gift from Dr. Le at Baylor College of Medicine in Houston), was used in this study, which expresses most features of human DAergic neurons and has been widely used in PD-related experiments [26,40,41]. Detailed methods for culturing MES cells have been previously described by us [26]. In order to identify the potential mediators of mitochondrial dysfunction induced by rotenone, a newly developed quantitative proteomics technique termed SILAC was used [42]. Briefly, parallel MES cultures were grown in identical culture media except for one essential amino acid, L-arginine: the first media contained the "light" (L- [^12^C_6_]Arg) (Cambridge Isotope Laboratories, Andover, MA) isotope and the other contained the "heavy" (L- [^13^C_6_]Arg) isotope. After being cultured for at least five generations (to achieve near 100% incorporation of arginine [42]), L- [^13^C_6_]Arg- and L- [^12^C_6_]Arg-labeled MES cells were treated with 20 nM rotenone in DMSO or DMSO alone, respectively, for another 3 days.

### Mitochondrial Isolation and WB

Mitochondria were isolated as described previously with minor modifications [28]. After treatment, equal amounts of L- [^13^C_6_]Arg- and L- [^12^C_6_]Arg-labeled MES cells were combined and collected by centrifugation at 260 × *g *for 10 min at 4°C. The cell pellets were washed twice with ice-cold PBS (pH 7.4) and resuspended with 10 volumes of isolation buffer (20 mM HEPES-KOH, pH 7.5, 10 mM KCl, 1.5 mM MgCl2, 1 mM EDTA, 1 mM EGTA, 1 mM DTT, 0.25 M sucrose, and a mixture of protease inhibitors). After 10-min incubation on ice, the cells were homogenized in a glass Dounce homogenizer (Wheaton, Milville, NJ). The homogenates were centrifuged twice at 650 × *g *for 10 min at 4°C to remove nuclei and unbroken cells. The postnuclear supernatants were centrifuged at 12,500 × *g *for 25 min at 4°C, and the pellets were resuspended carefully in mitochondrial isolation buffer and centrifuged again at 12,500 × *g *for 25 min. The heavy membrane fraction was then resuspended in isotonic sucrose buffer (0.25 M sucrose, 1 mM EDTA, and 10 mM Tris-HCl, pH 7.4), layered on a 1.0/1.5 M discontinuous sucrose gradient, and centrifuged at 60,000 × *g *for 20 min at 4°C. The mitochondria collected from the phase between the 1.0 and 1.5 M sucrose gradient were diluted in isolation buffer, and centrifuged again at 15,000 × *g *for 20 min to pellet the mitochondria. Purified mitochondrial pellets were washed with isolation buffer, solubilized in RIPA buffer (50 mM Tris-HCl, pH 8.0, 150 mM NaCl, 0.1% NP-40, 0.05% SDS, 0.5% deoxycholate sodium, and a mixture of protease inhibitors) and centrifuged at 14,000 × *g *for 10 min. The supernatant was collected, and protein concentration was determined by a Micro-BCA protein concentration determination kit (Pierce, Rockford, IL). For WB, equal amounts of protein of various subcellular fractions were loaded in each lane of an 8–16% Tris/glycine/SDS gel (Bio-rad, Hercules, CA). After gel electrophoresis and protein transfer, the membranes were probed with various primary and corresponding secondary antibodies against marker proteins from different cellular compartments. Immunoreactivity was detected with an ECL method (PerkinElmer, Boston, MA).

### SDS-PAGE and in-gel digestion with trypsin

A total of 100 μg mitochondrial protein was loaded on an 8–16% Tris/glycine/SDS gel (Bio-rad) and run at 100 V for 10 min, then 160 V for 1 h. The gel was stained with Coomassie blue R-250 (50% methanol, 10% acetic acid, 0.1% R-250) for 1 h and destained overnight in a solution containing 5% methanol/7% acetic acid. After imaging, stained protein bands were cut into 10 fractions according to molecular weight and distribution of protein abundance, and each fraction was then excised into smaller pieces of approximately 1 to 2 mm^3^. The gel pieces were destained with 50% methanol/5% acetic acid overnight, and in-gel digestion was performed as described previously [43]. The extracted peptides were desalted with a reverse-phase (RP) Atlantis dC18 column (Waters, Milford, MA).

### Protein identification by LC-MS/MS

Desalted peptides from each fraction were further separated by a two-dimensional microcapillary high performance LC system, which integrates a strong cation-exchange (SCX) column (100 mm in length × 0.32 mm for inner diameter; particle size: 5 μm) with two alternating RP C18 columns (100 mm in length × 0.18 mm for inner diameter), followed by analysis of each peptide with MS/MS in a LCQ DECA PLUS ^XP ^ion trap (ThermoElectron, San Jose, CA). Settings for the LCMS/MS were the following: six fractions were eluted from SCX using a binary gradient of 2–90% solvent D (1.0 M ammonium chloride and 0.1% formic acid in 5% acetonitrile) versus solvent C (0.1% formic acid in 5% acetonitrile). Each fraction was injected onto a RP column automatically with the peptides being resolved using a 300 min binary gradient of 5–80% solvent B (acetonitrile and 0.1% formic acid) versus solvent A (0.1% formic acid in water). A flow rate of 160 μl/min with a split ratio of 1/80 was used. Peptides were eluted directly into the electrospray ionization (ESI) ion trap mass spectrometer capable of data-dependent acquisition. Each full MS scan was followed by two MS/MS scans of the two most intense peaks in the full MS spectrum with dynamic exclusion enabled to allow detection of less-abundant peptide ions. Mass spectrometric scan events and HPLC solvent gradients were controlled by the Xcalibur software (Thermo Finnigan).

### MS/MS data analysis

Proteins from the mitochondrial fraction were later identified automatically using the computer program Sequest™, which searched the MS/MS spectra against the rat + mouse International Protein Index (IPI, v3.01, 43175 entries) database [17,18,22,25,29]. Search parameters for the SILAC-labeled samples used in this study were the following: +6 Da for ^13^C isotopic-labeled arginine, +16 Da for oxidized methionine, +57 Da for carbamidomethyl; mass tolerance ± 3Da. Potential peptides and proteins were further analyzed with PeptideProphet™ and ProteinProphet™ based on statistical models [17,44,45]. PeptideProphet uses various SEQUEST scores and a number of other parameters to calculate a probability score for each identified peptide. The peptides were then assigned a protein identification using the ProteinProphet software. ProteinProphet allows filtering of large-scale data sets with assessment of predictable sensitivity and false-positive identification error rates. In our study, only proteins with a high probability of accuracy (< 5% error rate) were selected. Quantification of the ratio of each protein (isotopically light [control] vs heavy [roteonone treatment]) was calculated using the automated statistical analysis of protein abundance (ASAP) Ratio program [46] and expressed as average ratio ± SE. All of these methods are used routinely in our lab [17,18,22,25,29].

### Bioinformatics Analysis

For prediction of the subcelluar location of proteins, all the proteins identified by MS/MS were searched against the Mouse Mitochondrial Proteome Database [47], which includes summarized results from computational predictions of signaling sequences, proteome mapping, mutant screening, expression profiling, protein-protein interactions and cellular sub-localization studies.

### Double immunofluorescent staining and confocal analysis of candidate proteins

MES cells were seeded on chambered glass slides (Nalge Nunc, Naperville, IL), fixed in 4% paraformaldehyde followed by overnight incubation with primary antibodies to cytochrome C and one of the candidate proteins (SNX1 1:200, VAMP3 1:200, MAP2K2 1:200, SHH 1:200), followed by incubation with secondary antibody (1:200 Flex Fluor^® ^488 goat anti-mouse IgG and 1:200 Flex Fluor^® ^568 goat anti-rabbit IgG or 1:200 Flex Fluor^® ^568 donkey anti-goat IgG, Molecular Probes, Eugene, OR). A laser scanning confocal microscope (Bio-Rad LS2000, Hercules, CA) was used to capture images.

### WB for validation of candidate mitochondrial proteins affected by rotenone

MES cells were treated with 20 nM rotenone or DMSO for 3 days, and mitochondria were isolated as for SILAC experiments described above. The relative intensity of the corresponding bands was quantified with Quantity One (Bio-Rad) and relative changes expressed as the ratio of DMSO-treated to rotenone-treated intensities. At least three independent experiments were performed for each candidate protein.

## Abbreviations

LB, Lewy body; LC, Liquid chromatography; MAP2K2, Mitogen activated protein kinase kinase 2; MudPIT, Multidimensional protein identification technology; PD, Parkinson's disease; SHH, Sonic hedgehog protein; SILAC, Stable isotope labeling by amino acids in cell culture; SNX1, Sorting nexin 1; VAMP3, Vesicle-associated membrane protein 3; WB, Western blotting.

## Authors' contributions

JJ and JZ designed the research. JJ and ML carried out the mitochondrial isolation and proteomic identification. JJ and CP carried out the mass spectrometry analysis. JJ, DZ and DK performed proteomics data analysis and bioinformatics analysis. JJ and JD participated in the candidate proteins validation. JJ, KM and JZ participated in coordination and drafted the manuscript. All authors read and approved the final manuscript.

## Supplementary Material

Additional file 1 Total proteins/groups identified with ≥ 2 peptides in mitochondria isolated from MES cells. The table shows total proteins/groups identified with ≥ 2 peptides in mitochondria isolated from MES cells. Click here for file
