# Supplementary material for: Identification of novel proteins affected by rotenone in mitochondria of dopaminergic cells
Source: BMC Neurosci. 2007 Aug 16;8:67. doi: 10.1186/1471-2202-8-67 (PMC2000881; doi:10.1186/1471-2202-8-67)
Supplement: Additional file 1 — Total proteins/groups identified with ≥ 2 peptides in mitochondria isolated from MES cells. The table shows total proteins/groups identified with ≥ 2 peptides in mitochondria isolated from MES cells. [file 1471-2202-8-67-S1.doc]

**Appendix I: Total proteins/groups identified with > 2 peptides in mitochondria isolated from MES cells**

(IPI: InteRNAtional protein index. P: probability (error rate <0.05). Proteins are listed by alphabetical order. The same proteins with different IPI entries are grouped in the same cell. Proteins sharing the same peptides but with different protein identification numbers are listed in one cell and set-off by lower-case letters.)

| P | protein | % coverage | # of unique peps | Description | Localization |
| --- | --- | --- | --- | --- | --- |
| 1 | TRYP_PIG | 30.3 | 10 |  |  |
| 1 | IPI00115094  IPI00409229  (IPI00204344) | 8.3 | 3 | [3-methyl-2-oxobutanoate dehydrogenase [lipoamide]] kinase mitochondrial precursor Ensembl_locations(Chr-bp):7-120047962 | mt |
| 1 | IPI00316329 | 7.4 | 3 | 0 day neonate head cDNA RIKEN full-length enriched library clone:4833436C19 full insert sequence | mt |
| 1 | IPI00132531 | 25.9 | 5 | 0610007D05Rik protein | unknown |
| 1 | IPI00121576 | 26.9 | 7 | 0610008C08Rik protein | unknown |
| 1 | IPI00316622 IPI00198620 | 13.7 | 4 | 0610008F14Rik protein ATP synthase delta chain mitochondrial precursor | mt |
| 1 | IPI00121322 | 14.1 | 4 | 0610010I20Rik protein | unknown |
| 1 | IPI00131843 | 18.5 | 4 | 0610011N22Rik protein | mt |
| 1 | IPI00121079 | 80.1 | 18 | 0610016L08Rik protein | unknown |
| 1 | IPI00132413 | 29.2 | 9 | 0610027A18Rik protein | unknown |
| 1 | IPI00120826 IPI00196589 | 7.6 | 2 | 0610038P07Rik protein Translocon-associated protein gamma subunit | synaptsome |
| 1 | IPI00132412 | 48.5 | 17 | 0610040E02Rik protein | unknown |
| 1 | IPI00133562 | 31.3 | 17 | 0610041L09Rik protein | unknown |
| 1 | IPI00120671 | 38.7 | 7 | 0710001C05Rik protein | unknown |
| 1 | IPI00108014 IPI00408180 IPI00313390 | 17.8 | 6 | 0710001P09Rik protein 1700021B03Rik protein Similar to RIKEN cDNA 0710001P09 gene | mt |
| 1 | IPI00338536 IPI00387539 | 31.6 | 15 | 0710008N11Rik protein 0710008N11Rik protein | unknown |
| 1 | IPI00263863 (IPI00326433) | 46.6 | 16 | 10 kda heat shock protein mitochondrial | mt |
| 1 | IPI00120232 | 38.4 | 5 | 1010001M04Rik protein | unknown |
| 0.74 | IPI00330333 IPI00363654 | 2.8 | 2 | 1100001D10Rik protein Similar to 1100001D10Rik protein | unknown |
| 1 | IPI00134103 | 20.4 | 3 | 1110001A23Rik protein | unknown |
| 1 | IPI00132330 | 13.6 | 2 | 1110001O19Rik protein | ER |
| 1 | IPI00132765 IPI00201454 | 18.9 | 3 | 1110002E23Rik protein Ensembl_locations(Chr-bp):9-8770740 | unknown |
| 0.98 | IPI00133215 | 21.2 | 2 | 1110002H15Rik protein | nuleus |
| 0.91 | IPI00133920 IPI00364566 | 10.6 | 2 | 1110003H02Rik protein Similar to SEC13 related gene | unknown |
| 1 | IPI00133583 IPI00203640 | 18.1 | 5 | 1110003P16Rik protein Similar to DNAj (Hsp40) homolog subfamily D member 1 | mt |
| 0.97 | IPI00380484 | 6.3 | 2 | 1110007C24Rik protein | unknown |
| 1 | IPI00133750 | 34.7 | 5 | 1110007K17Rik protein | unknown |
| 1 | IPI00133706 | 55.2 | 8 | 1110011F09Rik protein | unknown |
| 1 | IPI00121359 | 39.8 | 12 | 1110017G11Rik protein | unknown |
| 1 | IPI00133002 | 61 | 3 | 1110057H19Rik protein | unknown |
| 1 | IPI00132623 | 34.6 | 3 | 1190008J14Rik protein | golgi |
| 1 | IPI00120165 | 18.8 | 6 | 1200003H03Rik protein | unknown |
| 1 | IPI00387379 (IPI00132085) | 43.6 | 15 | 1200012F07Rik protein | unknown |
| 1 | IPI00120083 | 19.8 | 4 | 1200015A22Rik protein | unknown |
| 1 | IPI00132490 | 24.6 | 9 | 1200015P13Rik protein | unknown |
| 1 | IPI00229008 | 10.9 | 2 | 13 days embryo liver cDNA RIKEN full-length enriched library clone:2510049O12 product:NADH dehydrogenase (Ubiquinone) Fe-S protein 4 full insert sequence | membrane |
| 1 | IPI00119575 IPI00231770 IPI00225331 | 10.8 | 2 | 1300018C22Rik protein Protein kinase, camp dependent regulatory, type I, alpha  Protein kinase | mt |
| 1 | IPI00119559 IPI00322286 | 5.6 | 2 | 1300018J16Rik protein RIKEN cDNA 1300018J16 gene | mt |
| 1 | IPI00110386 | 12.4 | 5 | 14 17 days embryo head cDNA RIKEN full-length enriched library clone:3222401P09 full insert sequence | unknown |
| 1 | IPI00118384 (IPI00325135) | 30.6 | 4 | 14-3-3 protein epsilon | unknown |
| 1 | IPI00408378 (PI00196661) | 53.1 | 10 | 14-3-3 protein tau | cytoplasm |
| 1 | IPI00324893 (PI00116498) | 35.1 | 9 | 14-3-3 protein zeta\delta | mt |
| 1 | IPI00133066 | 12.9 | 4 | 1500002F15Rik protein | unknown |
| 1 | IPI00119131 | 40.3 | 20 | 1500005G05Rik protein | unknown |
| 1 | IPI00315815 (IPI00132350) | 17 | 3 | 1500009M05Rik protein | mt |
| 1 | IPI00119045 | 5.8 | 3 | 1500015B20Rik protein | unknown |
| 1 | IPI00123342 | 48.5 | 56 | 170 kda glucose regulated protein GRP170 precursor | unknown |
| 1 | IPI00118825 | 29.8 | 17 | 1700007H16Rik protein | unknown |
| 0.99 | IPI00330987 | 34.6 | 2 | 1700008E09Rik protein | unknown |
| 1 | IPI00132478 | 11.7 | 2 | 1700021F05Rik protein | mt |
| 1 | IPI00112630 IPI00110721 | 11.5 | 2 | 1700082G03Rik protein 2700085E05Rik protein | mt |
| 1 | IPI00406550 IPI00314513 | 13.8 | 2 | 18 days embryo cDNA RIKEN full-length enriched library clone:1110025A19 full insert sequence Similar to hypothetical protein AB030201 | unknown |
| 1 | IPI00132050 IPI00110825 | 25.8 | 8 | 1810004I06Rik protein 2010300P09Rik protein | unknown |
| 1 | IPI00315763 | 26.3 | 4 | 1810013M05Rik protein | unknown |
| 1 | IPI00379695 (IPI00111816) | 22.3 | 7 | 1810014G04Rik protein | mt |
| 1 | IPI00112227 | 51 | 14 | 1810020E01Rik protein | mt |
| 1 | IPI00135208 IPI00205193 | 26.9 | 5 | 1810033A19Rik protein Ensembl_locations(Chr-bp):10-90321502 | unknown |
| 1 | IPI00318935 IPI00115304 | 12 | 2 | 1810035L17Rik protein Hypothetical protein | mt |
| 1 | IPI00315794 (PI00316026) | 59.6 | 14 | 1810044O22Rik protein | unknown |
| 1 | IPI00119715 IPI00387258 IPI00362392 | 11.6 | 3 | 1810049A15Rik protein ER protein 58 Similar to ER protein 58 | mt |
| 1 | IPI00133187 | 35.3 | 3 | 1810055D05Rik protein | nuleus |
| 1 | IPI00126548 | 24.6 | 4 | 1-acyl-sn-glycerol-3-phosphate acyltransferase alpha | mt |
| 1 | IPI00318645 | 58.7 | 4 | 2010012C24Rik protein | unknown |
| 1 | IPI00129519 | 26.5 | 2 | 22 kda neuronal tissue-enriched acidic protein | mt |
| 1 | IPI00132347 | 55 | 14 | 2210415M14Rik protein | unknown |
| 1 | IPI00132218 IPI00373430 | 25.5 | 2 | 2310008M10Rik protein Similar to RIKEN cDNA 2310008M10 | mt |
| 0.98 | IPI00153903 IPI00358095 | 9.5 | 2 | 2310014M14Rik protein Similar to citrate lyase beta like | unknown |
| 1 | IPI00225288 | 27.3 | 6 | 2310015N07Rik protein | unknown |
| 1 | IPI00110100 | 28 | 6 | 2310016N05Rik protein | unknown |
| 1 | IPI00132039 | 37.5 | 16 | 2310034D24Rik protein | unknown |
| 1 | IPI00109611 | 18.7 | 6 | 2310056P07Rik protein | mt |
| 1 | IPI00109501 | 32.8 | 7 | 2400002D02Rik protein | unknown |
| 0.98 | IPI00109744 | 11.9 | 2 | 2410001E19Rik protein | nuleus |
| 0.9 | IPI00109632 | 20.2 | 2 | 2410003K15Rik protein | unknown |
| 1 | IPI00331436 | 45.7 | 20 | 2410015L10Rik protein | unknown |
| 1 | IPI00109313 | 6.7 | 2 | 2410015L18Rik protein | mt |
| 1 | IPI00131900 IPI00370438 | 12.7 | 2 | 2610001E06Rik protein Similar to RIKEN cDNA 2610001E06 | cytoplasm |
| 1 | IPI00132653 | 53.5 | 44 | 2610008O03Rik protein | unknown |
| 1 | IPI00315511 IPI00387438 | 20.2 | 4 | 2610008O20Rik protein Pex13p protein | unknown |
| 1 | IPI00132529 | 31.5 | 13 | 2610017G09Rik protein | unknown |
| 1 | IPI00134420 (IPI00135203) | 26.8 | 7 | 2610020H15Rik protein | mt |
| 1 | IPI00132475 | 26.5 | 8 | 2610020P13Rik protein | mt |
| 0.72 | IPI00132453 | 38.4 | 2 | 2610021I23Rik protein | unknown |
| 1 | IPI00315437 | 44.1 | 3 | 2610021K23Rik protein | unknown |
| 1 | IPI00132443 IPI00209148 | 14.3 | 5 | 2610023M21Rik protein M4 protein | unknown |
| 1 | IPI00132419 | 5.9 | 3 | 2610024N01Rik protein | unknown |
| 1 | IPI00132487 IPI00210478 | 12.7 | 2 | 2610025P17Rik protein Similar to mitochondrial ribosomal protein 63 | mt |
| 1 | IPI00132042 | 47.4 | 42 | 2610103L06Rik protein | unknown |
| 1 | IPI00131792 | 13.4 | 3 | 2610209A20Rik protein | unknown |
| 1 | IPI00113262 | 25.2 | 3 | 2610312C03Rik protein | mt |
| 1 | IPI00315941 IPI00372833 (IPI00113243) | 19.4 | 3 | 2610318K02Rik protein Similar to RIKEN cDNA 2610318K02 | unknown |
| 1 | IPI00113141 | 55 | 36 | 2610511A05Rik protein | unknown |
| 1 | IPI00132170 (IPI00187247) | 22.7 | 2 | 2610511M02Rik protein | unknown |
| 1 | IPI00113137 IPI00113245 IPI00313547 | 17.7 | 3 | 2610524P08Rik protein Gtpase ERA-S Conserved ERA-like gtpase | mt |
| 1 | IPI00135640 (IPI00213587) | 9.4 | 2 | 26S protease regulatory subunit 8 | mt |
| 1 | IPI00313824 IPI00172368 | 18.4 | 3 | 27 kda Golgi SNARE protein SEC22 vesicle trafficking protein-like 1 | mt |
| 1 | IPI00113080 | 13.9 | 5 | 2700002I20Rik protein | unknown |
| 1 | IPI00113073 | 38 | 15 | 2700007F14Rik protein | unknown |
| 1 | IPI00133403 | 16.3 | 2 | 2700033I16Rik protein | mt |
| 1 | IPI00112320 IPI00387472 | 11.8 | 2 | 2700038L12Rik protein Meiotic recombination protein REC14 | mt |
| 1 | IPI00112937 | 14.3 | 4 | 2700038P16Rik protein | unknown |
| 1 | IPI00210219 IPI00313559 (IPI00132923) | 19.6 | 4 | 28 kda Golgi SNARE protein Golgi SNAP receptor complex member 1 | mt |
| 1 | IPI00134918 | 27.2 | 3 | 2810004N20Rik protein | mt |
| 1 | IPI00137460 | 16.5 | 4 | 2810037C14Rik protein | unknown |
| 1 | IPI00112232 IPI00336870 | 30.7 | 6 | 2810038N09Rik protein Similar to mitochondrial ribosomal protein L55 | mt |
| 1 | IPI00111045 | 28.9 | 12 | 2810403L02Rik protein | unknown |
| 1 | IPI00112032 | 54.2 | 8 | 2810407E01Rik protein | unknown |
| 1 | IPI00112128 | 14.7 | 3 | 2810435D12Rik protein | mt |
| 1 | IPI00321858 | 20.5 | 6 | 28S ribosomal protein S15 mitochondrial precursor | mt |
| 1 | IPI00132448 | 51.7 | 4 | 28S ribosomal protein S17 mitochondrial precursor | cytoplasm |
| 0.65 | IPI00153603 | 19.6 | 2 | 28S ribosomal protein S18c mitochondrial precursor | unknown |
| 1 | IPI00124828 | 24.5 | 6 | 28S ribosomal protein S31 mitochondrial precursor | mt |
| 1 | IPI00109165 (IPI00387430) | 21 | 2 | 2900010I05Rik protein | unknown |
| 1 | IPI00109047 (IPI00378120) | 27 | 6 | 2900070E19Rik protein | mt |
| 1 | IPI00136655 | 28.8 | 10 | 2-amino-3-ketobutyrate coenzyme A ligase mitochondrial precursor | mt |
| 1 | IPI00110456 | 22.9 | 2 | 3110082I17Rik protein | nucleus |
| 1 | IPI00110380 | 11.9 | 3 | 3230401I01Rik protein | unknown |
| 1 | IPI00130833 IPI00362623 | 25.9 | 6 | 38 kda FK-506 binding protein homolog Similar to FK506-binding protein 38 | mt |
| 0.99 | IPI00387389 | 11.7 | 2 | 39S ribosomal protein L18 mitochondrial precursor | unknown |
| 1 | IPI00271726 | 15.4 | 2 | 39S ribosomal protein L28 mitochondrial precursor | mt |
| 0.99 | IPI00132700 | 16 | 2 | 39S ribosomal protein L35 mitochondrial precursor | mt |
| 1 | IPI00323669 | 33.5 | 6 | 39S ribosomal protein L40 mitochondrial precursor | unknown |
| 1 | IPI00132895 | 29.1 | 7 | 39S ribosomal protein L45 mitochondrial precursor | mt |
| 1 | IPI00379694 | 32.9 | 8 | 3-hydroxy-3-methylglutaryl-coenzyme A lyase | unknown |
| 1 | IPI00330754 | 42 | 20 | 3-hydroxybutyrate dehydrogenase | mt |
| 1 | IPI00116222 | 34 | 13 | 3-hydroxyisobutyrate dehydrogenase mitochondrial precursor | mt |
| 1 | IPI00231106 | 27.9 | 4 | 3-mercaptopyruvate sulfurtransferase | mt |
| 1 | IPI00317590 IPI00387914 IPI00406666 (IPI00188058) | 32.9 | 4 | 40S ribosomal protein S18 Similar to ribosomal protein S18 cytosolic Ensembl_locations(Chr-bp):13-96203445 | mt |
| 1 | IPI00323819 IPI00359654 (IPI00327386) (IPI00212186) (IPI00360575) | 21.8 | 7 | 40S ribosomal protein S20 Similar to 40S ribosomal protein S20 | mt |
| 1 | IPI00212776 (IPI00134599) | 49.8 | 15 | 40S ribosomal protein S3 | mt |
| 1 | IPI00331092 IPI00367656 | 28.9 | 11 | 40S ribosomal protein S4 X isoform ribosomal protein S4 X-linked | mt |
| 1 | IPI00192486 IPI00108454 (IPI00381891) IPI00407081 IPI00113655 IPI00399797 | 15.7 | 5 | 40S ribosomal protein S6 Similar to 40S ribosomal protein S6 (Phosphoprotein NP33)  Ensembl_locations(Chr-bp):X-59209349 40S ribosomal protein S6 Ensembl_locations(Chr-bp):4-85580174 | mt |
| 1 | IPI00231202 IPI00399721 IPI00338564 IPI00406582 IPI00409485 (IPI00406504) IPI00407112 | 24.5 | 3 | 40S ribosomal protein S8 Ensembl_locations(Chr-bp):2-177624545 Ribosomal protein S8 Ensembl_locations(Chr-bp):10-58526306 Ensembl_locations(Chr-bp):2-175854634;2-176123594  Ensembl_locations(Chr-bp):13-26380361 | unknown |
| 1 | IPI00133240 | 35.4 | 17 | 4430402G14Rik protein | unknown |
| 0.93 | IPI00108520 | 9.9 | 2 | 4632434I11Rik protein | unknown |
| 1 | IPI00314069 | 23.7 | 4 | 48 kda acyl-coa thioester hydrolase mitochondrial precursor | mt |
| 1 | IPI00108410 | 41.3 | 14 | 4921525H23Rik protein | unknown |
| 0.99 | IPI00108118 IPI00319986 | 13 | 2 | 4930418P06Rik protein Similar to RIKEN cDNA 4930418P06 gene | unknown |
| 1 | IPI00134809 IPI00365865 | 28.2 | 15 | 4930529O08Rik protein Similar to dihydrolipoamide S-succinyltransferase (E2 component of 2-oxo-glutarate complex) | unknown |
| 1 | IPI00330741 | 5 | 3 | 4933425L03Rik protein | unknown |
| 1 | IPI00114641 | 37.1 | 20 | 4F2 cell-surface antigen heavy chain | mt |
| 1 | IPI00124372 | 34.2 | 11 | 4-trimethylaminobutyraldehyde dehydrogenase | cytoplasm |
| 1 | IPI00136285 IPI00126855 | 27.5 | 4 | 5230400G24Rik protein Similar to RIKEN cDNA 5230400G24 gene | mt |
| 1 | IPI00110999 | 33.5 | 8 | 5730591C18Rik protein | mt |
| 1 | IPI00318453 (IPI00119024) | 10.1 | 2 | 5930404D22Rik protein | mt |
| 1 | IPI00210584  IPI00393333 | 15.5 | 5 | 5-aminoimidazole-4-carboxamide ribonucleotide formyltransferase\IMP cyclohydrolase Ensembl_locations(Chr-bp):9-70823685 | membrane |
| 1 | IPI00113143 | 13.8 | 2 | 6.8 kda mitochondrial proteolipid | mt |
| 1 | IPI00308885 (IPI00339148) | 79.1 | 30 | 60 kda heat shock protein mitochondrial precursor | mt |
| 1 | IPI00133608 | 49.4 | 4 | 6030432N09Rik protein | mt |
| 1 | IPI00314950 | 52.4 | 12 | 60S acidic ribosomal protein P0 | mt |
| 1 | IPI00113377 | 66.7 | 6 | 60S acidic ribosomal protein P1 | cytoplasm |
| 1 | IPI00132470 | 34.9 | 5 | 60S ribosomal protein L11 mitochondrial precursor | mt |
| 1 | IPI00118963 | 49.3 | 28 | 60S ribosomal protein L12 mitochondrial precursor | mt |
| 1 | IPI00134011 IPI00366411 | 24.2 | 5 | 60S ribosomal protein L13 mitochondrial Similar to mitochondrial ribosomal protein L13 | mt |
| 1 | IPI00162790 (IPI00192257) | 26.1 | 5 | 60S ribosomal protein l18a | cytoplasm |
| 1 | IPI00135311 | 36.3 | 8 | 60S ribosomal protein L19 mitochondrial precursor | mt |
| 1 | IPI00207980 (IPI00139780) IPI00379530 | 32.9 | 8 | 60S ribosomal protein L23   Similar to ribosomal protein L23 | mt |
| 1 | IPI00120955 | 16 | 2 | 60S ribosomal protein L32 mitochondrial precursor | mt |
| 1 | IPI00115902 IPI00369804 (IPI00115233) IPI00279674 IPI00400634 IPI00197720 (IPI00379192) (IPI00378074) (IPI00403796) IPI00400492 (IPI00359889) (IPI00381934) (IPI00368733) IPI00392735 | 9.1 | 4 | 60S ribosomal protein l35a Similar to ribosomal protein l35a  Ensembl_locations(Chr-bp):13-6474687 Ensembl_locations(Chr-bp):17-16591884 60S ribosomal protein l35a  Ensembl_locations(Chr-bp):11-69608028  Ensembl_locations(Chr-bp):1-54491656 | cytoplasm |
| 1 | IPI00331121 IPI00279867 IPI00387976 IPI00338977 IPI00394050 IPI00138302 (IPI00390823)IPI00194092 | 31.9 | 4 | 60S ribosomal protein L38 Ensembl_locations(Chr-bp):11-114174135 Ensembl_locations(Chr-bp):10-104560466 Ensembl_locations(Chr-bp):4-137203599 Ensembl_locations(Chr-bp):X-92024120 Ensembl_locations(Chr-bp):6-52816053  Ensembl_locations(Chr-bp):4-59072803 | unknown |
| 1 | IPI00111412 | 35.1 | 21 | 60S ribosomal protein L4 | mt |
| 1 | IPI00403448 (IPI00230914)(IPI00308706) | 32.4 | 16 | 60S ribosomal protein L5 | mt |
| 1 | IPI00313222 | 24.7 | 11 | 60S ribosomal protein L6 | unknown |
| 1 | IPI00311236 | 35.9 | 4 | 60S ribosomal protein L7 | mt |
| 1 | IPI00122413 | 54.7 | 3 | 60S ribosomal protein L9 | unknown |
| 1 | IPI00231954 | 14.6 | 5 | 6-phosphofructokinase type C | mt |
| 1 | IPI00320400 IPI00382191 | 12.4 | 2 | 6-phosphogluconate dehydrogenase decarboxylating Cc2-27 | mt |
| 1 | IPI00206624 (IPI00319992) | 44.6 | 66 | 78 kda glucose-regulated protein precursor | mt |
| 1 | IPI00130988 | 7.4 | 3 | 7-dehydrocholesterol reductase | mt |
| 1 | IPI00135191 | 15.1 | 3 | 9030624B09Rik protein | mt |
| 1 | IPI00112923 | 46.8 | 5 | 9430083G14Rik protein | unknown |
| 1 | IPI00382252 | 19.4 | 5 | Aa2-111 | unknown |
| 0.61 | IPI00382330 | 8.5 | 2 | Ac1164 | unknown |
| 1 | IPI00364884 IPI00110852 | 18.9 | 4 | Ac2-238 Translocon-associated protein alpha | mt |
| 1 | IPI00121833 | 17.4 | 4 | Acetyl-Coenzyme A acyltransferase 1 | unknown |
| 1 | IPI00331251 | 32.5 | 15 | Acetyl-coenzyme A dehydrogenase short chain | unknown |
| 1 | IPI00125266 | 11.9 | 3 | Acid ceramidase precursor | mt |
| 1 | IPI00138338 | 21.7 | 5 | Actin related protein 2\3 complex subunit 2 | mt |
| 1 | IPI00177038 IPI00400012 (IPI00362072) | 19 | 6 | Actin-like protein 2 Similar to Actin-like protein 2 (Actin-related protein 2) | mt |
| 1 | IPI00153740 | 9.8 | 2 | Activator of 90 kda heat shock protein atpase homolog 1 | cytoplasm, ER |
| 1 | IPI00133006 IPI00191112 | 15.4 | 6 | Acyl carrier protein mitochondrial precursor Similar to Acyl carrier protein mitochondrial precursor (ACP) (NADH-ubiquinone oxidoreductase 9.6 kda subunit) (CI-SDAP) | mt |
| 1 | IPI00136683 | 30.2 | 11 | Acyl coenzyme A thioester hydrolase mitochondrial precursor | mt |
| 0.99 | IPI00274222 IPI00230163 | 11.4 | 3 | Acyl-Coa dehydrogenase family member 8 mitochondrial precursor Acyl-Coenzyme A dehydrogenase family member 8 | unknown |
| 1 | IPI00331710 | 37.3 | 20 | Acyl-coa dehydrogenase family member 9 mitochondrial precursor | mt |
| 1 | IPI00211225 | 40.2 | 29 | Acyl-coa dehydrogenase long-chain specific mitochondrial precursor | mt |
| 1 | IPI00212015  (IPI00134961) | 30.9 | 12 | Acyl-coa dehydrogenase medium-chain specific mitochondrial precursor | mt |
| 1 | IPI00119842 | 19 | 9 | Acyl-coa dehydrogenase short\branched chain specific mitochondrial precursor | mt |
| 1 | IPI00119203 | 38.3 | 20 | Acyl-coa dehydrogenase very-long-chain specific mitochondrial precursor | mt |
| 1 | IPI00119114 | 37.9 | 34 | Acyl-coa dehydrogenaselong-chain specific mitochondrial precursor | mt |
| 1 | IPI00262693 IPI00123390 | 8.5 | 2 | Acyl-coa synthetase long-chain family member 6 Long-chain-fatty-acid--coa ligase 6 | mt |
| 0.76 | IPI00128918 | 5.9 | 2 | Ad24 | unknown |
| 1 | IPI00346965 | 16.5 | 4 | Adapter-related protein complex 1 beta 1 subunit | mt |
| 1 | IPI00389753 (PI00231502)  IPI00119689 IPI00378063 | 15.6 | 9 | Adapter-related protein complex 2 beta 1 subunit  1300012O03Rik protein  Ap2b1 protein | mt |
| 1 | IPI00231397 IPI00404674 | 4.2 | 2 | Adaptor-related protein complex 2 alpha 2 subunit Adaptor protein complex AP-2 | golgi |
| 1 | IPI00128491 | 62.2 | 4 | Adenine phosphoribosyltransferase | cytoplasm |
| 0.72 | IPI00188859 | 4.3 | 2 | Adenomatous polyposis coli protein | unknown |
| 0.71 | IPI00127551 IPI00359353 | 6.2 | 2 | Adenylate cyclase type VII Similar to adenylyl cyclase type VII | unknown |
| 1 | IPI00269076 | 44.4 | 6 | Adenylate kinase 2 | mt |
| 1 | IPI00230857 | 45.2 | 5 | Adenylate kinase 2 | mt |
| 1 | IPI00221769 IPI00362243 | 33.5 | 7 | Adenylate kinase 3 alpha-like Hypothetical protein XP_346582 | mt |
| 1 | IPI00125035 IPI00405342 | 39.5 | 7 | Adenylate kinase isoenzyme 4 mitochondrial Ensembl_locations(Chr-bp):4-100279459 | mt |
| 1 | IPI00137331 | 16.2 | 3 | Adenylyl cyclase-associated CAP protein homolog 1 | mt |
| 1 | IPI00221998 | 18 | 6 | Adipocyte plasma membrane-associated protein | membrane |
| 1 | IPI00310518 | 38.7 | 25 | Adipocyte-specific protein 4 | mt |
| 1 | IPI00407383 | 21.3 | 5 | ADIR1 precursor | cytoplasm |
| 1 | IPI00127841 (IPI00200466) | 51.7 | 9 | ADP ATP carrier protein fibroblast isoform | mt |
| 1 | IPI00115564 | 59.1 | 22 | ADP ATP carrier protein heart\skeletal muscle isoform T1 | mt |
| 1 | IPI00231927 | 54.7 | 14 | ADP ATP carrier protein heart\skeletal muscle isoform T1 | mt |
| 1 | IPI00221614 IPI00331953 (IPI00231674)(IPI00221613) | 54.7 | 16 | ADP-ribosylation factor 3 ADP-ribosylation factor 1 | mt |
| 1 | IPI00135071 | 14.4 | 3 | ADP-ribosylation factor-like membrane-associated protein | mt |
| 1 | IPI00278498 (IPI00191467) | 37 | 3 | ADP-ribosylation factor-like protein 1 | mt |
| 1 | IPI00408291  IPI00127431 IPI00403637 | 41.5 | 3 | Adult female placenta cDNA RIKEN full-length enriched library clone:1600032G08 full insert sequence Cytoplasmic dynein light chain Ensembl_locations(Chr-bp):5-112048094 | mt |
| 1 | IPI00113386 | 31.1 | 10 | Adult male kidney cDNA RIKEN full-length enriched library clone:0610025L15 product:protein expressed in thyroid homolog | mt |
| 1 | IPI00126042  IPI00196794 | 51.6 | 10 | Adult male kidney cDNA RIKEN full-length enriched library clone:0610030G24 full insert sequence Ras-related protein Rab-14 | mt |
| 1 | IPI00136691 | 12.7 | 2 | Adult male testis cDNA RIKEN full-length enriched library clone:4930565F05 product:hypothetical alanine-rich region containing protein full insert sequence | unknown |
| 1 | IPI00331490 IPI00271562 | 13.1 | 3 | Aflatoxin B1 aldehyde reductase member 2 Ensembl_locations(Chr-bp):4-138183469 | mt |
| 0.96 | IPI00408638 | 12.3 | 2 | Age-related protein | unknown |
| 1 | IPI00311509 | 13.2 | 4 | Aladin | mt |
| 0.71 | IPI00213584 | 9.6 | 2 | Alanine--glyoxylate aminotransferase 2 mitochondrial precursor | unknown |
| 1 | IPI00226805 IPI00321308 | 12.3 | 8 | Alanyl-tRNA synthetase Similar to alanyl-tRNA synthetase | mt |
| 0.63 | IPI00318925 | 4.5 | 2 | Alcohol dehydrogenase 6A (class V) | unknown |
| 1 | IPI00111218 | 41.6 | 17 | Aldehyde dehydrogenase mitochondrial precursor | mt |
| 1 | IPI00221402 | 34.1 | 11 | Aldolase 1 A isoform | unknown |
| 0.71 | IPI00309437 | 5.7 | 2 | ALDR protein | unknown |
| 1 | IPI00223818 | 41.9 | 18 | Alkyl-DIHYDROXYACETONEPHOSPHATE synthase homolog | unknown |
| 1 | IPI00115679 IPI00403058 | 36.5 | 33 | Alpha glucosidase II alpha subunit Alpha glucosidase 2 | ER |
| 1 | IPI00115680 IPI00122353 IPI00388209 (IPI00388122) IPI00372446 (IPI00392603) | 8.5 | 3 | Alpha glucosidase II beta subunit Similar to protein kinase C substrate 80K-H Ensembl_locations(Chr-bp):8-21107428  Similar to alpha glucosidase II beta subunit | ER, Glogi |
| 1 | IPI00118899 IPI00409205 (IPI00120382) (IPI00409309) | 18.3 | 8 | Alpha-actinin 4 Ensembl_locations(Chr-bp):7-22652897 | mt |
| 0.85 | IPI00113163 | 11.1 | 3 | Alpha-fetoprotein precursor | unknown |
| 1 | IPI00118930 (IPI00189925) | 36.9 | 8 | Alpha-soluble NSF attachment protein | mt |
| 1 | IPI00137725 | 18.5 | 3 | Ancient ubiquitous protein 1 precursor | mt |
| 1 | IPI00317309 | 9.1 | 2 | Annexin A5 | mt |
| 1 | IPI00310240 | 12 | 6 | Annexin A6 | mt |
| 1 | IPI00200918 IPI00224152 (IPI00231964) | 12.1 | 2 | APEX Apurinic\apyrimidinic endonuclease 1 | mt |
| 1 | IPI00120684 (IPI00211129) | 38.5 | 5 | Apoptosis regulator BAX membrane isoform alpha | mt |
| 0.9 | IPI00229434 | 4.8 | 2 | Apoptosis stimulating of p53 protein 2 | mt |
| 1 | IPI00315488 | 23.3 | 11 | Arginyl-tRNA synthetase | mt |
| 1 | IPI00116966 | 20 | 6 | Asparagine synthetase | nucleus |
| 1 | IPI00117312 | 61.6 | 42 | Aspartate aminotransferase mitochondrial precursor | mt |
| 1 | IPI00224890 IPI00407284 | 7.8 | 2 | Aspartate-beta-hydroxylase Aspartly beta-hydroxylase | mt |
| 1 | IPI00206224 (IPI00222457) (IPI00122743) | 15.8 | 3 | Aspartyl-tRNA synthetase | mt |
| 1 | IPI00123202 | 25.5 | 4 | Ataxin-10 | mt |
| 1 | IPI00130280 | 58.6 | 79 | ATP synthase alpha chain mitochondrial precursor | mt |
| 1 | IPI00341282 | 57 | 29 | ATP synthase B chain mitochondrial precursor | mt |
| 1 | IPI00196107 | 54.3 | 20 | ATP synthase B chain mitochondrial precursor | mt |
| 1 | IPI00113801 | 77.3 | 116 | ATP synthase beta chain mitochondrial precursor | mt |
| 1 | IPI00193214 | 77 | 110 | ATP synthase beta chain mitochondrial precursor | mt |
| 1 | IPI00204316 | 21.3 | 3 | ATP synthase coupling factor 6 mitochondrial precursor | mt |
| 1 | IPI00125460 | 26.9 | 2 | ATP synthase coupling factor 6 mitochondrial precursor | mt |
| 1 | IPI00271986 IPI00345136 | 28 | 7 | ATP synthase f chain mitochondrial Ensembl_locations(Chr-bp):2-18756080 | mt |
| 1 | IPI00313475 | 31.5 | 5 | ATP synthase gamma chain mitochondrial precursor | unknown |
| 1 | IPI00331316 | 48.5 | 13 | ATP synthase H+ transporting mitochondrial F0 complex subunit g | mt |
| 1 | IPI00396906 | 31.5 | 7 | ATP synthase H+ transporting mitochondrial F1 complex gamma polypeptide 1 | mt |
| 1 | IPI00111770 | 52.1 | 4 | ATP synthase H+ transporting mitochondrial F1F0 complex subunit e | mt |
| 1 | IPI00231978 | 33.8 | 2 | ATP synthase H+ transporting mitochondrial F1F0 complex subunit e | mt |
| 1 | IPI00195123 | 59.2 | 19 | ATP synthase oligomycin sensitivity conferral protein mitochondrial precursor | mt |
| 1 | IPI00116896 | 34.3 | 3 | ATP synthase protein 8 | mt |
| 1 | IPI00311682 IPI00326305 | 28.3 | 24 | Atpase Na+K+ transporting alpha 1 polypeptide Sodium\potassium-transporting atpase alpha-1 chain precursor | membrane |
| 1 | IPI00381395 | 25.3 | 8 | ATP-binding cassette sub-family B (MDR\TAP) member 7 | mt |
| 1 | IPI00231860 IPI00362476 | 16.6 | 5 | ATP-binding cassette sub-family D (ALD) member 3 Hypothetical protein XP_346639 | mt |
| 1 | IPI00116825 IPI00213162 | 6.1 | 3 | ATP-binding cassette sub-family F member 2 Similar to ATP-binding cassette sub-family F (GCN20) member 2 | mt |
| 0.67 | IPI00265219 | 2.8 | 2 | ATP-binding cassette transporter | unknown |
| 1 | IPI00119808 | 28.7 | 16 | ATP-dependent Clp protease ATP-binding subunit clpx-like mitochondrial precursor | mt |
| 0.98 | IPI00132424 | 4.4 | 2 | ATP-dependent DNA helicase II 70 kda subunit | nuleus |
| 1 | IPI00136555 | 30.1 | 21 | ATP-dependent metalloprotease ftsh1 | mt |
| 1 | IPI00206065 | 30.8 | 21 | ATP-dependent metalloprotease ftsh1 homolog | mt |
| 0.76 | IPI00194733 | 5.1 | 2 | Attractin | unknown |
| 0.64 | IPI00326141 | 27.9 | 2 | Augmenter of liver regeneration | unknown |
| 0.86 | IPI00382204 | 3.5 | 2 | Ba1-651 | unknown |
| 1 | IPI00407222 | 7.7 | 4 | Ba207c16.3 | unknown |
| 1 | IPI00321718 | 45.8 | 37 | Bap | mt |
| 1 | IPI00119959 | 27 | 3 | Barrier-to-autointegration factor | mt |
| 0.91 | IPI00113824 | 4 | 4 | Basement membrane-specific heparan sulfate proteoglycan core protein precursor | mt |
| 1 | IPI00113869 IPI00407948 IPI00408495 | 22 | 5 | Basigin precursor Basigin Basigin 2 | mt |
| 0.64 | IPI00212553 IPI00363017 | 2.2 | 3 | Bassoon Hypothetical protein XP_346835 | unknown |
| 1 | IPI00230422 | 21.6 | 8 | B-cell receptor-associated protein 31 | mt |
| 1 | IPI00309183 IPI00202077 IPI00215557 IPI00314275 | 24.5 | 7 | Bcl-2 homologous antagonist\killer BAK protein Ensembl_locations(Chr-bp):20-5257241 N-BAK1 | mt |
| 1 | IPI00321499 | 13.4 | 4 | Bcl-2-like protein 13 | mt |
| 1 | IPI00132685 (IPI00209329) | 24.6 | 2 | BET1 homolog | mt |
| 0.99 | IPI00231953 IPI00115097 | 5.5 | 2 | Beta prime COP Coatomer beta' subunit | mt |
| 1 | IPI00227445 IPI00407499 | 15.3 | 6 | Beta-alanine oxoglutarate aminotransferase 4-aminobutyrate aminotransferase | unknown |
| 0.61 | IPI00405221 IPI00311335 | 7.6 | 2 | Bifunctional 3'-phosphoadenosine 5'-phosphosulfate synthethase 1 Ensembl_locations(Chr-bp):3-135161184 | unknown |
| 1 | IPI00109824 | 69.7 | 46 | Bifunctional methylenetetrahydrofolate dehydrogenase\cyclohydrolase mitochondrial precursor | mt |
| 0.93 | IPI00396778 (IPI00318545) | 8.4 | 2 | Bisphosphate 3'-nucleotidase 1 | unknown |
| 0.82 | IPI00223929 IPI00153107 | 20.2 | 2 | Bleomycin hydrolase Similar to bleomycin hydrolase | unknown |
| 1 | IPI00122826 | 44.3 | 2 | BM88 antigen | mt |
| 0.67 | IPI00400618 | 3.9 | 2 | Bone specific CMF608 | unknown |
| 1 | IPI00322760 | 9.4 | 4 | Brain and kidney proline oxidase 2 | mt |
| 1 | IPI00404997 | 21.4 | 2 | Brain cDNA clone mncb-2844 Similar to Mus musculus mcasp (cux) mRNA | unknown |
| 0.77 | IPI00121814 IPI00403784 | 6.5 | 2 | Brain cDNA clone mncb-4931 Similar to X96994 BR-1 protein Brain CDNA | unknown |
| 0.99 | IPI00203604 IPI00402961 (IPI00124292) | 57.1 | 2 | Brain protein 44-like protein Ensembl_locations(Chr-bp):12-106426680 | mt |
| 1 | IPI00365663 IPI00115514 IPI00331555 | 31 | 5 | Branched chain keto acid dehydrogenase subunit E1 alpha polypeptide 2-oxoisovalerate dehydrogenase alpha subunit mitochondrial precursor Branched chain ketoacid dehydrogenase E1 alpha polypeptide | mt |
| 1 | IPI00115302 IPI00404065 IPI00201636 | 34.7 | 2 | Branched chain ketoacid dehydrogenase E1 beta polypeptide Ensembl_locations(Chr-bp):9-86709579 2-oxoisovalerate dehydrogenase beta subunit mitochondrial precursor | mt |
| 1 | IPI00324180 IPI00366614 | 4.6 | 6 | Breast cancer type 2 susceptibility protein homolog Breast cancer susceptibility protein BRCA2 | unknown |
| 1 | IPI00122862 | 13.9 | 6 | C1-tetrahydrofolate synthase | mt |
| 1 | IPI00229680 IPI00207794 (IPI00394419) | 24.9 | 4 | Calcium binding protein P22 Ensembl_locations(Chr-bp):3-105962571 | mt |
| 1 | IPI00135651 | 44.1 | 16 | Calcium-binding mitochondrial carrier protein Aralar2 | mt |
| 1 | IPI00115650 | 29.1 | 11 | Calcyclin binding protein | mt |
| 1 | IPI00231955 IPI00110986 | 31.1 | 5 | Calmodulin 3 Calmodulin | mt |
| 1 | IPI00119618 | 19.1 | 16 | Calnexin precursor | mt |
| 1 | IPI00123639 | 45.2 | 22 | Calreticulin precursor | ER |
| 1 | IPI00135186 | 42.5 | 14 | Calumenin precursor | ER |
| 0.66 | IPI00396891 IPI00221980 IPI00211755 (IPI00403153) IPI00261641 | 13.4 | 2 | Camp-specific cyclic nucleotide phosphodiesterase PDE8 Similar to high-affinity camp-specific and IBMX-insensitive 3' Ensembl_locations(Chr-bp):2-25273202  Similar to phosphodiesterase | unknown |
| 1 | IPI00111265 IPI00370681 | 45.1 | 6 | Capping protein (actin filament) muscle Z-line alpha 2 Similar to capping protein alpha 2 subunit | mt |
| 1 | IPI00131424 | 17.3 | 6 | Carnitine O-palmitoyltransferase II mitochondrial precursor | Cytoplasm |
| 1 | IPI00195593 | 7.8 | 2 | Carnitine O-palmitoyltransferase II mitochondrial precursor | Cytoplasm |
| 1 | IPI00330094 | 7.8 | 4 | Carnitine palmitoyltransferase 1 | mt |
| 1 | IPI00192586 IPI00320479 IPI00408176 | 36.1 | 17 | Casein kinase II alpha chain Casein kinase II alpha 1 related sequence 4 Casein kinase II alpha subunit | mt |
| 1 | IPI00118795 IPI00188618 | 15.1 | 4 | Casein kinase II alpha' chain Similar to casein kinase II alpha prime subunit | mt |
| 1 | IPI00126762 (IPI00205531) | 38.1 | 7 | Casein kinase II beta chain | mt |
| 1 | IPI00314106 | 16.9 | 4 | Cat eye syndrome critical region protein 5 homolog precursor | unknown |
| 1 | IPI00312058 | 41 | 26 | Catalase 1 | Peroxisome |
| 1 | IPI00212811 (IPI00113517)IPI00361928 | 15.9 | 3 | Cathepsin B precursor  Hypothetical protein XP_346477 | cytoplasm |
| 1 | IPI00111013 IPI00404551 | 21.7 | 7 | Cathepsin D precursor Cathepsin D | unknown |
| 0.69 | IPI00196054 (PI00125256) | 9.9 | 2 | C-C chemokine receptor type 2 | mt |
| 1 | IPI00127447 | 9 | 3 | CD36 antigen | membrane |
| 1 | IPI00187902 IPI00221921 | 19 | 3 | CD9 antigen (p24) CD9 antigen | mt |
| 1 | IPI00285606 IPI00113849 | 15.7 | 3 | CDC42 protein Splice isoform 2 of P21181 Cell division control protein 42 homolog | mt |
| 1 | IPI00117025 | 5.6 | 4 | CDK5 regulatory subunit associated protein 3 | unknown |
| 1 | IPI00308184 | 11.1 | 4 | CDNA flj10375 fis | mt |
| 1 | IPI00225318 | 26.2 | 6 | CDNA flj20594 fis | mt |
| 1 | IPI00340241 IPI00189691 | 8.6 | 2 | CDNA FLJ30553 FIS Similar to protein phosphatase 2C epsilon | mt |
| 1 | IPI00211134 IPI00124240 IPI00225350 IPI00391356 (PI00391263) | 16.1 | 2 | Cell division protein kinase 2 Splice isoform CDK2-beta of P97377 Cell division protein kinase 2 Splice isoform CDK2-alpha of P97377 Cell division protein kinase 2 Ensembl_locations(Chr-bp):7-2001086 | mt |
| 0.98 | IPI00399663 | 4.7 | 4 | Centromere protein E | unknown |
| 1 | IPI00125771 | 18.9 | 9 | Cervical cancer receptor | mt |
| 1 | IPI00372388 | 16.1 | 5 | Chaperonin containing TCP1 subunit 3 (gamma) | unknown |
| 0.99 | IPI00130344 IPI00367621 | 19.4 | 2 | Chloride intracellular channel protein 1 Similar to CLCP | mt |
| 1 | IPI00135977 | 18.2 | 2 | Chloride intracellular channel protein 4 | mt |
| 1 | IPI00198080 | 19.4 | 7 | Chloride ion pump-associated 55 kda protein | mt |
| 1 | IPI00206977 | 39.7 | 18 | Citrate synthase | mt |
| 1 | IPI00193983 IPI00169916 | 29.1 | 33 | Clathrin heavy chain clathrin Havy polypeptide (Hc) | cytoplasm |
| 1 | IPI00229834 IPI00372458 | 4.9 | 4 | Coatomer alpha subunit Similar to coatomer protein complex subunit alpha | mt |
| 0.71 | IPI00127100 | 13.8 | 3 | Cochlin precursor | unknown |
| 1 | IPI00407543 | 43.8 | 9 | Cofilin non-muscle isoform | Nuceus, cytoplasm |
| 0.89 | IPI00204991 | 5.6 | 2 | Collagen alpha1 | unknown |
| 0.89 | IPI00366945 | 6.4 | 2 | Collagen type V alpha 2 | unknown |
| 1 | IPI00135393 | 58.1 | 23 | Complement component 1 q subcomponent binding protein | mt |
| 0.95 | IPI00212452 | 55 | 2 | Complement component 1 Q subcomponent binding protein mitochondrial precursor | unknown |
| 0.93 | IPI00266752 | 5.8 | 2 | Copine III | mt |
| 1 | IPI00400301 | 29.9 | 5 | Coproporphyrinogen III oxidase mitochondrial precursor | mt |
| 0.97 | IPI00128647 | 8.3 | 3 | Crumbs-like protein 1 precursor | unknown |
| 0.61 | IPI00331507 (IPI00325517) | 7.8 | 2 | Cullin homolog 5 | unknown |
| 1 | IPI00197616 | 24.7 | 4 | Cyclase-associated protein homologue | mt |
| 1 | IPI00286154 (IPI00230113) IPI00231013 | 55.6 | 8 | Cytochrome b5   Splice isoform Short of P00173 | unknown |
| 1 | IPI00131176 | 27.8 | 17 | Cytochrome c oxidase polypeptide II | mt |
| 1 | (IPI00209908) | 27.8 | 15 |  | mt |
| 1 | IPI00192246 (IPI00120719) | 49.7 | 7 | Cytochrome c oxidase polypeptide Va mitochondrial precursor | mt |
| 1 | IPI00116154 | 23.4 | 8 | Cytochrome c oxidase polypeptide Vb mitochondrial precursor | mt |
| 0.91 | IPI00193918 | 23.3 | 2 | Cytochrome c oxidase polypeptide Vb mitochondrial precursor | unknown |
| 1 | IPI00117978 | 43.8 | 23 | Cytochrome c oxidase subunit IV isoform 1 mitochondrial precursor | mt |
| 1 | IPI00194222 | 39.1 | 10 | Cytochrome c oxidase subunit IV isoform 1 mitochondrial precursor | mt |
| 1 | IPI00121443 | 44.6 | 4 | Cytochrome c oxidase subunit VI a polypeptide 1 | mt |
| 1 | IPI00225390 IPI00389152 | 29.1 | 4 | Cytochrome c oxidase subunit vib Ensembl_locations(Chr-bp):2-204077027;1-85748168 | mt |
| 1 | IPI00120136 IPI00115949 | 48.6 | 4 | Cytochrome c oxidase subunit viia-related protein mitochondrial precursor Similar to silica-induced gene 81 | unknown |
| 1 | IPI00231864 (PI00222419) IPI00362298 (PI00265239) | 35.2 | 11 | Cytochrome c somatic   Similar to Cytochrome c somatic | mt |
| 1 | IPI00132728 | 39.4 | 37 | Cytochrome c1 heme protein mitochondrial precursor | mt |
| 1 | IPI00169542 | 36.2 | 21 | Cytochrome P450 51 | mt |
| 1 | IPI00353420 | 4 | 6 | Cytoskeletal protein | mt |
| 1 | IPI00198717 IPI00336324 | 18 | 5 | Cytosolic malate dehydrogenase Malate dehydrogenase cytoplasmic | mt |
| 1 | IPI00194680 | 21.2 | 3 | D-3-phosphoglycerate dehydrogenase | cytoplasm |
| 1 | IPI00109447 IPI00371540 | 14.4 | 2 | D530030D03Rik protein Similar to Protein HSPC163 | unknown |
| 1 | IPI00403272 IPI00313543 | 25.1 | 5 | D6Wsu176e protein Protein FAM3C precursor | unknown |
| 1 | IPI00382182 IPI00124684 IPI00407865 | 15.8 | 3 | Da2-35 Translin Ensembl_locations(Chr-bp):1-120842705 | mt |
| 1 | IPI00208175 (IPI00133234) | 9.3 | 3 | DB83 protein | mt |
| 0.94 | IPI00111497 | 12.1 | 3 | Ddm36e | mt |
| 1 | IPI00225183 | 21 | 16 | Dead | mt |
| 1 | IPI00230035 IPI00362534 | 21 | 11 | DEAD\H (Asp-Glu-Ala-Asp\His) box polypeptide 3 X-linked Similar to RNA helicase | unknown |
| 1 | IPI00188509 (IPI00109082) | 10.6 | 2 | Defender against cell death 1 | membrane |
| 1 | IPI00113731 | 5 | 2 | DEGENERATIVE spermatocyte homolog | unknown |
| 1 | IPI00331549 | 7.3 | 2 | Dehydrogenase\reductase SDR family member 1 | unknown |
| 1 | IPI00318750 | 32.7 | 8 | Dehydrogenase\reductase SDR family member 4 | mt |
| 1 | IPI00130804 | 21.4 | 4 | Delta35-delta24-dienoyl-coa isomerase mitochondrial precursor | mt |
| 1 | IPI00324109 IPI00387266 | 13.2 | 4 | Delta-5 fatty acid desaturase Delta-5 desaturase | Peroxisome |
| 1 | IPI00208106 (IPI00129362) | 12.8 | 5 | Delta-6 fatty acid desaturase | mt |
| 0.72 | IPI00321949 IPI00311808 | 8.7 | 2 | Dendritic cell-associated transmembrane protein Glycoprotein | unknown |
| 0.75 | IPI00130102 | 14.3 | 2 | Desmin | unknown |
| 1 | IPI00121343 | 5.5 | 3 | Desmoyokin | mt |
| 0.96 | IPI00403617 | 7.6 | 2 | Dexamethasone induced product | mt |
| 1 | IPI00120937 | 27 | 3 | Diablo homolog mitochondrial precursor | mt |
| 1 | IPI00222430 IPI00367951  IPI00108756 (IPI00231069) | 19.3 | 2 | Diazepam binding inhibitor Similar to acyl-Coa-binding protein (ACBP) (diazepam binding inhibitor) (DBI) (endozepine) (EP) Ensembl_locations(Chr-bp):11-53824322 | mt |
| 1 | IPI00373418 | 28.3 | 13 | Dihydrolipoamide branched chain transacylase E2 | mt |
| 1 | IPI00365545 | 46.4 | 23 | Dihydrolipoamide dehydrogenase (E3 component of pyruvate dehydrogenase complex 2-oxo-glutarate complex branched chain keto acid dehydrogenase complex) | Mt |
| 1 | IPI00115569 IPI00331564 | 49.3 | 28 | Dihydrolipoyl dehydrogenase mitochondrial precursor Dihydrolipoamide dehydrogenase | Mt |
| 1 | IPI00130733 | 46.8 | 18 | Dihydroorotate dehydrogenase mitochondrial precursor | ER |
| 0.99 | IPI00192034 (IPI00114375) | 11.5 | 2 | Dihydropyrimidinase related protein-2 | mt |
| 1 | IPI00126660 | 15.3 | 3 | Dihydroxyacetone phosphate acyltransferase | unknown |
| 0.94 | IPI00208648 | 5.1 | 2 | Disks large-associated protein 2 | mt |
| 0.66 | IPI00327963 IPI00390300 | 7.4 | 2 | DNA polymerase gamma subunit 1 Ensembl_locations(Chr-bp):1-135275359 | unknown |
| 1 | IPI00137628 | 22 | 3 | DNA polymerase gamma subunit 2 mitochondrial precursor | mt |
| 0.8 | IPI00378205 | 0.9 | 2 | DNA polymerase zeta catalytic subunit | unknown |
| 1 | IPI00133284 | 46.2 | 12 | DNA segment Chr 10 Johns Hopkins University 81 expressed | mt |
| 1 | IPI00118986 | 55.4 | 18 | DNA segment Chr 12 Wayne state University 28 expressed | unknown |
| 1 | IPI00131954 | 21.7 | 4 | DNA segment Chr 17 Wayne state University 104 expressed | mt |
| 1 | IPI00282823 IPI00229859 | 8.5 | 4 | DNA segment Chr 5 Wayne state University 45 expressed Similar to eukaryotic translation initiation factor 3 subunit 9 eta 116kda | cytoplasm |
| 1 | IPI00133965 | 38.8 | 8 | DNA segment Chr 9 Wayne state University 149 expressed | unknown |
| 1 | IPI00331598 | 23.9 | 4 | DNAj (Hsp40) homolog subfamily B member 12 | mt |
| 1 | IPI00314886 | 13.5 | 4 | DNAj (Hsp40) homolog subfamily C member 3 | mt |
| 1 | IPI00319790 IPI00331385 IPI00371053 | 5.7 | 2 | DNAj (Hsp40) homolog, subfamily C, member 7 Mdj11 Similar to DNAj (Hsp40) homolog, subfamily C, member 7 | mt |
| 1 | IPI00132208 (PI00210884) | 27.7 | 9 | DNAj homolog subfamily A member 1 | mt |
| 1 | IPI00136251 | 32.8 | 8 | DNAj homolog subfamily A member 2 | mt |
| 1 | IPI00320241 IPI00399789 | 33.8 | 10 | DNAj homolog subfamily B member 11 precursor Hypothetical protein | ER |
| 1 | IPI00331692 | 49.5 | 14 | Dodecenoyl-coenzyme A delta isomerase | mt |
| 1 | IPI00115668 | 42.7 | 13 | Dolichol-phosphate mannosyltransferase | mt |
| 1 | IPI00117705 | 51 | 25 | Dolichyl-diphosphooligosaccharide--protein glycosyltransferase 48 kda subunit precursor | ER |
| 1 | IPI00309035 | 50.2 | 41 | Dolichyl-diphosphooligosaccharide--protein glycosyltransferase 67 kda subunit precursor | ER |
| 1 | IPI00204365 | 44.8 | 37 | Dolichyl-diphosphooligosaccharide--protein glycosyltransferase 67 kda subunit precursor | ER |
| 1 | IPI00118995 IPI00372200 | 13.6 | 5 | Dolichyl-phosphate beta-glucosyltransferase Similar to dolichyl-phosphate beta-glucosyltransferase-like | mt |
| 1 | IPI00135708 IPI00407256 (IPI00231331) | 11.7 | 3 | Dual specificity mitogen-activated protein kinase kinase 2 Mitogen activated protein kinase kinase 2 | mt |
| 1 | IPI00120546 IPI00361396 | 21.3 | 2 | Dximx39e protein Similar to dximx39e protein | membrane |
| 0.93 | IPI00378501 | 1.8 | 2 | Dynein axonemal heavy polypeptide 9 | unknown |
| 1 | IPI00327630 | 14.1 | 44 | Dynein heavy chain cytosolic | mt |
| 1 | IPI00213552 IPI00153421 IPI00392968 | 6.6 | 2 | Dynein light intermediate chain 1 Hypothetical protein Ensembl_locations(Chr-bp):8-119123636 | cytoplasm |
| 1 | IPI00137424 | 30.6 | 8 | Ecsit | unknown |
| 0.91 | IPI00365661 | 3.5 | 4 | EGF-like-domain multiple 4 | unknown |
| 0.91 | IPI00117315 | 26.9 | 3 | Elastase 2 precursor | unknown |
| 1 | IPI00116753 | 47.7 | 21 | Electron transfer flavoprotein alpha-subunit mitochondrial precursor | unknown |
| 1 | IPI00205332 | 39.9 | 18 | Electron transfer flavoprotein alpha-subunit mitochondrial precursor | unknown |
| 1 | IPI00195372 (PI00307837)IPI00215281 | 34.2 | 13 | Elongation factor 1-alpha 1   Elongation factor-1 alpha | mt |
| 1 | IPI00318841 | 25.4 | 12 | Elongation factor 1-gamma | unknown |
| 1 | IPI00230283 | 25.6 | 7 | Elongation factor G 1 mitochondrial precursor | mt |
| 1 | IPI00113052 | 63.9 | 33 | Elongation factor Ts mitochondrial precursor | mt |
| 1 | IPI00274407 IPI00407246 | 47.1 | 36 | Elongation factor TU Ensembl_locations(Chr-bp):7-118631458 | mt |
| 1 | IPI00114401 | 29 | 4 | Emerin | mt |
| 1 | IPI00118832 | 42 | 11 | Endoplasmic reticulum protein erp29 precursor | ER |
| 1 | IPI00129526 | 40.8 | 64 | Endoplasmin precursor | mt |
| 1 | IPI00373432 | 25.7 | 6 | Endothelial monocyte activating polypeptide 2 | unknown |
| 1 | IPI00381603 | 35.5 | 10 | Enolase 1 alpha non-neuron | mt |
| 1 | IPI00221795 | 56.6 | 26 | Enoyl coenzyme A hydratase | mt |
| 1 | IPI00262113 IPI00202221 IPI00208657 IPI00377441 IPI00362101 IPI00213366 IPI00261455 IPI00324006 | 25.5 | 2 | Ensembl_locations(Chr-bp):10-131901891 Ensembl_locations(Chr-bp):X-91382409 Similar to 40S ribosomal protein S26 Ribosomal protein S26 Similar to 40S ribosomal protein S26 Ensembl_locations(Chr-bp):X-34345657 Ensembl_locations(Chr-bp):6-148168835 40S ribosomal protein S26 | mt |
| 0.97 | IPI00405338 | 18.6 | 2 | Ensembl_locations(Chr-bp):10-17371631 | unknown |
| 1 | IPI00392061 IPI00196264 (IPI00209003) IPI00391830 IPI00359314 (IPI00191912) IPI00118324 IPI00190240 IPI00137718 IPI00319539 IPI00221433 IPI00340936 (IPI00139518)IPI00198550 (IPI00391150) (IPI00361888) IPI00138892 IPI00380207 IPI00409051 | 44.6 | 5 | Ensembl_locations(Chr-bp):10-49441577 Polyubiquitin  Ensembl_locations(Chr-bp):1-241389197 Similar to ribosomal protein s27a  Ensembl_locations(Chr-bp):11-29444204 Ribosomal protein s27a Ubiquitin Polyubiquitin C Ribosomal protein s27a Similar to polyubiquitin  Ubiquitin A-52 residue ribosomal protein fusion product 1   Ubiquitin\60S ribosomal fusion protein Similar to ubiquitin A-52 residue ribosomal protein fusion product 1 Ensembl_locations(Chr-bp):19-16619920 | unknown |
| 1 | IPI00393815 IPI00307931 | 27.7 | 16 | Ensembl_locations(Chr-bp):10-68437180 Hypothetical EF-hand\small gtpase | unknown |
| 1 | IPI00390786 (PI00190407) | 22.7 | 10 | Ensembl_locations(Chr-bp):10-88200227 | unknown |
| 1 | IPI00114411 IPI00389238 (IPI00110477) IPI00380851 (IPI00365986) | 13.5 | 4 | Ensembl_locations(Chr-bp):10-89125366 Ensembl_locations(Chr-bp):7-23429833  Similar to TU12B1-TY protein | mt |
| 1 | IPI00212082 IPI00368435 IPI00157497 IPI00351867 | 3.5 | 3 | Ensembl_locations(Chr-bp):1-153887103 Similar to Ten-m4 Ten-m4 Odd Oz\ten-m homolog 4 | mt |
| 1 | IPI00119211 IPI00323806 (IPI00230939) | 24.8 | 5 | Ensembl_locations(Chr-bp):11-58106708 60S ribosomal protein L24 | unknown |
| 1 | IPI00408700 IPI00122215 | 13.2 | 2 | Ensembl_locations(Chr-bp):1-159465460 Hypothetical protein | mt |
| 1 | IPI00117354 IPI00390867 | 18.2 | 4 | Ensembl_locations(Chr-bp):11-66623937 Ensembl_locations(Chr-bp):10-53766072 | unknown |
| 1 | IPI00338838 IPI00353099 | 54.5 | 6 | Ensembl_locations(Chr-bp):11-82599542 60S ribosomal protein L12 | unknown |
| 1 | IPI00127886 (IPI00128785) IPI00122633 | 12.5 | 4 | Ensembl_locations(Chr-bp):11-94140775  Similar to hypothetical protein FLJ20920 | unknown |
| 1 | IPI00408829 IPI00284914 IPI00231229 IPI00283531 | 9.5 | 2 | Ensembl_locations(Chr-bp):1-195858884 Glutathione S-transferase P 1 Glutathione S-transferase P Glutathione S-transferase P 2 | mt |
| 0.66 | IPI00389440  IPI00138199 | 3.9 | 2 | Ensembl_locations(Chr-bp):1-221126616 Splice isoform PC5B of Q04592 Proprotein convertase subtilisin\kexin type 5 precursor | unknown |
| 0.68 | IPI00339677 | 8.6 | 2 | Ensembl_locations(Chr-bp):12-30473377 | unknown |
| 0.92 | IPI00388691 IPI00368204 | 4.7 | 2 | Ensembl_locations(Chr-bp):1-239059520 Similar to mitotic kinesin-related protein | unknown |
| 1 | IPI00388015 IPI00371227 IPI00311185 IPI00124820 | 22.6 | 5 | Ensembl_locations(Chr-bp):12-43638436 Similar to slingshot 1 Coronin actin binding protein 1C Coronin 1C | mt |
| 1 | IPI00407069 IPI00210946 IPI00408797 IPI00330341 | 37.5 | 11 | Ensembl_locations(Chr-bp):12-63202636 60S ribosomal protein L17 Ensembl_locations(Chr-bp):18-38362032 ribosomal protein L17 | unknown |
| 1 | IPI00402996 IPI00331345 IPI00231693 IPI00399806 | 17.9 | 4 | Ensembl_locations(Chr-bp):13-107355123 Rps3a protein Ribosomal protein s3a 40S ribosomal protein s3a | unknown |
| 0.87 | IPI00272154 | 13.8 | 2 | Ensembl_locations(Chr-bp):13-22052137 | unknown |
| 1 | IPI00190013 (IPI00193870) (IPI00387755) IPI00372019 (IPI00390330) | 15.9 | 3 | Ensembl_locations(Chr-bp):13-52881955  Similar to coagulation factor XIII beta subunit | ER |
| 1 | IPI00403923 IPI00223253 IPI00199403 (IPI00194974) IPI00224575 (IPI00268549) (IPI00231295) | 29.4 | 7 | Ensembl_locations(Chr-bp):13-58182115 Splice isoform 1 of Q07244 Heterogeneous nuclear ribonucleoprotein K Ensembl_locations(Chr-bp):17-12196657  Splice isoform 2 of Q07244 Heterogeneous nuclear ribonucleoprotein K | membrane |
| 0.75 | IPI00392681 IPI00205215 | 11.8 | 2 | Ensembl_locations(Chr-bp):13-79827596 E-selectin precursor | unknown |
| 0.99 | IPI00267881 | 23.6 | 2 | Ensembl_locations(Chr-bp):14-55879381 | mt |
| 1 | IPI00121419 | 31.5 | 4 | Ensembl_locations(Chr-bp):14-8199428 | unknown |
| 1 | IPI00215093 | 43.5 | 11 | Ensembl_locations(Chr-bp):14-87049366 | unknown |
| 1 | IPI00406328 | 34.1 | 5 | Ensembl_locations(Chr-bp):1-53430187 | unknown |
| 1 | IPI00402838 IPI00230507 | 66.9 | 20 | Ensembl_locations(Chr-bp):15-5342982ATP synthase  H+ transporting mitochondrial F0 complex subunit d | mt |
| 0.98 | IPI00351827 (IPI00407453) | 5.4 | 2 | Ensembl_locations(Chr-bp):15-91249483 | mt |
| 0.73 | IPI00215102 IPI00373768 IPI00221751 | 6.8 | 2 | Ensembl_locations(Chr-bp):16-1896119 Similar to RIKEN cDNA A630054L15 Hypothetical protein | unknown |
| 1 | IPI00127684 IPI00377912 (IPI00124015) | 19 | 4 | Ensembl_locations(Chr-bp):16-19895405 Similar to presenilin associated rhomboid-like | mt |
| 1 | IPI00209436 | 24.5 | 10 | Ensembl_locations(Chr-bp):16-75638420 | unknown |
| 1 | IPI00393034 IPI00191707  IPI00391021 (IPI00337893) | 34.1 | 20 | Ensembl_locations(Chr-bp):16-87945852 Pyruvate dehydrogenase E1 component alpha subunit somatic form mitochondrial precursor  Similar to pyruvate dehydrogenase | mt |
| 0.61 | IPI00271486 | 14.5 | 2 | Ensembl_locations(Chr-bp):17-29247387 | unknown |
| 1 | IPI00409462 IPI00265517 IPI00215291 IPI00331773 | 10 | 4 | Ensembl_locations(Chr-bp):17-34902259 HLA-B-associated transcript 1A Probable ATP-dependent RNA helicase p47 Putative RNA helicase | unknown |
| 1 | IPI00210725 IPI00169804 | 31.4 | 3 | Ensembl_locations(Chr-bp):17-35052165 Hypothetical protein | mt |
| 0.95 | IPI00211262 (IPI00394614) | 14.9 | 2 | Ensembl_locations(Chr-bp):17-36308841 | unknown |
| 1 | IPI00189903 IPI00230133 | 5.9 | 2 | Ensembl_locations(Chr-bp):17-50363457 Histone 1 h1b | mt |
| 1 | IPI00405628 | 34.7 | 7 | Ensembl_locations(Chr-bp):17-81874967 | mt |
| 0.97 | IPI00388475 IPI00203637 | 7.1 | 2 | Ensembl_locations(Chr-bp):18-29193192 Anion exchange protein 4 | mt |
| 0.91 | IPI00360249 | 13.6 | 2 | Ensembl_locations(Chr-bp):18-29741881 | unknown |
| 1 | IPI00393794 (IPI00209373) IPI00117063 IPI00341822 (IPI00361397) (IPI00362587) | 3.7 | 2 | Ensembl_locations(Chr-bp):18-44371798  RNA-binding protein FUS Similar to pigpen | mt |
| 1 | IPI00394262 IPI00373214 IPI00129792 | 6.6 | 4 | Ensembl_locations(Chr-bp):1-86569163 Similar to importin alpha Q1 Importin alpha-4 subunit | mt |
| 0.89 | IPI00206222 | 21 | 3 | Ensembl_locations(Chr-bp):18-7124077 | unknown |
| 0.69 | IPI00391840 IPI00125959 (IPI00198544) IPI00406157 | 7.9 | 2 | Ensembl_locations(Chr-bp):18-73274615 Mothers against decapentaplegic homolog 2  Ensembl_locations(Chr-bp):18-76459356 | mt |
| 1 | IPI00120532 | 42.5 | 5 | Ensembl_locations(Chr-bp):19-16167152 | unknown |
| 1 | IPI00405289 IPI00214582 (IPI00136984) | 28.9 | 9 | Ensembl_locations(Chr-bp):19-23760442 40S ribosomal protein S7 | unknown |
| 1 | IPI00108886 | 17.3 | 2 | Ensembl_locations(Chr-bp):19-8228174 | mt |
| 0.93 | IPI00345544 | 17.6 | 2 | Ensembl_locations(Chr-bp):2_random_NT_080083-18621 | unknown |
| 1 | IPI00393372 IPI00361431 IPI00191142 (PI00112448) | 23.9 | 9 | Ensembl_locations(Chr-bp):20-5849586 Similar to 40S ribosomal protein S10 40S ribosomal protein S10 | mt |
| 1 | IPI00283479 | 26.3 | 5 | Ensembl_locations(Chr-bp):2-107289856 | unknown |
| 1 | IPI00211070 | 19.6 | 6 | Ensembl_locations(Chr-bp):2-28254679 | mt |
| 1 | IPI00405639 IPI00402942 IPI00395206 IPI00379121 IPI00110383 | 19.7 | 3 | Ensembl_locations(Chr-bp):2-81001104 Ensembl_locations(Chr-bp):7-108033572 Mitotic phosphoprotein 44 Similar to mitotic phosphoprotein 44 2310006I24Rik protein | mt |
| 1 | IPI00267820 IPI00227392  IPI00231677 | 27.5 | 5 | Ensembl_locations(Chr-bp):3-101641928 Tyrosine 3-monooxygenase\tryptophan 5-monooxygenase activation protein eta polypeptide Tyrosine 3\tryptophan 5 -monooxygenase activation protein eta polypeptide | unknown |
| 0.65 | IPI00387816 | 6.7 | 2 | Ensembl_locations(Chr-bp):3-103124760 | unknown |
| 0.67 | IPI00400703 IPI00363958 | 12.1 | 2 | Ensembl_locations(Chr-bp):3-105496299 Similar to AF15q14 protein isoform 2 | unknown |
| 1 | IPI00213297 | 13.7 | 3 | Ensembl_locations(Chr-bp):3-106206124 | unknown |
| 0.99 | IPI00356034 IPI00379270 IPI00192280 (IPI00379549) IPI00391753 (IPI00379359) | 13.3 | 2 | Ensembl_locations(Chr-bp):3-134323494 Similar to 60S ribosomal protein L34 60S ribosomal protein L34  Similar to ribosomal protein L34 | mt |
| 1 | IPI00195147 IPI00380398 IPI00364307 (IPI00357099) (IPI00342982) (IPI00345880) (IPI00365152) (IPI00341683) (IPI00271464) (IPI00381001) (IPI00352067) (IPI00366911) (IPI00270804)  IPI00380130 IPI00202003 IPI00406400 | 27.8 | 7 | Ensembl_locations(Chr-bp):3-143055142 Ensembl_locations(Chr-bp):14-67806873 Similar to 40S ribosomal protein S2  Repeat family 3 gene 40S ribosomal protein S2 Ensembl_locations(Chr-bp):17-24314040 | unknown |
| 1 | IPI00402859 IPI00112641 (IPI00204111) | 46 | 2 | Ensembl_locations(Chr-bp):3-148495701 15 kda selenoprotein precursor | mt |
| 0.91 | IPI00212124 (IPI00392456) | 7.1 | 2 | Ensembl_locations(Chr-bp):3-48137016 | unknown |
| 0.78 | IPI00201608 IPI00199737 IPI00128689 | 2.3 | 2 | Ensembl_locations(Chr-bp):3-6826472 Alpha 1 type V collagen Collagen a1(V) | unknown |
| 0.79 | IPI00389083 | 30.4 | 2 | Ensembl_locations(Chr-bp):3-72668730 | unknown |
| 1 | IPI00405891 IPI00407216 | 59.8 | 2 | Ensembl_locations(Chr-bp):3-86219848 Ensembl_locations(Chr-bp):X-81585578 | nuleus |
| 1 | IPI00407736 IPI00359978 IPI00137736 IPI00122674 | 17.4 | 2 | Ensembl_locations(Chr-bp):4-117606018 Similar to 40S ribosomal protein S28 40S ribosomal protein S28 Ensembl_locations(Chr-bp):Un_random_NT_084270-1060 | unknown |
| 1 | IPI00191073 IPI00128904 | 21.9 | 4 | Ensembl_locations(Chr-bp):4-120963919 Poly(rc)-binding protein 1 | mt |
| 1 | IPI00388020 IPI00222546 | 39.1 | 5 | Ensembl_locations(Chr-bp):4-121301801 ribosomal protein L22 | mt |
| 1 | IPI00277112 IPI00131407 | 22 | 4 | Ensembl_locations(Chr-bp):4-134495288 Proteasome subunit alpha type 5 | unknown |
| 1 | IPI00405994 IPI00369264 IPI00331461 IPI00359103 | 25.6 | 5 | Ensembl_locations(Chr-bp):4-134829368 Splice isoform 2 of P39026 RIKEN cDNA 2010203J19 Similar to 60S ribosomal protein L11 | unknown |
| 1 | IPI00213436 IPI00125929 | 26.8 | 4 | Ensembl_locations(Chr-bp):4-37244834 NADH-ubiquinone oxidoreductase MLRQ subunit | mt |
| 0.83 | IPI00391030 | 4.3 | 2 | Ensembl_locations(Chr-bp):4-38745259 | unknown |
| 1 | IPI00392756 IPI00212767 | 8.1 | 2 | Ensembl_locations(Chr-bp):4-69289249 Trypsin I anionic precursor | mt |
| 1 | IPI00212969 IPI00358211 IPI00405058 IPI00315227 | 19.9 | 5 | Ensembl_locations(Chr-bp):4-79976457 Similar to heterogeneous nuclear ribonucleoprotein A2\B1 Heterogeneous nuclear ribonucleoprotein A2\B1 isoform 2 Heterogeneous nuclear ribonucleoproteins A2\B1 | unknown |
| 0.99 | IPI00209456 | 8.2 | 3 | Ensembl_locations(Chr-bp):4-83684451 | unknown |
| 1 | IPI00213751 IPI00192134 | 15.2 | 3 | Ensembl_locations(Chr-bp):5-136282552 Similar to RIKEN cDNA 2810405F18 | ER |
| 0.7 | IPI00215601 | 11.4 | 2 | Ensembl_locations(Chr-bp):5-22701950 | unknown |
| 1 | IPI00400595 | 26 | 18 | Ensembl_locations(Chr-bp):5-59472634 | mt |
| 1 | IPI00403619 IPI00208205 (IPI00323357) | 49.2 | 4 | Ensembl_locations(Chr-bp):5-5967967 Heat shock cognate 71 kda protein | mt |
| 1 | IPI00392655 | 23.9 | 4 | Ensembl_locations(Chr-bp):5-76960675 | unknown |
| 1 | IPI00225480 IPI00377793 IPI00405507 | 20.3 | 5 | Ensembl_locations(Chr-bp):6-130258692 Ribosomal protein L18 60S ribosomal protein L18 | unknown |
| 1 | IPI00134129 | 27.3 | 2 | Ensembl_locations(Chr-bp):6-41455019 | mt |
| 0.84 | IPI00409173 IPI00222188 | 10.8 | 2 | Ensembl_locations(Chr-bp):6-4211505 Procollagen type I alpha 2 | mt |
| 0.96 | IPI00198885 | 4.1 | 2 | Ensembl_locations(Chr-bp):6-76040238 | mt |
| 0.81 | IPI00123946 | 19.2 | 2 | Ensembl_locations(Chr-bp):7-10498390 | unknown |
| 1 | IPI00390784 | 19.3 | 5 | Ensembl_locations(Chr-bp):7-119763736 | unknown |
| 1 | IPI00389989 | 42.7 | 6 | Ensembl_locations(Chr-bp):7-120258007 | unknown |
| 1 | IPI00112342 | 46 | 9 | Ensembl_locations(Chr-bp):7-133516038 | unknown |
| 1 | IPI00408520 IPI00229548 | 14.8 | 3 | Ensembl_locations(Chr-bp):7-13826504 Neutral amino acid transporter ASCT2 | unknown |
| 1 | IPI00392935 IPI00365944 IPI00354819 IPI00409817 | 51.1 | 14 | Ensembl_locations(Chr-bp):7-1772774 Similar to 17 00 dalton myosin light chain Myosin light chain alkali nonmuscle Splice isoform Non-muscle of Q60605 Myosin light polypeptide 6 | mt |
| 0.93 | IPI00402850 IPI00230992 (IPI00317902) | 14.4 | 2 | Ensembl_locations(Chr-bp):7-3221644 Proteasome (prosome macropain) subunit beta type 5 | unknown |
| 1 | IPI00388922 IPI00169537 IPI00204561 | 21.9 | 3 | Ensembl_locations(Chr-bp):7-74672459 Mitochondrial folate transporter\carrier Similar to mitochondrial folate transporter\carrier | mt |
| 0.91 | IPI00111771 | 7.4 | 2 | Ensembl_locations(Chr-bp):7-89454388 | unknown |
| 1 | IPI00393068 IPI00391407 IPI00366256 IPI00171981 | 4.7 | 2 | Ensembl_locations(Chr-bp):7-94609663 Ensembl_locations(Chr-bp):Un-65469655 Similar to RIKEN cDNA 1110021N07 1110021N07Rik protein | unknown |
| 0.92 | IPI00390624 | 33 | 2 | Ensembl_locations(Chr-bp):7-97720206 | unknown |
| 0.93 | IPI00387790 | 6.5 | 2 | Ensembl_locations(Chr-bp):7-9919331 | mt |
| 1 | IPI00351010 | 20.7 | 9 | Ensembl_locations(Chr-bp):8-106146795 | unknown |
| 1 | IPI00406634 IPI00170128 IPI00327108 | 13.4 | 5 | Ensembl_locations(Chr-bp):8-124923273 Paraplegin Paraplegin | mt |
| 0.76 | IPI00204851 (IPI00393140) (IPI00393148) | 6.8 | 2 | Ensembl_locations(Chr-bp):8-127613226 | unknown |
| 0.99 | IPI00203823 IPI00368955  IPI00197711 | 9.7 | 2 | Ensembl_locations(Chr-bp):8-31694432 Similar to L-lactate dehydrogenase A chain (LDH-A) (LDH muscle subunit) (LDH-M) L-lactate dehydrogenase A chain | mt |
| 1 | IPI00392438 | 51.2 | 2 | Ensembl_locations(Chr-bp):8-43793405 | unknown |
| 1 | IPI00264580 | 36.7 | 4 | Ensembl_locations(Chr-bp):8-45774790 | unknown |
| 1 | IPI00404424 | 3.6 | 4 | Ensembl_locations(Chr-bp):8-81422165 | unknown |
| 0.66 | IPI00116577 | 11 | 2 | Ensembl_locations(Chr-bp):9-22790028 | unknown |
| 0.65 | IPI00388838 | 11.9 | 2 | Ensembl_locations(Chr-bp):9-63498984 | unknown |
| 0.94 | IPI00393349 IPI00230359 IPI00367110 IPI00404267 | 6.2 | 2 | Ensembl_locations(Chr-bp):9-82346141 HIV-1 Rev binding protein Similar to HIV-1 Rev binding protein Nucleoporin-like protein Rab homolog | unknown |
| 0.96 | IPI00265757 | 7.7 | 2 | Ensembl_locations(Chr-bp):9-92557406 | unknown |
| 0.74 | IPI00407875 | 19.9 | 2 | Ensembl_locations(Chr-bp):Un_random_NT_052104-340 | unknown |
| 1 | IPI00407792 IPI00381069 IPI00122426 (IPI00202214) | 11.1 | 4 | Ensembl_locations(Chr-bp):Un_random_NT_055704-347 Similar to 60S ribosomal protein L19 60S ribosomal protein L19 | mt |
| 0.9 | IPI00406403 IPI00381837 | 4.6 | 2 | Ensembl_locations(Chr-bp):Un_random_NT_060620-5611 Similar to putative pheromone receptor | unknown |
| 1 | IPI00408501 IPI00319973 | 44.1 | 10 | Ensembl_locations(Chr-bp):X-27924719 Progesterone receptor membrane component | mt |
| 0.61 | IPI00391069 | 10 | 3 | Ensembl_locations(Chr-bp):X-44263086 | unknown |
| 1 | IPI00390048 IPI00393237 | 32.4 | 7 | Ensembl_locations(Chr-bp):X-81879111 Similar to RIKEN cDNA 0610008C08 | unknown |
| 1 | IPI00406960 | 18.7 | 12 | Env polyprotein | mt |
| 1 | IPI00212478 IPI00388857 (IPI00393327) | 15.8 | 3 | Epsilon 1 globin Ensembl_locations(Chr-bp):1-161659551 | unknown |
| 1 | IPI00387407 | 25.2 | 8 | Ero1l | ER |
| 1 | IPI00187240  IPI00364587 | 4 | 2 | ES cells cDNA RIKEN full-length enriched library clone:2400010F03 product:DEAD box protein homolog Similar to DEAD (Asp-Glu-Ala-Asp) box polypeptide 47 | unknown |
| 0.99 | IPI00344004 | 24.8 | 4 | ES cells cDNA RIKEN full-length enriched library clone:2410011G03 product:NADH-ubiquinone oxidoreductase subunit B17.2 (EC 1.6.5.3) (EC 1.6.99.3) (Complex I-B17.2) (CI-B17.2) (CIB17.2) homolog | unknown |
| 1 | IPI00316067 | 20.2 | 5 | Estradiol 17 beta-dehydrogenase 7 | mt |
| 1 | IPI00320208 IPI00330981 | 26.8 | 3 | Eukaryotic translation elongation factor 1 beta 2 Elongation factor 1-beta | unknown |
| 1 | IPI00203214 IPI00337957 | 29.5 | 26 | Eukaryotic translation elongation factor 2 Elongation factor 2 | mt |
| 1 | IPI00230830 (IPI00317216) | 23.5 | 2 | Eukaryotic translation initiation factor 2 subunit 1 alpha | mt |
| 1 | IPI00120914 IPI00371204 | 13 | 2 | Eukaryotic translation initiation factor 3 subunit 5 Similar to eukaryotic translation initiation factor 3 subunit 5 epsilon 47kda | mt |
| 1 | IPI00115751 IPI00366292 | 8.4 | 3 | Eukaryotic translation initiation factor 3 subunit 7 Similar to eukaryotic translation initiation factor 3 subunit 7 | mt |
| 1 | IPI00369618 IPI00118676 | 48.3 | 28 | Eukaryotic translation initiation factor 4A isoform 1 Eukaryotic initiation factor 4A-I | unknown |
| 1 | IPI00115862 | 27.3 | 3 | Eukaryotic translation initiation factor 6 | mt |
| 1 | IPI00395038 IPI00287338 | 9.7 | 6 | Exportin 1 CRM1 homolog Exportin 1 (CRM1 yeast homolog) | nuleus,cytoplasm |
| 1 | IPI00264211 | 7.8 | 4 | Exportin 5 | nucleus, cytoplasm |
| 0.91 | IPI00354924 | 3.2 | 2 | Expressed sequence AI931714 | unknown |
| 0.96 | IPI00330128 | 3.3 | 3 | Expressed sequence AW456499 | unknown |
| 1 | IPI00229601 | 4.4 | 3 | Fad104 | mt |
| 1 | IPI00404474 (IPI00117176) | 6.9 | 3 | Fatty acid amide hydrolase | mt |
| 1 | IPI00387535 IPI00169772 | 21.1 | 9 | Fatty acid coenzyme A ligase long chain 3 Long-chain-fatty-acid--coa ligase 3 | mt |
| 1 | IPI00285029 | 13.9 | 22 | Fatty acid synthase | mt |
| 1 | IPI00327830 | 28.9 | 2 | Fatty acid-binding protein epidermal | mt |
| 1 | IPI00111235 | 22.1 | 8 | Fatty aldehyde dehydrogenase | mt |
| 0.89 | IPI00348513 | 6.1 | 2 | F-box only protein 13 | unknown |
| 0.81 | IPI00112966 IPI00366732 IPI00392689 | 6.8 | 2 | F-box\LRR-repeat protein 2 Similar to F-box and leucine-rich repeat protein 2 Ensembl_locations(Chr-bp):8-118640969 | unknown |
| 1 | IPI00315299 IPI00406515 | 23.5 | 2 | Ferritin light chain 1 Ensembl_locations(Chr-bp):13-71959052 | mt |
| 1 | IPI00228343 | 31.5 | 11 | Ferrochelatase | mt |
| 0.84 | IPI00204009 | 6.1 | 2 | Fibrillin-2 | unknown |
| 0.74 | IPI00331030 (IPI00112944) | 16.4 | 3 | Fidgetin-like 1 | unknown |
| 1 | IPI00133931 | 22.4 | 3 | FK506 binding protein 11 precursor | unknown |
| 1 | IPI00126000 | 30.4 | 5 | FK506-binding protein 3 | mt |
| 1 | IPI00114365 | 11.6 | 4 | Folylpolyglutamate synthase mitochondrial precursor | mt |
| 1 | IPI00129928 | 54.8 | 41 | Fumarate hydratase mitochondrial precursor | mt |
| 1 | IPI00380446 | 28 | 6 | Gag protein | mt |
| 1 | IPI00108206 | 3.8 | 3 | Gag-Pol polyprotein | mt |
| 1 | IPI00136929 | 52.4 | 26 | Gamma actin-like protein | mt |
| 0.79 | IPI00192642 (IPI00113772) | 20.9 | 4 | Gamma-aminobutyric-acid receptor alpha-1 subunit precursor | mt |
| 1 | IPI00123756 | 33.2 | 5 | Gangioside-induced differentiation-associated protein 1-like 1 | mt |
| 0.84 | IPI00113992 | 5 | 2 | GDP-mannose pyrophosphorylase B homolog | unknown |
| 1 | IPI00230715 | 33.3 | 7 | Genes associated with retinoid-IFN-induced mortality 19 | mt |
| 1 | IPI00123306 | 18.9 | 6 | Germ cell-specific ankyrin SAM and basic leucine zipper domain containing protein | cytoplasm |
| 1 | IPI00211776 | 5.1 | 6 | Giantin | Golgi |
| 0.62 | IPI00132801 | 8.6 | 2 | Glucagon-like peptide 1 receptor precursor | mt |
| 1 | IPI00228633 | 11.1 | 3 | Glucose phosphate isomerase 1 | mt |
| 1 | IPI00228385 | 17.9 | 6 | Glucose-6-phosphate dehydrogenase X-linked | unknown |
| 1 | IPI00330323 (IPI00131467) | 11.4 | 5 | Glucosidase 1 | mt |
| 1 | IPI00191733 (IPI00318522) | 6.5 | 2 | Glutamate decarboxylase 65 kda isoform | mt |
| 1 | IPI00114209 | 59.3 | 63 | Glutamate dehydrogenase mitochondrial precursor | mt |
| 1 | IPI00340523 | 44.1 | 28 | Glutaminase | mt |
| 1 | IPI00339916 | 12.6 | 12 | Glutamyl-prolyl-tRNA synthetase | unknown |
| 1 | IPI00331282 IPI00190531 IPI00111359 | 12.6 | 3 | Glutathione reductase 1 Glutathione reductase Glutathione reductase mitochondrial precursor | mt |
| 1 | IPI00387307 | 42.2 | 30 | Glycerol phosphate dehydrogenase 1 mitochondrial | mt |
| 1 | IPI00127267 | 18.3 | 13 | Glycine dehydrogenase [decarboxylating] mitochondrial precursor | mt |
| 1 | IPI00322631 | 7.5 | 5 | Glycosylphosphatidylinositol anchor attachment 1 protein | mt |
| 0.62 | IPI00121566 | 14.8 | 2 | GMP reductase 1 | unknown |
| 1 | IPI00122399 | 13.1 | 8 | Golgi apparatus protein 1 precursor | mt |
| 1 | IPI00316682 | 6.2 | 2 | Golgi autoantigen golgin subfamily A member 5 | mt |
| 1 | IPI00319488 | 10.5 | 17 | Golgi autoantigen golgin subfamily b macrogolgin 1 | mt |
| 0.71 | IPI00134035 IPI00349520 | 3.9 | 3 | Gp330 Low density lipoprotein receptor-related protein 2 LRP2 | unknown |
| 1 | IPI00226790 | 19.9 | 9 | GPI transamidase component PIG-T precursor | mt |
| 1 | IPI00121515 | 7.4 | 2 | GPI-anchored membrane protein 1 | mt |
| 0.84 | IPI00396730 | 14.9 | 2 | Growth differentiation factor 10 | mt |
| 1 | IPI00205598 | 49.3 | 13 | Grpe protein homolog 1 mitochondrial precursor | unknown |
| 1 | IPI00130159 IPI00269571 | 9.9 | 2 | Grpe protein homolog 2 mitochondrial precursor Ensembl_locations(Chr-bp):7-27322470 | mt |
| 1 | IPI00225963 | 40.1 | 23 | GRSF-1 protein homolog | unknown |
| 1 | IPI00404970 (IPI00224070) | 13.2 | 2 | Gtpase-activating protein rhogap homolog | mt |
| 1 | IPI00212796 (PI00134621) | 30.6 | 9 | GTP-binding nuclear protein RAN | mt |
| 1 | IPI00231925 IPI00228617 | 19.4 | 4 | GTP-binding protein (G-alpha-i2) Guanine nucleotide binding protein alpha inhibiting 2 | unknown |
| 1 | IPI00213685 IPI00116688 | 24.2 | 2 | GTP-binding protein Rab-3D Ras-related protein Rab-3D | cytoplasm |
| 0.83 | IPI00118021 | 14.9 | 2 | Gtrgeo22 | unknown |
| 1 | IPI00338854 (IPI00231726) | 22 | 4 | Guanine nucleotide binding protein alpha inhibiting 3 | unknown |
| 1 | IPI00121387 (IPI00200437) | 13.4 | 3 | Guanine nucleotide-binding protein alpha-11 subunit | mt |
| 1 | IPI00118569 | 14.1 | 4 | Guanine nucleotide-binding protein alpha-13 subunit | mt |
| 1 | IPI00317740 (IPI00231134) | 5 | 2 | Guanine nucleotide-binding protein beta subunit-like protein 12.3 | mt |
| 1 | IPI00120716 (IPI00212655) | 12.6 | 2 | Guanine nucleotide-binding protein G(I)\G(S)\G(T) beta subunit 1 |  |
| 1 | IPI00162780 IPI00194489 | 28.5 | 8 | Guanine nucleotide-binding protein G(I)\G(S)\G(T) beta subunit 2 Guanine nucleotide-binding protein beta 2 | membrane |
| 1 | IPI00115546 IPI00230192 | 16.7 | 3 | Guanine nucleotide-binding protein G(o) alpha subunit 2 Guanine nucleotide binding protein alpha o | mt |
| 1 | IPI00214454 | 29.6 | 4 | H protein | unknown |
| 1 | IPI00128655 IPI00135946 | 63.8 | 24 | Haymaker protein Probable mitochondrial import receptor subunit TOM40 homolog | mt |
| 1 | IPI00387868 IPI00331556 | 7.6 | 5 | Heat shock 70 kda protein 4 Heat shock protein 110 kda | mt |
| 1 | IPI00229080 IPI00191466 | 46.3 | 26 | Heat shock protein 1 beta Similar to heat shock protein 84 - mouse | mt |
| 0.99 | IPI00123802 IPI00224109 | 3.8 | 2 | Heat shock protein 105 Splice isoform HSP105-beta of Q61699 Heat-shock protein 105 kda | mt |
| 1 | IPI00132762 | 45.3 | 26 | Heat shock protein 75 kda mitochondrial precursor | mt |
| 0.61 | IPI00309516 | 3.1 | 2 | Hedgehog-interacting protein | unknown |
| 1 | IPI00270064 IPI00203041 IPI00127902 | 16.3 | 11 | HELG Similar to 2810477H02Rik protein Hypothetical protein | mt |
| 1 | IPI00131577 | 28.7 | 6 | Heme oxygenase 1 | mt |
| 1 | IPI00309322 (IPI00400219) | 33.7 | 9 | Heme oxygenase 2 | microsome |
| 1 | IPI00135085 | 28.4 | 5 | Heme-binding protein | mt |
| 1 | IPI00110658 IPI00313201 | 16.9 | 3 | Hemoglobin alpha adult chain 1 13 days embryo liver cDNA RIKEN full-length enriched library clone:2500002C06 full insert sequence | unknown |
| 1 | IPI00117288 IPI00212724 IPI00277066 IPI00208193 IPI00212723 | 21.4 | 5 | Heterogeneous nuclear ribonucleoprotein A\B Type A\B hnrnp p40 Hnrpab protein Nucleic acid binding factor prm10 Type A\B hnrnp P38 | mt |
| 1 | IPI00205916 IPI00223095 | 11.5 | 2 | Hexaprenyldihydroxybenzoate methyltransferase mitochondrial precursor Hexaprenyldihydroxybenzoate methyltransferase | mt |
| 1 | IPI00114342 (IPI00201057) | 14 | 6 | Hexokinase type II | mt |
| 1 | IPI00278230 | 41.2 | 5 | HIRA-interacting protein 5 | mt |
| 1 | IPI00223713 IPI00231650 | 10.4 | 3 | Histone 1 h1c H1 histone family member 4 | unknown |
| 1 | IPI00378190 | 58.8 | 6 | Histone H2A.F\Z variant | mt |
| 0.83 | IPI00115645 | 3.6 | 3 | Histone H3 methyltransferase DOT1 | unknown |
| 1 | IPI00231340 IPI00336741 IPI00407339 IPI00329998 (PI00366335)  (PI00368661) | 50 | 10 | Histone H4 Hist1h4i protein germinal histone H4 Similar to germinal histone H4 gene | unknown |
| 1 | IPI00191621 | 28.7 | 3 | Hormone-regulated proliferation-associated 20 kda protein | unknown |
| 0.67 | IPI00222264 | 10.1 | 3 | HRIHFB2003 protein homolog | unknown |
| 1 | IPI00129964 (IPI00382352) IPI00322435 | 13.3 | 3 | HS1-binding protein  HAX1XS | mt |
| 1 | IPI00116308 | 7.5 | 2 | Hsc70-interacting protein | mt |
| 1 | IPI00275907 | 9.2 | 6 | HSGCN1 homolog | unknown |
| 1 | IPI00330804 IPI00208256 | 28.5 | 11 | Hspca protein Heat shock protein 86 | cytoplasm |
| 0.86 | IPI00371091 | 3.2 | 2 | Huntingtin interacting protein 1 related | unknown |
| 0.99 | IPI00319167 | 2.5 | 5 | Hydrocephalus inducing | mt |
| 1 | IPI00320847 | 47 | 12 | Hydroxyacyl-Coenzyme A dehydrogenase type II | unknown |
| 1 | IPI00231253 | 83.5 | 29 | Hydroxysteroid (17-beta) dehydrogenase 10 | unknown |
| 1 | IPI00408421 | 20.1 | 11 | Hydroxysteroid 17-beta dehydrogenase 4 | mt |
| 1 | IPI00312759 | 14.9 | 4 | Hypothetical aminoacyl-transfer RNA synthetases class-II containing protein | mt |
| 1 | IPI00226932 | 32.2 | 32 | Hypothetical ARM repeat structure containing protein | mt |
| 0.95 | IPI00227548 | 3.3 | 2 | Hypothetical copper amine oxidase containing protein | membrane |
| 1 | IPI00312811  IPI00204917 | 16.9 | 2 | Hypothetical D111\G-patch domain\zinc finger C-x8-C-x5-C-x3-H type containing protein ARF-related protein | mt |
| 1 | IPI00226466 | 43.6 | 27 | Hypothetical DNAj N-terminal domain containing protein | mt |
| 0.95 | IPI00379123 | 5.6 | 2 | Hypothetical dynein heavy chain containing protein | unknown |
| 0.89 | IPI00407367  IPI00330355 | 8.5 | 2 | Hypothetical E1-E2 atpases\haloacid dehalogenase\epoxide hydrolase family containing protein RIKEN cDNA 9330174J19 | unknown |
| 1 | IPI00226726 | 18.2 | 5 | Hypothetical FAD\NAD | unknown |
| 1 | IPI00409837 IPI00115493 | 30 | 8 | Hypothetical glutamyl-tRNA Similar to PET112 | mt |
| 1 | IPI00117083 | 55.8 | 14 | Hypothetical grpe protein homolog | unknown |
| 1 | IPI00314166  (IPI00403586) | 24.8 | 5 | Hypothetical Lipolytic enzymes\esterase\lipase\thioesterase family active site containing protein | mt |
| 1 | IPI00312018 IPI00409116 | 27.5 | 6 | Hypothetical Microbodies C-terminal targeting signal containing protein Ensembl_locations(Chr-bp):5-114224299 | mt |
| 1 | IPI00221569 | 16.2 | 4 | Hypothetical Microbodies C-terminal targeting signal\zinc-containing alcohol dehydrogenase superfamily containing protein | mt |
| 0.99 | IPI00227451 | 15.2 | 2 | Hypothetical phosphoglycerate mutase family containing protein | unknown |
| 0.96 | IPI00273052  IPI00109743 IPI00400159 | 5.2 | 2 | Hypothetical P-loop containing nucleotide triphosphate hydrolases structure containing protein 4122402O22Rik protein RIKEN cDNA 4122402O22 | mt |
| 1 | IPI00230351 | 60.2 | 47 | Hypothetical protein | unknown |
| 1 | IPI00137368 | 27.2 | 46 | Hypothetical protein | unknown |
| 1 | IPI00123129 | 42.1 | 37 | Hypothetical protein | unknown |
| 1 | IPI00130288 | 43.2 | 31 | Hypothetical protein | unknown |
| 1 | IPI00153660 | 41.7 | 29 | Hypothetical protein | unknown |
| 1 | IPI00276926 | 37.3 | 24 | Hypothetical protein | mt |
| 1 | IPI00115977 | 47.2 | 22 | Hypothetical protein | unknown |
| 1 | IPI00323177 | 38.4 | 20 | Hypothetical protein | unknown |
| 1 | IPI00380316 | 40.1 | 17 | Hypothetical protein | unknown |
| 1 | IPI00128281 | 45.4 | 15 | Hypothetical protein | unknown |
| 1 | IPI00114878 | 43.7 | 14 | Hypothetical protein | unknown |
| 1 | IPI00331590 | 59.2 | 10 | Hypothetical protein | unknown |
| 1 | IPI00267299 | 17.4 | 9 | Hypothetical protein | unknown |
| 1 | IPI00221850 | 36.1 | 9 | Hypothetical protein | mt |
| 1 | IPI00111211 | 25.4 | 9 | Hypothetical protein | mt |
| 1 | IPI00127227 | 26.3 | 7 | Hypothetical protein | unknown |
| 1 | IPI00153103 | 26.9 | 6 | Hypothetical protein | Nucleus |
| 1 | IPI00170339 | 14.8 | 5 | Hypothetical protein | unknown |
| 1 | IPI00336437 | 26.7 | 5 | Hypothetical protein | unknown |
| 1 | IPI00169870 | 7.9 | 5 | Hypothetical protein | mt |
| 1 | IPI00226901 | 23.6 | 5 | Hypothetical protein | cytoplasm |
| 1 | IPI00229722 | 11.2 | 5 | Hypothetical protein | unknown |
| 1 | IPI00230353 | 18.8 | 4 | Hypothetical protein | unknown |
| 1 | IPI00115627 | 14.8 | 4 | Hypothetical protein | unknown |
| 1 | IPI00309972 | 18.9 | 4 | Hypothetical protein | unknown |
| 1 | IPI00353563 | 5.7 | 4 | Hypothetical protein | unknown |
| 1 | IPI00116138 | 15.8 | 4 | Hypothetical protein | mt |
| 0.8 | IPI00338302 | 10.5 | 4 | Hypothetical protein | unknown |
| 1 | IPI00380290 | 5.9 | 3 | Hypothetical protein | unknown |
| 1 | IPI00153101 | 5.3 | 3 | Hypothetical protein | mt |
| 1 | IPI00170373 | 10.9 | 3 | Hypothetical protein | unknown |
| 1 | IPI00228106 | 20.4 | 3 | Hypothetical protein | unknown |
| 1 | IPI00331533 | 20.6 | 3 | Hypothetical protein | mt |
| 0.7 | IPI00228917 | 6.2 | 3 | Hypothetical protein | unknown |
| 0.98 | IPI00404108 | 30.5 | 2 | Hypothetical protein | mt |
| 0.94 | IPI00405128 | 25 | 2 | Hypothetical protein | mt |
| 0.69 | IPI00339467 | 11.3 | 2 | Hypothetical protein | unknown |
| 0.61 | IPI00221455 | 25.5 | 2 | Hypothetical protein | unknown |
| 0.6 | IPI00130763 | 9.2 | 2 | Hypothetical protein | unknown |
| 0.9 | IPI00169793 | 16.3 | 2 | Hypothetical protein | unknown |
| 0.97 | IPI00229120 | 10.3 | 2 | Hypothetical protein | mt |
| 1 | IPI00122032 | 11.1 | 2 | Hypothetical protein | unknown |
| 1 | IPI00123765 | 11.3 | 2 | Hypothetical protein | unknown |
| 1 | IPI00266836 | 19.9 | 2 | Hypothetical protein | unknown |
| 1 | IPI00402903 | 12.9 | 2 | Hypothetical protein | unknown |
| 1 | IPI00124082 | 22.8 | 2 | Hypothetical protein | mt |
| 0.68 | IPI00121528 | 15.8 | 2 | Hypothetical protein | unknown |
| 0.66 | IPI00380424 | 4 | 2 | Hypothetical protein | unknown |
| 1 | IPI00381357 | 14 | 2 | Hypothetical protein | mt |
| 0.93 | IPI00170090 | 7.2 | 2 | Hypothetical protein | unknown |
| 0.82 | IPI00223200 | 22.6 | 2 | Hypothetical protein | unknown |
| 0.96 | IPI00229288 | 5.5 | 3 | Hypothetical protein 8030448M07 | unknown |
| 1 | IPI00139378 | 58.8 | 2 | Hypothetical protein BM-002 | mt |
| 1 | IPI00122075 | 13.5 | 4 | Hypothetical protein MGC25836 | unknown |
| 0.75 | IPI00121525 | 15.7 | 3 | Hypothetical protein MGC37914 | unknown |
| 1 | IPI00341671 | 14.4 | 3 | Hypothetical protein XP_203663 | mt |
| 0.61 | IPI00193126 | 10.8 | 2 | Hypothetical protein XP_233002 | unknown |
| 0.98 | IPI00351189 | 39.8 | 2 | Hypothetical protein XP_287369 | unknown |
| 1 | IPI00372908 | 34.3 | 4 | Hypothetical protein XP_342191 | mt |
| 1 | IPI00196668 | 22.2 | 3 | Hypothetical protein XP_346621 | cytoplasm |
| 0.83 | IPI00377587 | 12.6 | 2 | Hypothetical protein XP_358485 | unknown |
| 1 | IPI00316469 IPI00387408 IPI00404285 IPI00382320 | 6.6 | 4 | Hypothetical protein 1300006C19Rik protein Ensembl_locations(Chr-bp):9-117615458 Ac1573 | unknown |
| 1 | IPI00267295 IPI00212512 | 10.1 | 3 | Hypothetical protein 26S proteasome non-atpase regulatory subunit 1 | unknown |
| 1 | IPI00404801 IPI00126248 (PI00214665)IPI00214801 | 16.6 | 10 | Hypothetical protein ATP-citrate synthase   Ensembl_locations(Chr-bp):10-89435308 | mt |
| 1 | IPI00380285 IPI00121575 IPI00331431 | 33.5 | 11 | Hypothetical protein Brain cDNA clone mncb-5081 1300013N08Rik protein | mt |
| 1 | IPI00124610 IPI00191587 IPI00213205 IPI00133218 | 17.2 | 3 | Hypothetical protein Ensembl_locations(Chr-bp):4-144544390 Ensembl_locations(Chr-bp):13-48122443 2610313E07Rik protein | unknown |
| 1 | IPI00226414 (IPI00404935) | 22.3 | 3 | Hypothetical protein | mt |
| 1 | IPI00169925 IPI00109167 | 40.7 | 11 | Hypothetical protein NADH-ubiquinone oxidoreductase 24 kda subunit mitochondrial precursor | mt |
| 1 | IPI00278804 IPI00194179 | 15.1 | 2 | Hypothetical protein Secretory carrier-associated membrane protein 1 | mt |
| 1 | IPI00169845 IPI00215410 | 26.7 | 7 | Hypothetical protein Similar to BC032271 protein | unknown |
| 1 | IPI00331554 IPI00208091 IPI00119581 (IPI00330634) | 4.5 | 2 | Hypothetical protein Similar to fibrillarin Fibrillarin | mt |
| 1 | IPI00109253 IPI00215064 | 36.5 | 7 | Hypothetical protein Similar to hypothetical protein | mt |
| 1 | IPI00116850 IPI00209916 | 22.8 | 5 | Hypothetical protein Similar to hypothetical protein D10Wsu52e | unknown |
| 1 | IPI00404621 IPI00359049 (IPI00407584) | 4.4 | 2 | Hypothetical protein Similar to KIAA1007 protein | mt |
| 0.89 | IPI00123494 IPI00370456 | 10.1 | 2 | Hypothetical protein Similar to proteasome 26S non-atpase subunit 2 | unknown |
| 1 | IPI00387492 IPI00169463 | 62.9 | 2 | Hypothetical protein Tubulin beta-2 chain | mt |
| 1 | IPI00222509 | 6.5 | 2 | Hypothetical regulator of chromosome condensation | ER |
| 1 | IPI00338628 IPI00339012 | 47.8 | 21 | Hypothetical ribosomal protein L1 60S ribosomal protein l10a | unknown |
| 1 | IPI00308263 IPI00282141 | 14.5 | 3 | Hypothetical RING finger containing protein 0610009K11Rik protein | mt |
| 0.96 | IPI00221464 IPI00153987 (IPI00228452) IPI00214804 | 17 | 2 | Hypothetical RING finger containing protein Hypothetical protein  Ensembl_locations(Chr-bp):8-48853342 | unknown |
| 1 | IPI00228006 IPI00110425 | 6.6 | 2 | Hypothetical RNI-like structure containing protein 2310004L02Rik protein | mt |
| 1 | IPI00138163 | 33 | 10 | Hypothetical thioredoxin containing protein | mt |
| 0.99 | IPI00356666 | 12.2 | 2 | Hypothetical von Willebrand factor type A domain containing protein | mt |
| 1 | IPI00226875 | 5.9 | 4 | Hypothetical zinc finger | unknown |
| 1 | IPI00135068 | 36.3 | 36 | Import inner membrane translocase subunit TIM44 mitochondrial precursor | mt |
| 1 | IPI00205394 | 30 | 34 | Import inner membrane translocase subunit TIM44 mitochondrial precursor | mt |
| 1 | IPI00331444 IPI00206234 | 6.6 | 3 | Importin 7 Similar to importin 7 | mt |
| 1 | IPI00323881 (IPI00204261) | 8.6 | 5 | Importin beta-1 subunit | mt |
| 1 | IPI00112414 | 17.3 | 8 | Importin-alpha re-exporter | mt |
| 1 | IPI00323971 | 7.4 | 2 | Inosine-5'-monophosphate dehydrogenase 2 | mt |
| 1 | IPI00231659 IPI00133615 | 32 | 9 | Integral membrane protein Tmp21-I (p23) precursor 1110014C03Rik protein | membrane |
| 1 | IPI00205166 IPI00330956 (IPI00331413) IPI00189857 | 6.4 | 3 | Integrin alpha 6 Similar to integrin alpha 6  Integrin alpha 6 subchain | mt |
| 1 | IPI00132474 | 28.4 | 16 | Integrin beta-1 precursor | mt |
| 0.99 | IPI00120264 | 9 | 2 | Interferon inducible protein 1 | mt |
| 1 | IPI00318550 | 31.1 | 6 | Interleukin enhancer binding factor 2 | mt |
| 0.71 | IPI00205182 IPI00206288 | 5.6 | 2 | Interleukin-6 receptor alpha chain precursor interleukin 6 receptor | mt |
| 1 | IPI00194047 (IPI00109169) | 35.9 | 24 | Isocitrate dehydrogenase [NAD] subunit gamma mitochondrial precursor | mt |
| 1 | IPI00318614 | 45.3 | 36 | Isocitrate dehydrogenase 2 (NADP+) mitochondrial | mt |
| 1 | IPI00225201 | 8.5 | 5 | Isoleucine-tRNA synthetase | unknown |
| 1 | IPI00119433 IPI00380611 | 25.5 | 9 | Isovaleryl dehydrogenase precursor 1300016K07Rik protein | unknown |
| 0.69 | IPI00136067 IPI00365920 | 3.9 | 2 | Jagged 2 precursor Jagged 2 | mt |
| 0.99 | IPI00382153 IPI00387298 IPI00139301 | 9 | 2 | Keratin 5 Hypothetical protein Type II keratin 5 | mt |
| 1 | IPI00157501 | 9.6 | 7 | Keratin complex 2 basic gene 17 | unknown |
| 1 | IPI00393340 | 38.5 | 2 | Keratin K6 |  |
| 1 | IPI00226062 IPI00110670 | 11.1 | 2 | Keratin Keratin type I cytoskeletal 10 | cytoplasm |
| 1 | IPI00392713 | 32.1 | 5 | Kidney-type glutaminase GAC isoform | unknown |
| 0.63 | IPI00320497 | 2.6 | 2 | Kinase suppressor of ras | mt |
| 1 | IPI00122559 | 26.5 | 30 | Kinectin | mt |
| 0.72 | IPI00201125 IPI00263123 IPI00407144 | 5.1 | 4 | Kinesin family member 1B Splice isoform 1 of Q60575 Kinesin-like protein KIF1B Splice isoform 2 of Q60575 Kinesin-like protein KIF1B | unknown |
| 0.99 | IPI00407864 IPI00330356 | 10.7 | 2 | Kinesin family member 23 13 days embryo head cDNA RIKEN full-length enriched library clone:3110001D19 product:kinesin-like 5 | mt |
| 0.97 | IPI00116913 | 2.5 | 3 | Laminin alpha-5 chain precursor | unknown |
| 1 | IPI00204778 (IPI00129395) IPI00331577 | 4.9 | 3 | Large neutral amino acids transporter small subunit 1  Solute carrier family 7 (Cationic amino acid transporter, y+ system) member 5 | mt |
| 1 | IPI00130381 | 4.2 | 3 | Large proline-rich protein BAT3 | unknown |
| 1 | IPI00128783 IPI00373645 | 3.3 | 4 | LDLR dan Low density lipoprotein receptor-related protein 4 | mt |
| 1 | IPI00229517 | 51.9 | 6 | Lectin galactose binding soluble 1 | mt |
| 0.65 | IPI00119907 | 17.5 | 2 | Lectin-related NK cell receptor LY49T | unknown |
| 1 | IPI00170287 | 64.9 | 138 | Leucine rich protein mlrp130 | unknown |
| 1 | IPI00131177 | 37.3 | 31 | Leucine zipper-EF-hand containing transmembrane protein 1 | mt |
| 1 | IPI00223039 IPI00380794 | 11.3 | 7 | Leucyl tRNA synthetase homolog Hypothetical protein | unknown |
| 1 | IPI00123138 | 9.1 | 4 | Leucyl-tRNA synthetase | mt |
| 0.96 | IPI00135010 | 3.8 | 2 | Leukocyte adhesion glycoprotein p15095 alpha integrin subunit | unknown |
| 1 | IPI00130535 | 22.4 | 9 | Lipoamide acyltransferase component of branched-chain alpha-keto acid dehydrogenase complex mitochondrial precursor | mt |
| 1 | IPI00117104 | 23.9 | 7 | Lipoic acid synthetase mitochondrial precursor | mt |
| 0.96 | IPI00315079 IPI00119063 IPI00369995 | 1.6 | 2 | Lipoprotein receptor-related protein AM2 receptor Similar to lipoprotein receptor-related protein | unknown |
| 0.98 | IPI00409513 | 1.9 | 3 | L-NAME induced actin cytoskeletal protein | unknown |
| 1 | IPI00330384 | 16.8 | 7 | LOC276852 protein | unknown |
| 1 | IPI00210503 | 22.5 | 9 | Long-chain-fatty-acid--coa ligase 4 | mt |
| 1 | IPI00170363 | 9.4 | 3 | Long-chain-fatty-acid--coa ligase 5 | mt |
| 1 | IPI00188989 IPI00112549 | 12.9 | 9 | Long-chain-fatty-acid--coa ligase liver isozyme Long-chain-fatty-acid--coa ligase 2 | mt |
| 1 | IPI00336540 | 16.5 | 4 | Lunapark | mt |
| 1 | IPI00228849 IPI00330599 | 31.1 | 13 | LYRIC homolog LYRIC\3D3 | unknown |
| 1 | IPI00117880 | 26.9 | 10 | Lysyl-tRNA synthetase | mt |
| 1 | IPI00120800 | 13.1 | 2 | Mage-k1 | mt |
| 1 | IPI00197696 | 52.1 | 6 | Malate dehydrogenase mitochondrial precursor | unknown |
| 1 | IPI00126120 | 13.4 | 2 | Maleylacetoacetate isomerase | membrane |
| 0.96 | IPI00224294 | 9.6 | 2 | Mannosidase 2 | unknown |
| 0.65 | IPI00197426 | 13.5 | 2 | Mast cell protease 6 precursor | unknown |
| 0.94 | IPI00198493 IPI00169962 IPI00361995 IPI00391645 | 11.4 | 2 | Matrin 3 Similar to matrin 3 Hypothetical protein XP_212889 Ensembl_locations(Chr-bp):15-80558569 | unknown |
| 1 | IPI00189766 | 54.6 | 11 | Membrane associated progesterone receptor component 1 | mt |
| 1 | IPI00112327 | 31.2 | 8 | Metaxin 1 | mt |
| 1 | IPI00225254 | 54 | 17 | Metaxin 2 | unknown |
| 0.97 | IPI00327267 IPI00403447 | 2.1 | 2 | Methionine synthase 5-methyltetrahydrofolate-homocysteine methyltransferase | mt |
| 1 | IPI00320850 | 5.9 | 2 | Methylcrotonyl-coa carboxylase alpha chain mitochondrial precursor | mt |
| 1 | IPI00318210 IPI00362777 IPI00205018 | 7.7 | 2 | Methylmalonate-semialdehyde dehydrogenase Hypothetical protein XP_346778 Methylmalonate-semialdehyde dehydrogenase [acylating] mitochondrial precursor | mt |
| 0.88 | IPI00201479 | 16.7 | 2 | MHC class I antigen | unknown |
| 0.99 | IPI00134390 | 13.2 | 3 | Microsomal dipeptidase precursor | unknown |
| 1 | IPI00130144 IPI00316509 | 26.6 | 9 | Microsomal epoxide hydrolase Epoxide hydrolase 1 | unknown |
| 1 | IPI00112096 | 27.5 | 3 | Microsomal signal peptidase 12 kda subunit | unknown |
| 1 | IPI00313957 | 34.3 | 6 | Microsomal signal peptidase 18 kda subunit | microsome |
| 1 | IPI00130920 | 25.6 | 32 | Microtubule-associated protein 1B | mt |
| 1 | IPI00133167 | 55.2 | 6 | Mitochondria-associated granulocyte macrophage CSF signaling molecule mitochondrial precursor | mt |
| 1 | IPI00133360 IPI00197081 | 10.2 | 2 | Mitochondrial 28S ribosomal protein S14 Similar to mitochondrial ribosomal protein S14 | mt |
| 1 | IPI00126011 | 28.9 | 8 | Mitochondrial 28S ribosomal protein S2 | mt |
| 1 | IPI00110918 | 34.5 | 16 | Mitochondrial 28S ribosomal protein S22 | mt |
| 1 | IPI00315671 | 28.7 | 3 | Mitochondrial 28S ribosomal protein S25 | mt |
| 1 | IPI00110672 | 26.9 | 5 | Mitochondrial 28S ribosomal protein S28 | mt |
| 1 | IPI00132504 | 29.4 | 16 | Mitochondrial 28S ribosomal protein S30 | mt |
| 1 | IPI00120709 | 39.9 | 7 | Mitochondrial 28S ribosomal protein S34 | mt |
| 1 | IPI00315808 | 69.6 | 5 | Mitochondrial 28S ribosomal protein S36 | mt |
| 1 | IPI00118196 | 17.1 | 5 | Mitochondrial 28S ribosomal protein S5 | unknown |
| 1 | IPI00269020 | 33.9 | 4 | Mitochondrial 28S ribosomal protein S6 | mt |
| 1 | IPI00311406 | 18.9 | 7 | Mitochondrial 39s ribosomal protein L39 | unknown |
| 1 | IPI00109293 | 21.8 | 6 | Mitochondrial 39S ribosomal protein L56 | mt |
| 1 | IPI00118227 | 29.7 | 3 | Mitochondrial 60S ribosomal protein L27 | mt |
| 1 | IPI00118235 | 6.9 | 2 | Mitochondrial 60S ribosomal protein L3 | membrane |
| 1 | IPI00131988 IPI00271430 | 21.1 | 3 | Mitochondrial 60s ribosomal protein L49 Mitochondrial ribosomal protein L49 | mt |
| 1 | IPI00131584 | 56.5 | 16 | Mitochondrial carnitine\acylcarnitine carrier protein | mt |
| 1 | IPI00137173 IPI00407430 | 28.6 | 11 | Mitochondrial carrier-like protein 1 isoform B Mitochondrial carrier homolog 1 | mt |
| 1 | IPI00317074 | 54.7 | 31 | Mitochondrial dicarboxylate carrier | mt |
| 1 | IPI00109275 | 43 | 13 | Mitochondrial glutamate carrier 1 | mt |
| 1 | IPI00165902 (IPI00209086) | 53.3 | 13 | Mitochondrial import inner membrane translocase subunit TIM10 | mt |
| 1 | IPI00212651 (IPI00134484) | 36.8 | 5 | Mitochondrial import inner membrane translocase subunit TIM13 A | mt |
| 1 | IPI00129506 | 32.7 | 3 | Mitochondrial import inner membrane translocase subunit TIM17 A | mt |
| 1 | IPI00129504 | 17.4 | 2 | Mitochondrial import inner membrane translocase subunit TIM17 B | mt |
| 1 | IPI00315325 | 23.2 | 3 | Mitochondrial import inner membrane translocase subunit TIM22 | mt |
| 1 | IPI00123712 | 45.9 | 5 | Mitochondrial import inner membrane translocase subunit TIM23 | mt |
| 1 | IPI00204831 IPI00125776 | 22.7 | 3 | Mitochondrial import inner membrane translocase subunit TIM8 A Mitochondrial import inner membrane translocase subunit TIM8 A | mt |
| 1 | IPI00125513 | 60.7 | 4 | Mitochondrial import inner membrane translocase subunit TIM9 A | mt |
| 1 | IPI00125509 | 34 | 5 | Mitochondrial import inner membrane translocase subunit TIM9 B | mt |
| 1 | IPI00208207 IPI00120715 IPI00377374 | 41.7 | 5 | Mitochondrial import receptor subunit TOM20 homolog 1810060K07Rik protein Similar to outer mitochondrial membrane receptor rtom20 | unknown |
| 1 | IPI00315135 | 54.9 | 12 | Mitochondrial import receptor subunit TOM22 homolog | mt |
| 1 | IPI00205076 | 51.5 | 68 | Mitochondrial Lon protease homolog | mt |
| 1 | IPI00125853 | 30.9 | 6 | Mitochondrial ornithine transporter 1 | mt |
| 1 | IPI00120199 | 37.4 | 25 | Mitochondrial processing peptidase alpha subunit mitochondrial precursor | mt |
| 1 | IPI00278781 IPI00229945 IPI00361539 | 21.8 | 3 | Mitochondrial ribosomal protein bmrp36a mitochondrial ribosomal protein L43 Similar to mitochondrial ribosomal protein L43 | mt |
| 1 | IPI00118237 | 34.1 | 13 | Mitochondrial ribosomal protein L1 | mt |
| 1 | IPI00379025 | 19.9 | 6 | Mitochondrial ribosomal protein L44 | mt |
| 1 | IPI00284978 | 23 | 7 | Mitochondrial ribosomal protein L9 | mt |
| 1 | IPI00373759 IPI00320963 | 5.6 | 2 | Mitochondrial ribosomal protein S18A 28S ribosomal protein s18a mitochondrial precursor | cytoplasm |
| 1 | IPI00284934 | 52.5 | 7 | Mitochondrial ribosomal protein S23 | mt |
| 1 | IPI00336292 | 36.9 | 9 | Mitochondrial ribosomal protein S9 | mt |
| 1 | IPI00325525 IPI00312244 IPI00391907 | 6.1 | 2 | Mitochondrial transmembrane gtpase FZO1A Mitofusin 2 Ensembl_locations(Chr-bp):5-164961176 | mt |
| 1 | IPI00109175 | 15.9 | 4 | Mitochondrial uncoupling protein 2 | mt |
| 1 | IPI00133047 IPI00373435 | 31.5 | 2 | Mitogen-activated protein kinase kinase 1 interacting protein 1 Similar to MEK binding partner 1 | mt |
| 0.6 | IPI00117889 IPI00207858 | 4.6 | 2 | Mitogen-activated protein kinase kinase kinase kinase 2 Similar to Rab8-interacting protein | unknown |
| 1 | IPI00377734 | 8.2 | 6 | MKIAA0023 protein | mt |
| 1 | IPI00377728 IPI00223262 | 34.7 | 5 | MKIAA0719 protein Unknown EST | mt |
| 0.6 | IPI00264338 | 5.4 | 2 | MKIAA0734 protein | unknown |
| 0.79 | IPI00399498 | 2 | 2 | MKIAA1151 protein | unknown |
| 1 | IPI00202370 | 11.2 | 9 | Monoamine oxidase | mt |
| 1 | IPI00330688 | 32.2 | 10 | Mrps7 protein | unknown |
| 0.94 | IPI00124893 | 8.7 | 2 | Mto1 | unknown |
| 1 | IPI00129915 | 6.4 | 4 | Multidrug resistance protein | mt |
| 1 | IPI00128152 | 7.8 | 4 | Multidrug resistance protein 1 | mt |
| 0.97 | IPI00203391 (IPI00322096) | 11.5 | 2 | Multifunctional protein ADE2 | unknown |
| 1 | IPI00131222 IPI00372521 | 6 | 2 | Multiple inositol polyphosphate phosphatase Multiple inositol polyphosphate histidine phosphatase 1 | mt |
| 0.97 | IPI00338565 IPI00122438 | 6.6 | 3 | Mutant fibrillin-1 Fibrillin 1 precursor | mt |
| 0.67 | IPI00114809 | 10.3 | 2 | Myc-associated zinc finger protein | unknown |
| 1 | IPI00116372 IPI00212141 | 23.1 | 4 | Myeloid leukemia factor 2 Similar to Myeloid leukemia factor 2 homolog | mt |
| 1 | IPI00209113 | 26.3 | 2 | Myosin heavy chain nonmuscle type A | mt |
| 1 | IPI00137100 (IPI00124281) IPI00408215 | 12.9 | 8 | Myosin IB   Hypothetical protein | mt |
| 1 | IPI00231509 IPI00132705 | 51.2 | 8 | Myosin regulatory light chain 1500001M02Rik protein | unknown |
| 0.89 | IPI00214038 (IPI00118120) IPI00393437 IPI00405881 | 7.2 | 3 | Myosin Va  Ensembl_locations(Chr-bp):8-80002855 Ensembl_locations(Chr-bp):9-77611564 | mt |
| 0.62 | IPI00189725 IPI00392741 | 4.6 | 2 | Myosin Vb Ensembl_locations(Chr-bp):18-71454257 | unknown |
| 0.97 | IPI00125593 IPI00280533 (IPI00194766) | 1.3 | 2 | Myosin VIIA Ensembl_locations(Chr-bp):7-90454888 | mt |
| 0.84 | IPI00138250 IPI00331996 | 2.1 | 3 | Myosin XV Ensembl_locations(Chr-bp):10-46756173 | unknown |
| 0.99 | IPI00209040 | 3.9 | 3 | Myosin-rhogap protein Myr 7 | unknown |
| 1 | IPI00128692 | 44.8 | 8 | NAD(P)-dependent steroid dehydrogenase | unknown |
| 1 | IPI00198720 IPI00109442 | 31.1 | 34 | NAD+-specific isocitrate dehydrogenase a-subunit 1500012E04Rik protein | mt |
| 1 | IPI00315302 | 25.3 | 4 | NADH dehydrogenase (ubiquinone) 1 alpha subcomplex 2 | mt |
| 1 | IPI00226687 | 14.8 | 5 | NADH dehydrogenase (ubiquinone) 1 alpha subcomplex assembly factor 1 | mt |
| 1 | IPI00121288 | 33.5 | 8 | NADH dehydrogenase (ubiquinone) 1 beta subcomplex 10 | mt |
| 1 | IPI00341550 | 14.2 | 2 | NADH dehydrogenase subunit 1 | mt |
| 1 | IPI00170093 | 36.3 | 11 | NADH dehydrogenase:ubiquinone Fe-S protein 8 | mt |
| 1 | IPI00132390 | 39.5 | 5 | NADH:ubiquinone oxidoreductase B15 subunit | mt |
| 1 | IPI00231662 | 67.2 | 8 | NADH-cytochrome b5 reductase | mt |
| 1 | IPI00207072 | 37.9 | 3 | NADH-ubiquinone oxidoreductase 13 kda-A subunit mitochondrial precursor | mt |
| 1 | IPI00331332 | 29.7 | 6 | NADH-ubiquinone oxidoreductase 13 kda-B subunit | mt |
| 1 | IPI00121309 IPI00225329 | 47.1 | 16 | NADH-ubiquinone oxidoreductase 30 kda subunit mitochondrial precursor NADH dehydrogenase | mt |
| 1 | IPI00120212 | 51.2 | 20 | NADH-ubiquinone oxidoreductase 39 kda subunit mitochondrial precursor | mt |
| 1 | IPI00116748 | 33.2 | 12 | NADH-ubiquinone oxidoreductase 42 kda subunit mitochondrial precursor | mt |
| 1 | IPI00308882 | 39.8 | 27 | NADH-ubiquinone oxidoreductase 75 kda subunit | mt |
| 1 | IPI00133399 | 31.3 | 5 | NADH-ubiquinone oxidoreductase B14 subunit | mt |
| 1 | IPI00132216 IPI00365436 | 13.1 | 2 | NADH-ubiquinone oxidoreductase B9 subunit Similar to NADH-ubiquinone oxidoreductase B9 subunit (Complex I-B9) (CI-B9) | mt |
| 1 | IPI00116169 IPI00355228 | 4.9 | 2 | NADH-ubiquinone oxidoreductase chain 2 NADH dehydrogenase subunit 2 | mt |
| 1 | IPI00116843 | 9.8 | 7 | NADH-ubiquinone oxidoreductase chain 4 | mt |
| 1 | IPI00116869 IPI00355281 | 6.8 | 3 | NADH-ubiquinone oxidoreductase chain 5 NADH dehydrogenase subunit 5 | mt |
| 1 | IPI00114246 | 28.5 | 4 | NADH-ubiquinone oxidoreductase ESSS subunit mitochondrial precursor | mt |
| 1 | IPI00122496 | 47.4 | 20 | NADPH:adrenodoxin oxidoreductase  mitochondrial precursor | mt |
| 1 | IPI00223855 | 41.5 | 31 | NADPH--cytochrome P450 reductase | mt |
| 1 | IPI00111831 (IPI00121297) IPI00371266 | 19.5 | 3 | NASCENT polypeptide-associated complex alpha polypeptide  Similar to alpha NAC\1.9.2. Protein | nuleus |
| 1 | IPI00117416 | 34.3 | 9 | Neighbor of COX4 | unknown |
| 0.97 | IPI00207095 | 4.6 | 4 | Nesprin-1 | unknown |
| 1 | IPI00210635 IPI00311934 | 3.5 | 2 | N-ethylmaleimide sensitive factor Vesicle-fusing atpase | mt |
| 0.62 | IPI00119840 | 11 | 3 | Netrin 4 | unknown |
| 0.88 | IPI00127654 | 10.1 | 4 | Neurogenic locus notch homolog protein 3 precursor | unknown |
| 1 | IPI00314829 | 16.1 | 8 | Neurolysin (metallopeptidase M3 family) | mt |
| 1 | IPI00230882 | 15.1 | 7 | Neurolysin mitochondrial precursor | mt |
| 0.89 | IPI00309199 | 13 | 4 | Neurotrypsin precursor | unknown |
| 1 | IPI00135130 | 24.2 | 4 | Neutral amino acid transporter A | mt |
| 1 | IPI00113389 | 18.6 | 7 | Niban | mt |
| 1 | IPI00309964 | 29.7 | 33 | Nicotinamide nucleotide transhydrogenase | mt |
| 1 | IPI00115824 IPI00364134 | 32.7 | 12 | Nipsnap1 protein Similar to NIPSNAP1 protein | unknown |
| 1 | IPI00115827 | 21 | 5 | Nipsnap2 protein | mt |
| 1 | IPI00314452 | 31.2 | 7 | NIPSNAP-related protein | cytoplasm |
| 1 | IPI00311072 | 23.5 | 7 | Nitrogen fixation gene 1 | unknown |
| 1 | IPI00320716 (IPI00125492) | 26.3 | 7 | NOGO-interacting mitochondrial protein | mt |
| 1 | IPI00123181 | 30 | 7 | Nonmuscle heavy chain myosin II-A | unknown |
| 1 | IPI00338604 | 33.6 | 56 | Non-muscle myosin heavy chain 10 | mt |
| 1 | IPI00320016 | 26.4 | 8 | Non-POU-domain-containing octamer-binding protein | nuleus |
| 1 | IPI00134131 | 4 | 2 | Nonspecific lipid-transfer protein mitochondrial precursor | mt |
| 1 | IPI00406664 | 8.3 | 4 | N-rap | unknown |
| 0.92 | IPI00214262 IPI00399450 IPI00387232 IPI00399449 | 11.4 | 2 | NSFL1 cofactor p47 Splice isoform 2 of Q9CZ44 NSFL1 cofactor p47 Splice isoform 3 of Q9CZ44 NSFL1 cofactor p47 Splice isoform 1 of Q9CZ44 NSFL1 cofactor p47 | nucleus |
| 1 | IPI00368473 | 4 | 5 | Nuclear mitotic apparatus protein 1 | mt |
| 1 | IPI00120467 | 8.6 | 6 | Nuclear pore complex protein Nup155 | unknown |
| 1 | IPI00342158 IPI00195875 | 2.7 | 2 | Nuclear pore membrane glycoprotein POM210 Integral membrane glycoprotein gp210 precursor | mt |
| 1 | IPI00121276 | 55.8 | 21 | Nuclear receptor binding factor 1 | mt |
| 1 | IPI00210183 | 28.2 | 12 | Nuclear receptor binding factor-1 | mt |
| 1 | IPI00170126 | 26.2 | 26 | Nuclear transplantation upregulated protein 1 | mt |
| 1 | IPI00120886 (IPI00400653) IPI00388522 (IPI00200809) | 21.9 | 5 | Nuclease sensitive element binding protein 1  Ensembl_locations(Chr-bp):5-139865150 | mt |
| 1 | IPI00132314 | 9.2 | 2 | Nucleobindin 1 precursor | mt |
| 1 | IPI00318048 | 5.2 | 2 | Nucleolar protein Nop56 | mt |
| 1 | IPI00120691 | 13.6 | 8 | Nucleolar RNA helicase II | nuleus |
| 1 | IPI00317794 | 14.1 | 9 | Nucleolin | mt |
| 1 | IPI00189770 (IPI00225192) | 9 | 2 | Nucleoporin p54 | mt |
| 1 | IPI00127417 (IPI00325189)  IPI00403584 IPI00194404 (IPI00131459) IPI00408489 | 19.1 | 8 | Nucleoside diphosphate kinase B  Hypothetical protein Nucleoside diphosphate kinase A  Down syndrome cell adhesion molecule-like protein | mt |
| 1 | IPI00123199 (IPI00404665) IPI00407932 | 24 | 6 | Nucleosome assembly protein 1-like 1  Ensembl_locations(Chr-bp):10-114043292 | mt |
| 1 | IPI00380333 | 9.5 | 2 | Nur77 downstream protein 2 | mt |
| 1 | IPI00337099 | 46.2 | 28 | Nyggf2 protein | unknown |
| 0.68 | IPI00270041 | 3.8 | 2 | Odd Oz\ten-m homolog 1 | unknown |
| 0.94 | IPI00201319 | 29 | 2 | OL-64 protein | mt |
| 0.75 | IPI00126457 | 12.7 | 2 | Olfactory receptor MOR237-2 | unknown |
| 1 | IPI00109108 | 16.7 | 10 | Oligosaccharyl transferase STT3 subunit homolog | mt |
| 0.9 | IPI00131869 IPI00368374 | 9.2 | 2 | OPA-containing protein 1 Similar to OPA-containing protein 1 | unknown |
| 1 | IPI00116750 | 26 | 3 | Opioid receptor sigma 1 | unknown |
| 1 | IPI00129178 | 62.2 | 42 | Ornithine aminotransferase mitochondrial precursor | mt |
| 0.62 | IPI00127131 | 4.5 | 2 | Osa1 nuclear protein | unknown |
| 1 | IPI00133374 | 15.6 | 3 | Oxidative stress induced | membrane |
| 1 | IPI00331361 IPI00330812 | 26.3 | 38 | P160 myb-binding protein MYB binding protein (P160) 1a | mt |
| 0.99 | IPI00127233 IPI00121491 | 4.1 | 2 | P53-related protein kinase Similar to mnori-2p | unknown |
| 0.99 | IPI00331082 IPI00320532 IPI00205097 | 10.8 | 2 | P62 ras-GAP associated phosphoprotein Src associated in mitosis 68 kda Nuclear RNA binding protein Sam68 | mt |
| 0.67 | IPI00211116 IPI00327483 | 9.5 | 2 | PCTAIRE-1b protein kinase PCTAIRE-1a protein kinase | unknown |
| 1 | IPI00135239 | 4.2 | 3 | PDZ domain actin binding protein Shroom | mt |
| 0.97 | IPI00204396 IPI00133555 | 16.1 | 2 | Peachy 1110017C15Rik protein | unknown |
| 0.62 | IPI00128789 IPI00360319 | 3 | 2 | Pecanex-like protein 3 Similar to pecanex-like 3 | unknown |
| 1 | IPI00118545 | 52.5 | 17 | PEG1\MEST protein | unknown |
| 1 | IPI00187740 | 47.9 | 3 | Peptidyl-prolyl cis-trans isomerase A | mt |
| 1 | IPI00135686 | 46.2 | 14 | Peptidyl-prolyl cis-trans isomerase B precursor | ER |
| 1 | IPI00116228 | 33 | 5 | Peptidyl-prolyl cis-trans isomerase mitochondrial precursor | mt |
| 1 | IPI00121788 | 29.1 | 4 | Peroxiredoxin 1 | unknown |
| 1 | IPI00117910 | 17.2 | 3 | Peroxiredoxin 2 | unknown |
| 1 | IPI00116254 IPI00208209 | 34.8 | 9 | Peroxiredoxin 4 prx IV | mt |
| 1 | IPI00129517 | 51 | 9 | Peroxiredoxin 5 mitochondrial precursor | mt |
| 1 | IPI00322931 | 14 | 3 | Peroxisomal 3 2-trans-enoyl-coa isomerase | mt |
| 1 | IPI00406227 IPI00309365 | 9.4 | 2 | Peroxisomal acyl-coa thioesterase 2 Peroxisomal acyl-coenzyme A thioester hydrolase 1 | unknown |
| 1 | IPI00135680 (IPI00201664) | 25.3 | 4 | Peroxisomal assembly protein PEX3 | nuleus |
| 1 | IPI00223004 | 29.5 | 11 | Peroxisomal CA-dependent solute carrier homolog | mt |
| 1 | IPI00127237 | 32.2 | 11 | Peroxisomal membrane protein PEX14 | Peroxisome |
| 1 | IPI00119466 | 11.1 | 4 | Peroxisomal membrane protein PMP34 | unknown |
| 1 | IPI00331596 | 28.5 | 6 | Peroxisomal trans 2-enoyl coa reductase | mt |
| 1 | IPI00263874 IPI00199865 (IPI00403321) IPI00311594 | 15.6 | 2 | Phenylalanyl-tRNA synthetase alpha chain Similar to 0610012A19Rik protein  Similar to phenylalanine-tRNA synthetase-like | unknown |
| 1 | IPI00124771 | 56.9 | 28 | Phosphate carrier protein mitochondrial precursor | mt |
| 1 | IPI00221857 | 11.9 | 9 | Phosphatidylglycerophosphate synthase homolog | mt |
| 1 | IPI00125681 | 47.4 | 21 | Phosphatidylinositol glycan class S homolog | membrane |
| 1 | IPI00310099 | 21.2 | 9 | Phosphatidylserine decarboxylase proenzyme | mt |
| 1 | IPI00223060 | 40.5 | 25 | Phosphoenolpyruvate carboxykinase | mt |
| 1 | IPI00408407 IPI00231425 | 16.2 | 3 | Phosphoglycerate mutase 1 phosphoglycerate mutase 1 | mt |
| 1 | IPI00229523 | 17.3 | 3 | Phosphotidylinositol transfer protein beta | mt |
| 1 | IPI00191485 | 3 | 3 | Pkd1 | mt |
| 1 | IPI00124661 | 15.7 | 2 | PL6 protein | mt |
| 0.84 | IPI00172310 | 5.1 | 3 | Pleckstrin homology domain containing family E (with leucine rich repeats) member 1 | unknown |
| 1 | IPI00400215 IPI00399735 IPI00400209 IPI00400212 IPI00400213 IPI00400214 IPI00400207 IPI00400208 IPI00400211 | 5.1 | 13 | Plectin 1 isoform 11 plectin 1 isoform 1 plectin 1 isoform 5 plectin 1 isoform 7 plectin 1 isoform 8 plectin 1 isoform 10 plectin 1 isoform 2 plectin 1 isoform 3 plectin 1 isoform 6 | mt |
| 1 | IPI00321143 | 18.3 | 2 | Poly(rc)-binding protein 3 | mt |
| 1 | IPI00124287 IPI00331552 | 20.6 | 10 | Polyadenylate-binding protein 1 Poly A binding protein cytoplasmic 1 | mt |
| 0.68 | IPI00318010 | 3.1 | 2 | Polycystic kidney disease 1-like 2 | unknown |
| 0.63 | IPI00112858 | 3.4 | 2 | Polydom protein precursor | unknown |
| 1 | IPI00321923 | 36.5 | 35 | Polynucleotide phosphorylase | mt |
| 1 | IPI00308771 IPI00371478  IPI00268187 | 21.3 | 7 | Polypeptide galnac transferase-T2 Similar to UDP-N-acetyl-alpha-D-galactosamine:polypeptide N-acetylgalactosaminyltransferase 2 Hypothetical protein | golgi |
| 1 | IPI00136883 IPI00390239 (IPI00231555) IPI00123037 IPI00390801 | 36.9 | 17 | Polypyrimidine tract-binding protein 1 Splice isoform PYBP2 of Q00438   Hypothetical protein Ensembl_locations(Chr-bp):7-11355539 | mt |
| 0.68 | IPI00224304 | 22.9 | 2 | PR\SET domain-containing protein 07 homolog | unknown |
| 1 | IPI00109891 | 7.5 | 4 | Probable cation-transporting atpase 13A | mt |
| 0.99 | IPI00226624 (IPI00227592) | 9 | 2 | Probable G protein-coupled receptor GPR22 homolog | unknown |
| 1 | IPI00127406 | 23.5 | 15 | Procollagen-lysine 2-oxoglutarate 5-dioxygenase 3 precursor | mt |
| 1 | IPI00207871 (IPI00127407) | 8.5 | 2 | Procollagen-lysine2-oxoglutarate 5-dioxygenase 1 precursor | mt |
| 1 | IPI00231358 IPI00224740 | 57.9 | 11 | Profilin Profilin 1 | mt |
| 1 | IPI00129577 | 53.4 | 14 | Programmed cell death protein 8 mitochondrial precursor | unknown |
| 1 | IPI00133440 (IPI00211756) | 65.1 | 37 | Prohibitin | mt |
| 1 | IPI00124980 | 20.9 | 4 | Prolactin regulatory element-binding protein | mt |
| 1 | IPI00119305 | 40.9 | 17 | Proliferation-associated protein 2G4 | mt |
| 0.94 | IPI00400661 | 3.5 | 2 | Proline rich synapse associated protein 1 isoform E | unknown |
| 1 | IPI00131548 | 9.1 | 2 | Proline synthetase co-transcribed bacterial homolog protein | unknown |
| 1 | IPI00209863 IPI00272381 IPI00399959 | 6.2 | 2 | Prolyl 4-hydroxylase alpha-1 subunit precursor Splice isoform 1 of Q60715 Prolyl 4-hydroxylase alpha-1 subunit precursor Splice isoform 2 of Q60715 Prolyl 4-hydroxylase alpha-1 subunit precursor | membrane |
| 1 | IPI00314972 IPI00330523 | 7.3 | 3 | Propionyl coa-carboxylase alpha-subunit Propionyl-coenzyme A carboxylase alpha polypeptide | mt |
| 1 | IPI00320736 IPI00119688 | 13.1 | 3 | Propionyl-coa carboxylase beta chain 1300012P06Rik protein | mt |
| 0.99 | IPI00128389 | 3 | 2 | Prostaglandin G\H synthase 1 precursor | mt |
| 1 | IPI00319518 | 52 | 86 | Protease serine 15 | unknown |
| 1 | IPI00231757 IPI00404117 (IPI00318970) | 17.6 | 3 | Proteasome (prosome macropain) subunit alpha type 2 Proteasome subunit alpha type 2 | mt |
| 0.99 | IPI00191749 (IPI00113845) | 15.8 | 2 | Proteasome subunit beta type 1 | mt |
| 0.85 | IPI00330709 | 5.4 | 2 | Protein c14orf102 homolog | mt |
| 1 | IPI00317709 | 11.6 | 5 | Protein c14orf127 homolog | unknown |
| 1 | IPI00132456 | 18.4 | 4 | Protein c14orf166 homolog | unknown |
| 1 | IPI00111323 | 6.8 | 2 | Protein c20orf108 homolog | unknown |
| 1 | IPI00330679 | 33.3 | 9 | Protein c20orf116 homolog precursor | unknown |
| 1 | IPI00165731 IPI00196965 | 13.3 | 4 | Protein c20orf22 homolog Ensembl_locations(Chr-bp):3-141375054 | unknown |
| 1 | IPI00132768 | 50 | 7 | Protein c6orf53 homolog | unknown |
| 1 | IPI00133744 | 23.1 | 2 | Protein c6orf66 homolog | mt |
| 1 | IPI00221608 | 51.6 | 33 | Protein CGI-51 homolog | unknown |
| 1 | IPI00309047 | 17.5 | 9 | Protein disulfide isomerase A4 precursor | mt |
| 1 | IPI00222496 | 37.3 | 15 | Protein disulfide isomerase A6 precursor | ER |
| 1 | IPI00133522 IPI00122815 | 50.9 | 36 | Protein disulfide isomerase precursor Similar to protein disulfide isomerase | mt |
| 1 | IPI00133612 IPI00366247 | 29 | 9 | Protein KIAA0103 homolog Similar to RIKEN cDNA 4921531G14 | unknown |
| 1 | IPI00230005 IPI00227900  (IPI00200013) | 7.2 | 2 | Protein kinase camp dependent catalytic alpha Splice isoform 2 of P05132 camp-dependent protein kinase alpha-catalytic subunit | mt |
| 0.69 | IPI00285485 | 12.3 | 2 | Protein kinase C-binding protein NELL2 precursor | mt |
| 1 | IPI00310091 | 18.8 | 6 | Protein phosphatase 2 | mt |
| 0.98 | IPI00114802 (IPI00201403) | 12.3 | 2 | Protein phosphatase 2C alpha isoform | mt |
| 1 | IPI00222447 IPI00369540 | 20.5 | 6 | Protein PM1 Similar to RIKEN cDNA 5730466P16 | mt |
| 1 | IPI00222429 | 31.5 | 39 | Protein PM5 precursor homolog | unknown |
| 1 | IPI00226882 IPI00231341 | 16.6 | 7 | Protein transport protein Sec61 alpha subunit isoform 1 Sec61 alpha subunit homolog | unknown |
| 1 | IPI00133132 (IPI00310105) | 12.8 | 3 | Protein tyrosine kinase 2 beta | mt |
| 1 | IPI00388450 | 8.3 | 5 | Protein tyrosine phosphatase receptor type U | mt |
| 1 | IPI00123039 | 20.8 | 4 | Protein-tyrosine phosphatase non-receptor type 1 | mt |
| 0.95 | IPI00207544 | 2.6 | 4 | Protocadherin Fat 2 precursor | unknown |
| 0.99 | IPI00109672 IPI00201455 | 7.9 | 2 | Proto-oncogene tyrosine-protein kinase YES Protein tyrosine kinase c-Yes | nuleus |
| 1 | IPI00119116 | 33.1 | 9 | Protoporphyrinogen oxidase | cytoplasm |
| 1 | IPI00129430 | 21.2 | 9 | PTB-associated splicing factor | nucleus |
| 1 | IPI00133270 | 41.9 | 19 | Putative ATP-dependent Clp protease proteolytic subunit mitochondrial precursor | unknown |
| 1 | IPI00113606 | 44.7 | 18 | Putative lipid kinase | unknown |
| 1 | IPI00121974 IPI00203757 | 15.4 | 4 | Putative membrane protein Similar to RIKEN cDNA 1190006A08 | unknown |
| 1 | IPI00128818 IPI00361832 | 23 | 15 | Putative pre-mRNA splicing factor RNA helicase Similar to Putative pre-mRNA splicing factor RNA helicase (DEAH box protein 15) | mt |
| 1 | IPI00130883 IPI00378187 (IPI00367437) | 32.7 | 2 | Putative RNA-binding protein 3 RNA binding motif protein 3 | unknown |
| 1 | IPI00135512 | 24.2 | 5 | Putative secreted protein ZSIG9 | unknown |
| 1 | IPI00119219 | 64.4 | 29 | Putative steroid dehydrogenase KIK-I | unknown |
| 0.81 | IPI00129561 | 2.5 | 3 | Putative ubiquitin-specific protease | unknown |
| 1 | IPI00114710 | 21.8 | 24 | Pyruvate carboxylase mitochondrial precursor | mt |
| 1 | IPI00222767 | 29.1 | 12 | Pyruvate dehydrogenase protein X component | mt |
| 1 | IPI00153792 | 74.8 | 5 | Qil1 | mt |
| 1 | IPI00122565 | 21.6 | 9 | Rab GDP dissociation inhibitor beta-2 | mt |
| 1 | IPI00330031 IPI00372642 IPI00130112 | 15.4 | 3 | RAB34 member of RAS oncogene family Similar to Rab family small gtpase Rah Ras-related protein Rab-34 | mt |
| 1 | IPI00404579 IPI00388139 IPI00191761 IPI00224518 | 36.6 | 7 | RAB5C Ensembl_locations(Chr-bp):10-89667740 Similar to Rab5c protein Ras-related protein Rab-5C | unknown |
| 1 | IPI00108335 IPI00392420 (IPI00200675) | 3.6 | 3 | RAD50 Ensembl_locations(Chr-bp):10-39121980 | mt |
| 1 | IPI00337844 | 4.2 | 7 | RAN binding protein 2 | mt |
| 0.99 | IPI00129470 | 11.9 | 3 | Ran gtpase-activating protein 1 | mt |
| 1 | IPI00122935 | 13.6 | 15 | Ras gtpase-activating-like protein IQGAP1 | mt |
| 1 | IPI00407954 | 50 | 14 | RAS related protein 1b | mt |
| 1 | IPI00130118 | 32 | 5 | Ras-related protein Rab-10 | unknown |
| 1 | IPI00135869 IPI00192283 | 44 | 13 | Ras-related protein Rab-11B GTP-binding protein RAB11B | membrane |
| 0.86 | IPI00112374 | 29.9 | 2 | Ras-related protein Rab-27A | unknown |
| 1 | IPI00137227 | 59 | 18 | Ras-related protein Rab-2A | mt |
| 1 | IPI00316495 (IPI00209150) | 24.7 | 2 | Ras-related protein Rab-3C | mt |
| 1 | IPI00408892 (IPI00215564) | 76.3 | 20 | Ras-related protein Rab-7 | mt |
| 1 | IPI00200427 IPI00127820 | 18.9 | 2 | Ras-related protein Rab-9A Small GTP binding protein | ER |
| 1 | IPI00203575 (IPI00124282) | 17.5 | 3 | Ras-related protein Ral-A | mt |
| 1 | IPI00331173 | 12.6 | 6 | RAT NBP60 homolog | unknown |
| 1 | IPI00407167 | 9.6 | 2 | Reticulon 3 | ER |
| 1 | IPI00110659 | 21.9 | 6 | Retinal short-chain dehydrogenase\reductase 4 precursor | unknown |
| 1 | IPI00136098 | 38.9 | 11 | Retinol dehydrogenase 11 | mt |
| 1 | IPI00229040 | 36.8 | 6 | Retinol dehydrogenase 13 | mt |
| 1 | IPI00322312 IPI00196994 | 42.2 | 8 | Rho GDP-dissociation inhibitor 1 Similar to rhogdi-1 | mt |
| 0.64 | IPI00191533 | 3.1 | 2 | Rho guanine nucleotide exchange factor 11 | unknown |
| 0.86 | IPI00118748 | 5.9 | 2 | Rho-related BTB domain-containing protein 1 | unknown |
| 0.74 | IPI00336345 IPI00360619 | 3.9 | 3 | Ribonuclease III Similar to Ribonuclease III (RNAse III) (p241) | unknown |
| 1 | IPI00226073 IPI00372427 (IPI00210357) (IPI00131508) (IPI00352639) | 10.3 | 2 | Ribonucleoprotein F homolog Similar to ribonucleoprotein F | mt |
| 1 | IPI00323075 | 48.1 | 44 | Ribophorin II | mt |
| 1 | IPI00224505 (IPI00230916) | 24.2 | 8 | Ribosomal protein L13 | unknown |
| 1 | IPI00231124 (IPI00223217) | 15.3 | 4 | Ribosomal protein L13A | unknown |
| 1 | IPI00231246 IPI00133185 (IPI00109199) IPI00403034 (IPI00373497) | 21.4 | 5 | Ribosomal protein L14 3100001N19Rik protein  Similar to RIKEN cDNA 3100001N19 | mt |
| 1 | IPI00231435 IPI00315548 IPI00409323 IPI00400601 IPI00406640 | 33.1 | 4 | Ribosomal protein L21 Rpl21 protein Ensembl_locations(Chr-bp):18-82221571 Ensembl_locations(Chr-bp):9-58532573 Ensembl_locations(Chr-bp):Un_random_NT_084314-2 | unknown |
| 1 | IPI00136310 | 47.3 | 9 | Ribosomal protein L23 | mt |
| 1 | IPI00231360 (IPI00122421) IPI00368970 (IPI00345988) | 28.8 | 6 | Ribosomal protein L27  Similar to ribosomal protein L27 | unknown |
| 1 | IPI00315166 IPI00338939 (IPI00343058) IPI00405007 | 24.3 | 4 | Ribosomal protein l27a Similar to ribosomal protein l27a  60S ribosomal protein l27a | mt |
| 1 | IPI00321170 | 30.8 | 22 | Ribosomal protein L3 | mt |
| 1 | IPI00231346 (PI00222549)  IPI00377381 IPI00399898 IPI00351677 | 54.6 | 12 | Ribosomal protein L30  Similar to ribosomal protein L30  Ensembl_locations(Chr-bp):15-34852140;15-34852140 Ensembl_locations(Chr-bp):X-61399558 | mt |
| 1 | IPI00230623 IPI00377984 (IPI00196808) IPI00270924 (IPI00231778) | 28.7 | 8 | Ribosomal protein L32 Similar to 60S ribosomal protein L32   Ensembl_locations(Chr-bp):8-4372436 | unknown |
| 1 | IPI00111784 | 11.4 | 3 | Ribosomal protein mitochondrial L26 | mt |
| 1 | IPI00134835 IPI00133741 | 39.6 | 10 | Ribosomal protein mitochondrial L7 S cells  RIKEN cDNA full-length enriched library clone:2410029F03 full insert sequence | mt |
| 1 | IPI00125901 (PI00366014) | 29.8 | 12 | Ribosomal protein S13 | cytoplasm |
| 1 | IPI00231692 IPI00372695 IPI00404376 (IPI00361666) (IPI00378844) IPI00406431 (IPI00379577) (IPI00319231) (IPI00381526) | 15.3 | 3 | Ribosomal protein S15 Similar to ribosomal protein S15 Ensembl_locations(Chr-bp):18-64473599   Ensembl_locations(Chr-bp):6-58481592 | mt |
| 1 | IPI00231474 (PI00230660) | 45.4 | 9 | Ribosomal protein s15a | mt |
| 1 | IPI00230767 IPI00231288 | 32.4 | 6 | Ribosomal protein S16 Similar to 40S ribosomal protein S16 | mt |
| 1 | IPI00324983 IPI00263129 IPI00368558 IPI00381546 | 32.8 | 4 | Ribosomal protein S17 40S ribosomal protein S17 Similar to 40S RIBOSOMAL PROTEIN S17 Similar to ribosomal protein S17 | unknown |
| 1 | IPI00124709 IPI00210965 | 28.9 | 4 | Ribosomal protein S27-like 40S ribosomal protein S27 | unknown |
| 1 | IPI00126351 IPI00121149 | 21.4 | 33 | Ribosome binding protein 1 Splice isoform 3 of Q99PL5 Ribosome-binding protein 1 | mt |
| 1 | IPI00115117 | 55.8 | 8 | RIKEN cDNA 0610038F01 gene | unknown |
| 1 | IPI00228719 IPI00366571 | 10.4 | 3 | RIKEN cDNA 1110001C20 gene Ac2-190 | unknown |
| 1 | IPI00400330 | 10.6 | 3 | RIKEN cDNA 1110001J12 | unknown |
| 1 | IPI00409243 IPI00367950 IPI00137045 IPI00212104 | 10.1 | 2 | RIKEN cDNA 1110020G09 Similar to hypothetical protein FLJ30596 4933430B08Rik protein Ensembl_locations(Chr-bp):2-58654444 | mt |
| 1 | IPI00381394 | 13.7 | 14 | RIKEN cDNA 1110055E19 | unknown |
| 1 | IPI00279562 IPI00126857 | 8.7 | 3 | RIKEN cDNA 1190005P08 gene Hypothetical protein | mt |
| 1 | IPI00133016 IPI00387392 | 5 | 2 | RIKEN cDNA 1200007B05 gene 1200007B05Rik protein | mt |
| 1 | IPI00400137 IPI00403336 IPI00117657 | 16.9 | 12 | RIKEN cDNA 1200011N24 MKIAA0567 protein Dynamin-like 120 kda protein mitochondrial precursor | mt |
| 1 | IPI00126634 IPI00203398 | 43.8 | 15 | RIKEN cDNA 1300003F06 gene Similar to Polymerase delta interacting protein 38 | unknown |
| 1 | IPI00108431 | 30.3 | 12 | RIKEN cDNA 1500001M20 | unknown |
| 1 | IPI00322018 | 20.3 | 4 | RIKEN cDNA 1700023M03 gene | unknown |
| 1 | IPI00377592 | 5.9 | 2 | RIKEN cDNA 1810024J13 | mt |
| 1 | IPI00112126 | 25.7 | 3 | RIKEN cDNA 1810027O10 | unknown |
| 1 | IPI00317684 | 11.6 | 4 | RIKEN cDNA 1810074P20 | mt |
| 0.65 | IPI00322346 | 10.6 | 2 | RIKEN cDNA 2010300C02 | unknown |
| 1 | IPI00396833 | 26.5 | 13 | RIKEN cDNA 2210023C10 | unknown |
| 1 | IPI00265386 | 31.7 | 13 | RIKEN cDNA 2210404D11 | unknown |
| 1 | IPI00162942 | 27.7 | 8 | RIKEN cDNA 2300004O14 gene | unknown |
| 1 | IPI00169862 | 9.3 | 3 | RIKEN cDNA 2310005O14 gene | mt |
| 1 | IPI00341302 | 17.7 | 4 | RIKEN cDNA 2310030N02 | mt |
| 1 | IPI00227171 | 8.6 | 2 | RIKEN cDNA 2310051N18 | unknown |
| 1 | IPI00338458 | 10.2 | 4 | RIKEN cDNA 2810422B04 | unknown |
| 0.91 | IPI00309792 | 1.3 | 2 | RIKEN cDNA 2810441C07 | unknown |
| 1 | IPI00405108 | 10.2 | 4 | RIKEN cDNA 2900072D10 gene | mt |
| 1 | IPI00226958 | 16.3 | 11 | RIKEN cDNA 3100002P13 | unknown |
| 1 | IPI00274656 | 31.9 | 21 | RIKEN cDNA 3110004O18 | mt |
| 1 | IPI00311445 IPI00197180 | 37 | 18 | RIKEN cDNA 3732409C05 Similar to hypothetical protein FLJ22728 | mt |
| 0.65 | IPI00222413 | 3.3 | 3 | RIKEN cDNA 4432411E13 | unknown |
| 1 | IPI00321665 IPI00123139 | 16.8 | 3 | RIKEN cDNA 4733401H18 gene 2P1 protein | unknown |
| 1 | IPI00337930 | 5.1 | 4 | RIKEN cDNA 4930432B04 | unknown |
| 0.83 | IPI00229866 IPI00373079 | 5.6 | 2 | RIKEN cDNA 4930570G11 Similar to KIAA0853 protein | unknown |
| 0.8 | IPI00330139 | 6.4 | 3 | RIKEN cDNA 5530401C11 | unknown |
| 1 | IPI00223047 | 41.6 | 40 | RIKEN cDNA 5630400A09 gene | unknown |
| 1 | IPI00230456 IPI00277184 | 28.2 | 9 | RIKEN cDNA 5730502D15 Hypothetical protein PP2447 homolog | unknown |
| 1 | IPI00229762 | 7.9 | 2 | RIKEN cDNA 6330590F17 | mt |
| 0.85 | IPI00341975 | 10.3 | 2 | RIKEN cDNA 6430598A04 gene | mt |
| 1 | IPI00329915 | 16.8 | 5 | RIKEN cDNA 9030408N13 gene | unknown |
| 1 | IPI00153702 | 53.8 | 10 | RIKEN cDNA A230072I16 gene | unknown |
| 0.75 | IPI00378378 | 1.8 | 2 | RIKEN cDNA A330102H22 | unknown |
| 0.96 | IPI00345676 | 2.9 | 4 | RIKEN cDNA A730019I05 gene | unknown |
| 0.99 | IPI00322033 | 12.5 | 3 | RIKEN cDNA A730055F12 | unknown |
| 0.92 | IPI00280103 | 3.9 | 2 | RIKEN cDNA A930021H16 | mt |
| 1 | IPI00380805 | 2.1 | 3 | RIKEN cDNA B630009I04 | unknown |
| 0.82 | IPI00272398 | 3.7 | 2 | RIKEN cDNA B930011H20 gene | unknown |
| 0.68 | IPI00114424 | 2.2 | 2 | RIKEN cDNA D930036F22 gene | unknown |
| 1 | IPI00330363 IPI00340913 IPI00354363 IPI00363949 | 38.9 | 16 | Rpl7a protein 60S ribosomal protein l7a Similar to Rpl7a protein Similar to 60S ribosomal protein L7a (Surfeit locus protein 3) (PLA-X polypeptide) | mt |
| 0.92 | IPI00195015 | 11.9 | 2 | RRNA promoter binding protein | unknown |
| 1 | IPI00224219 | 30.2 | 16 | RSLY1P homolog | unknown |
| 1 | IPI00308069 | 24.3 | 14 | Rtn4 | mt |
| 1 | IPI00133985 (IPI00212268) | 14.3 | 4 | Ruvb-like 1 | mt |
| 1 | IPI00118333 | 3.4 | 4 | RW1 protein | mt |
| 1 | IPI00109221 | 24 | 13 | SAC1 (supressor of actin mutations 1 homolog)-like | unknown |
| 0.79 | IPI00124120 IPI00373012 | 3.5 | 2 | Sacsin Similar to sacsin | mt |
| 1 | IPI00230440 (IPI00231706) | 6.5 | 2 | S-adenosylhomocysteine hydrolase | mt |
| 0.8 | IPI00338954 | 7.9 | 3 | SAM and SH3 domains containing protein 1 | unknown |
| 0.86 | IPI00229424 | 4.6 | 4 | SCO-spondin | unknown |
| 1 | IPI00207738 IPI00123349 | 4.1 | 3 | SEC23A Protein transport protein Sec23A | mt |
| 1 | IPI00330857 | 19.7 | 12 | Sec63 | mt |
| 0.88 | IPI00329862 | 3.6 | 2 | Secreted protein SST3 | unknown |
| 1 | IPI00205606 IPI00131143 IPI00224252 | 9.5 | 3 | Sel1 (suppressor of lin-12) 1 homolog Splice isoform 1 of Q9Z2G6 Sel-1 homolog precursor Splice isoform 2 of Q9Z2G6 Sel-1 homolog precursor | mt |
| 1 | IPI00310205 | 9.8 | 3 | Selenocysteine-specific elongation factor | mt |
| 0.89 | IPI00117912 IPI00400269 IPI00365296  IPI00403907 | 2.8 | 2 | Semaphorin 5B precursor Hypothetical protein Similar to sema domain seven thrombospondin repeats (type 1 and type 1-like) transmembrane domain (TM) and short cytoplasmic domain (semaphorin) 5B Sema domain seven thrombospondin repeats (type 1 and type 1-like) transmembrane domain (TM) and short cytoplasmic domain | unknown |
| 0.63 | IPI00399886 | 4.8 | 2 | Separin | unknown |
| 1 | IPI00114945 | 33 | 7 | Septin 2 | mt |
| 1 | IPI00204899 IPI00224626 IPI00192454 | 39.2 | 10 | Septin 7 Cell division cycle 10 homolog Ensembl_locations(Chr-bp):8-24924872 | mt |
| 1 | IPI00112935 | 61.1 | 47 | Serine hydroxymethyltransferase | mt |
| 1 | IPI00275992 | 32.3 | 19 | Serine protease HTRA2 mitochondrial precursor | mt |
| 1 | IPI00129351 | 4.2 | 3 | Serine\threonine kinase Haspin | nuleus |
| 1 | IPI00130185 | 32.7 | 9 | Serine\threonine protein phosphatase type 1 alpha | unknown |
| 1 | IPI00225307 IPI00115368 | 15.9 | 5 | Seryl-aminoacyl-tRNA synthetase 1 Seryl-tRNA synthetase | mt |
| 1 | IPI00205157 | 59.9 | 15 | Short chain 3-hydroxyacyl-coa dehydrogenase mitochondrial precursor | mt |
| 1 | IPI00121105 | 59.9 | 14 | Short chain 3-hydroxyacyl-coa dehydrogenase mitochondrial precursor | mt |
| 1 | IPI00115454 | 54.3 | 23 | Sideroflexin 1 | mt |
| 1 | IPI00213735 | 50.9 | 19 | Sideroflexin 1 | mt |
| 1 | IPI00127069 | 13.7 | 3 | Sideroflexin 2 | mt |
| 1 | IPI00404591 IPI00409909 | 14.8 | 4 | Signal recognition particle 54 kda Similar to signal recognition particle 54 kda | mt |
| 1 | IPI00316133 | 6 | 2 | Signal recognition particle 72 | mt |
| 1 | IPI00111271 | 49.4 | 15 | Signal recognition particle receptor beta subunit | ER |
| 0.99 | IPI00366405 IPI00122015 IPI00394248 | 10.5 | 2 | Similar to 0910001A06Rik protein Similar to hypothetical protein dkfzp566a1524 Ensembl_locations(Chr-bp):7-101185384 | mt |
| 1 | IPI00169472 | 53.1 | 28 | Similar to 10-formyltetrahydrofolate dehydrogenase | unknown |
| 0.99 | IPI00269029 | 7.2 | 2 | Similar to 130 kda golgi-localized phosphoprotein | mt |
| 0.93 | IPI00369882 | 4.6 | 3 | Similar to 1-beta dynein | unknown |
| 1 | IPI00169958 | 20.1 | 9 | Similar to 2 3-oxidosqualene: lanosterol cyclase | unknown |
| 1 | IPI00192903 IPI00170357 | 8 | 7 | Similar to 2310036I02Rik protein Similar to AFG3 atpase family gene 3-like 2 | mt |
| 1 | IPI00370382 | 13.6 | 3 | Similar to 26S proteasome non-atpase regulatory subunit 11 (26S proteasome regulatory subunit S9) (26S proteasome regulatory subunit p44.5) | unknown |
| 1 | IPI00362656 IPI00229286 | 7.8 | 2 | Similar to 2700029M09Rik protein Similar to hypothetical protein FLJ20534 | microsome |
| 1 | IPI00357889 | 27.6 | 4 | Similar to 28S ribosomal protein S9 mitochondrial precursor (MRP-S9) | unknown |
| 1 | IPI00365904 | 19.2 | 6 | Similar to 2900070E19Rik protein | unknown |
| 1 | IPI00154047 | 31.7 | 14 | Similar to 3-hydroxyisobutyryl-coenzyme A hydrolase | mt |
| 0.98 | IPI00341516 | 9.8 | 2 | Similar to 3-phosphoglycerate dehydrogenase | unknown |
| 1 | IPI00277923 | 18.4 | 2 | Similar to 3-phosphoglycerate dehydrogenase | mt |
| 1 | IPI00367101 | 15.9 | 4 | Similar to 40S RIBOSOMAL PROTEIN S19 | mt |
| 1 | IPI00134385 IPI00137735 (IPI00379589) (IPI00215184) IPI00269895 IPI00403614 (IPI00371033) (IPI00115992) | 16.1 | 3 | Similar to 40S ribosomal protein S25 40S ribosomal protein S25  Ensembl_locations(Chr-bp):12-105883929 Ensembl_locations(Chr-bp):4-89779330 | unknown |
| 0.97 | IPI00345554 | 31.1 | 2 | Similar to 40S ribosomal protein S7 (S8) | mt |
| 1 | IPI00373197 IPI00351206 | 33.2 | 6 | Similar to 4631434O19Rik protein Progesterone membrane binding protein | membrane |
| 1 | IPI00365301 | 32.5 | 8 | Similar to 5730438N18Rik protein | mt |
| 1 | IPI00108194 | 15 | 3 | Similar to 5OT-EST protein | mt |
| 1 | IPI00340135 | 14.1 | 4 | Similar to 60S ribosomal protein L15 | mt |
| 1 | IPI00368150 | 23.3 | 2 | Similar to 60S ribosomal protein L21 | mt |
| 1 | IPI00367934 (IPI00409333) (IPI00364185)  (IPI00381955)  IPI00203523 IPI00112112 (IPI00162953) | 37.8 | 7 | Similar to 60S ribosomal protein l23a    60S ribosomal protein l23a Ensembl_locations(Chr-bp):1-53342918 | unknown |
| 1 | IPI00358844 IPI00283931 (IPI00379374) IPI00200552 IPI00125389 (IPI00132460) | 11.8 | 6 | Similar to 60S ribosomal protein L26 Ensembl_locations(Chr-bp):2-14629147  60S ribosomal protein L26 Ensembl_locations(Chr-bp):16-17316518 | mt |
| 0.93 | IPI00346530 | 11.3 | 2 | Similar to 60S RIBOSOMAL PROTEIN L3 (L4) | unknown |
| 1 | IPI00360681 | 30.2 | 10 | Similar to 60S ribosomal protein L36 | mt |
| 1 | IPI00368250 (IPI00215208) IPI00137787 | 26.5 | 7 | Similar to 60S ribosomal protein L8  Ribosomal protein L8 | unknown |
| 1 | IPI00370636 | 14.1 | 7 | Similar to 60S ribosomal protein L9 mitochondrial precursor (l9mt) | mt |
| 0.97 | IPI00365314 | 7.3 | 3 | Similar to a disintegrin-like and metalloprotease with thrombospondin type 1 motifs 9B | unknown |
| 0.72 | IPI00371328 | 2.6 | 2 | Similar to abnormal spindle | unknown |
| 1 | IPI00154054 | 41.7 | 31 | Similar to acetyl-Co A acetyltransferase 1 mitochondrial | mt |
| 1 | IPI00337024 IPI00226430 | 41.8 | 15 | Similar to acetyl-coenzyme A acyltransferase 2 3-ketoacyl-coa thiolase | unknown |
| 1 | IPI00194087 IPI00114593 | 34.5 | 9 | Similar to actin alpha cardiac Actin alpha cardiac | unknown |
| 1 | IPI00348049 | 19 | 4 | Similar to actin gamma cytoplasmic | cytoplasm |
| 0.76 | IPI00372040 IPI00138691 | 13.1 | 2 | Similar to actin related protein 2\3 complex subunit 4 ARP2\3 complex 20 kda subunit | unknown |
| 1 | IPI00373752 IPI00399467 IPI00381295 | 5.3 | 4 | Similar to actin-binding protein homolog ABP-278 Ensembl_locations(Chr-bp):14-3103877 Filamin B | mt |
| 1 | IPI00363738 | 57.1 | 5 | Similar to Adenine phosphoribosyltransferase (APRT) | unknown |
| 1 | IPI00139168 | 5.8 | 5 | Similar to ADP-ribosyltransferase | mt |
| 0.95 | IPI00360810 | 3.8 | 2 | Similar to afadin | mt |
| 1 | IPI00358154 (IPI00393033) | 3.1 | 5 | Similar to A-kinase anchor protein 9 isoform 2 | unknown |
| 0.97 | IPI00371719 | 47.8 | 2 | Similar to alpha tubulin | unknown |
| 0.71 | IPI00367109 | 7 | 3 | Similar to alpha-3 type IV collagen | unknown |
| 1 | IPI00370401 IPI00377350 IPI00114407 IPI00311103 (IPI00122339) | 6.7 | 2 | Similar to ALY Splice isoform 2 of Q9JJW6 RNA and export factor binding protein 2 Splice isoform 1 of O08583 THO complex subunit 4 Splice isoform 1 of Q9JJW6 RNA and export factor binding protein 2 | unknown |
| 0.75 | IPI00378753 | 5 | 2 | Similar to aminopeptidase N | mt |
| 1 | IPI00379784 | 38.3 | 6 | Similar to antigen LEC-A | unknown |
| 0.99 | IPI00225176 | 9.9 | 4 | Similar to APE2 protein | mt |
| 1 | IPI00372407 IPI00170307 | 17 | 2 | Similar to apoa-I binding protein Apoa-I binding protein | mt |
| 0.72 | IPI00373651 IPI00135033 | 9.2 | 3 | Similar to apoptosis inhibitor 5 Apoptosis inhibitory protein 5 | unknown |
| 0.69 | IPI00364908 | 3.6 | 2 | Similar to archvillin | unknown |
| 1 | IPI00371043 (IPI00404019)  IPI00110350 | 32.7 | 8 | Similar to arginine-rich mutated in early stage tumors  ARMET protein precursor | unknown |
| 1 | IPI00365499 | 9.7 | 5 | Similar to ARL6IP2 | unknown |
| 1 | IPI00192755 | 28.4 | 3 | Similar to ARP2\3 complex 21 kda subunit (P21-ARC) (Actin-related protein 2\3 complex subunit 3) | unknown |
| 1 | IPI00371286 IPI00120031 | 31.9 | 8 | Similar to arsenic resistance atpase Arsenical pump-driving atpase | ER |
| 1 | IPI00367331 | 22.6 | 7 | Similar to arylacetamide deacetylase (esterase) | ER |
| 0.91 | IPI00359640 | 2.5 | 4 | Similar to AT motif-binding factor | unknown |
| 0.96 | IPI00364993 | 3.2 | 4 | Similar to ATP binding cassette gene sub-family A (ABC1) member 13 | unknown |
| 1 | IPI00339831 | 24.7 | 10 | Similar to ATP synthase H+ transporting mitochondrial F1 complex O subunit | cytoplasm |
| 1 | IPI00369531 IPI00336348 | 7.7 | 2 | Similar to ATP synthase mitochondrial F1 complex assembly factor 2 Hypothetical protein | mt |
| 1 | IPI00368071 IPI00407692 (IPI00373076) | 6.8 | 3 | Similar to atpase H+ transporting V1 subunit A isoform 1 Atpase H+ transporting lysosomal 70kd V1 subunit A isoform 1 | mt |
| 1 | IPI00199600 IPI00133163 | 9.3 | 2 | Similar to atpase H+ transporting V1 subunit G isoform 1 Vacuolar ATP synthase subunit G 1 | nuleus |
| 1 | IPI00358295 | 18.3 | 7 | Similar to ATP-binding cassette sub-family B member 7 mitochondrial precursor (ATP-binding cassette transporter 7) (ABC transporter 7 protein) | mt |
| 1 | IPI00193816  IPI00322869 | 8 | 4 | Similar to ATP-binding cassette sub-family E member 1 (RNAse L inhibitor) (Ribonuclease 4 inhibitor) (RNS4I) (huhp68) ATP-binding cassette sub-family E member 1 | mt |
| 1 | IPI00365343 IPI00120290 | 32.3 | 14 | Similar to ATP-dependent zinc metalloprotease AFG3-like protein 1 | mt |
| 1 | IPI00130304 | 37.1 | 6 | Similar to BCL2-associated athanogene 2 | mt |
| 1 | IPI00213929 | 28.2 | 10 | Similar to BCS1-like | mt |
| 1 | IPI00209037 IPI00133948 | 40 | 7 | Similar to binding protein FK506-binding protein 2 precursor | unknown |
| 1 | IPI00342938 | 17.6 | 4 | Similar to bk1191b2.3.1 (PUTATIVE novel Acyl Transferase Similar to C. Elegans C50D2.7) (variant 1)) | mt |
| 0.99 | IPI00120549 | 3.5 | 4 | Similar to bone specific CMF608 | unknown |
| 1 | IPI00122442 | 13 | 7 | Similar to branched chain aminotransferase 2 mitochondrial | mt |
| 1 | IPI00362153 IPI00191039 | 3.1 | 2 | Similar to bullous pemphigoid antigen 1-b Ensembl_locations(Chr-bp):9-32714647 | mt |
| 0.65 | IPI00367893 | 14.6 | 2 | Similar to C230063O06 protein | unknown |
| 1 | IPI00365582 | 3.3 | 3 | Similar to CAD protein | unknown |
| 1 | IPI00368936 | 23.9 | 10 | Similar to calcium-binding transporter | mt |
| 1 | IPI00379571 | 14.5 | 3 | Similar to calmodulin - rabbit (tentative sequence) | mt |
| 1 | IPI00353523 | 36.1 | 2 | Similar to candidate tumor suppressor protein | mt |
| 1 | IPI00368907 | 42 | 8 | Similar to Cappa1 protein | unknown |
| 1 | IPI00365283 IPI00406800 | 17.6 | 3 | Similar to capping protein beta subunit, isoform 2 Splice isoform 1 of P47757 F-actin capping protein beta subunit | unknown |
| 1 | IPI00367861 IPI00133828 | 5.1 | 2 | Similar to carbohydrate (chondroitin) synthase 1 Similar to mkiaa0990 protein | mt |
| 1 | IPI00344090 | 21.5 | 3 | Similar to CDNA FLJ10581 FIS | membrane |
| 0.6 | IPI00351472 | 6.7 | 2 | Similar to CDNA sequence BC018371 | unknown |
| 1 | IPI00116297 | 10.4 | 3 | Similar to CDP-diacylglycerol synthase | unknown |
| 1 | IPI00162949 | 6.1 | 2 | Similar to CDP-diacylglycerol--inositol 3-phosphatidyltransferase | mt |
| 0.93 | IPI00380142 | 14.9 | 2 | Similar to CG016 | unknown |
| 0.67 | IPI00351563 | 6 | 2 | Similar to CG32384-PA | unknown |
| 1 | IPI00373135 IPI00403703 IPI00223856 | 3.6 | 2 | Similar to CG5554-PA 2810417D04Rik protein Thioredoxin-like protein KIAA1162 homolog precursor | mt |
| 1 | IPI00381212 | 9.2 | 3 | Similar to CG8014-PA | unknown |
| 1 | IPI00355250 | 5.9 | 4 | Similar to CG9318-PA | mt |
| 1 | IPI00358470 IPI00133212 | 10.9 | 6 | Similar to CGI-141 protein Hypothetical UPF0198 protein CGI-141 homolog | unknown |
| 1 | IPI00368291 | 30.1 | 8 | Similar to CGI-143 protein | unknown |
| 1 | IPI00153266 | 36.8 | 19 | Similar to CGI-49 protein | unknown |
| 1 | IPI00221564 | 27.8 | 5 | Similar to CGI-92 protein | mt |
| 1 | IPI00367532 | 21.9 | 4 | Similar to chaperonin 60 | mt |
| 0.69 | IPI00366217 | 4.5 | 2 | Similar to chromosome 11 open reading frame 9 | unknown |
| 1 | IPI00284859 | 12.1 | 2 | Similar to chromosome 14 open reading frame 127 | mt |
| 1 | IPI00359856 IPI00378237 | 1.9 | 2 | Similar to chromosome 16 open reading frame 7 Hypothetical vacuolar sorting protein 9 | unknown |
| 0.79 | IPI00361597 | 2.9 | 3 | Similar to chromosome 17 open reading frame 27 | unknown |
| 1 | IPI00221540 | 39.4 | 12 | Similar to chromosome 8 open reading frame 2 | unknown |
| 1 | IPI00358720 (IPI00379305) | 33.8 | 4 | Similar to chromosome 9 open reading frame 19 | unknown |
| 1 | IPI00121627 | 9 | 3 | Similar to cleft lip and palate associated transmembrane protein 1 | unknown |
| 1 | IPI00373718 | 34.2 | 19 | Similar to clpp protease | mt |
| 1 | IPI00364504 | 4.4 | 3 | Similar to coat protein gamma-cop | unknown |
| 0.91 | IPI00125538 | 5 | 2 | Similar to Cohen syndrome 1 protein isoform 1 | unknown |
| 1 | IPI00364377 | 12.9 | 2 | Similar to coiled-coil protein | mt |
| 0.99 | IPI00357842 | 19.5 | 3 | Similar to collagen alpha 2(VI) chain precursor long splice form - human | unknown |
| 0.6 | IPI00371784 | 5.3 | 2 | Similar to colon cancer antigen NY-CO-45 | unknown |
| 1 | IPI00193349 IPI00131871 | 13.3 | 2 | Similar to COP9 complex subunit 4 COP9 complex subunit 4 | mt |
| 0.94 | IPI00191745 | 24.2 | 2 | Similar to coproporphyrinogen oxidase | mt |
| 1 | IPI00229441 | 12.4 | 3 | Similar to COX10 homolog cytochrome c oxidase assembly protein heme A: farnesyltransferase | mt |
| 1 | IPI00196099 IPI00119413 IPI00389600 | 30.1 | 2 | Similar to COX11 homolog RIKEN cDNA 2010004I09 Ensembl_locations(Chr-bp):10-79054094 | unknown |
| 1 | IPI00222203 IPI00123527 IPI00267596 | 25 | 9 | Similar to COX15 homolog Similar to COX15 homolog cytochrome c oxidase assembly protein Hypothetical protein | mt |
| 1 | IPI00365505 | 47.8 | 6 | Similar to Cox7a2l protein | unknown |
| 1 | IPI00278602 | 7.9 | 2 | Similar to Cyclic-AMP-dependent transcription factor ATF-6 alpha (Activating transcription factor 6 alpha) (ATF6-alpha) | mt |
| 0.96 | IPI00381416 | 3.5 | 2 | Similar to cyclin-dependent kinase-like 5 | unknown |
| 1 | IPI00362291 | 37.7 | 2 | Similar to cytochrome c oxidase polypeptide via mitochondrial precursor | membrane |
| 1 | IPI00366416 | 39.3 | 36 | Similar to cytochrome c-1 | mt |
| 1 | IPI00222582 | 14.9 | 5 | Similar to cytochrome P450 monooxygenase | mt |
| 1 | IPI00387292 IPI00127172 | 14.9 | 9 | Similar to DEAD\H (Asp-Glu-Ala-Asp\His) box polypeptide 1 Hypothetical protein | unknown |
| 0.61 | IPI00353581 | 7.6 | 2 | Similar to death effector filament-forming Ced-4-like apoptosis protein isoform 3 | unknown |
| 0.92 | IPI00371785 | 7.5 | 2 | Similar to death receptor 6 | unknown |
| 0.94 | IPI00359210 | 7 | 2 | Similar to dj366n23.3 (NYD-TSPG protein) | unknown |
| 0.65 | IPI00364560 | 3.7 | 2 | Similar to DKFZP564P1916 protein | unknown |
| 0.82 | IPI00359278 | 9.9 | 2 | Similar to DKFZP566K1924 protein | unknown |
| 1 | IPI00364340 IPI00123557 | 9.7 | 4 | Similar to DNA helicase Ruvb-like 2 | mt |
| 1 | IPI00189203 IPI00128346 | 45.4 | 11 | Similar to DNA segment Chr 10 ERATO Doi 214 expressed Uncharacterized hematopoietic stem\progenitor cells protein MDS029 homolog | mt |
| 1 | IPI00378302 IPI00133122 | 30.2 | 5 | Similar to DNA segment Chr 4 Wayne state University 125 expressed DNA segment Chr 4 Wayne state University 125 expressed | mt |
| 0.74 | IPI00360771 | 3.5 | 2 | Similar to DNA segment on chromosome 10 (unique) 170 | unknown |
| 0.99 | IPI00363584 | 2.1 | 4 | Similar to DNA-dependent protein kinase catalytic subunit | unknown |
| 1 | IPI00308717 | 11.5 | 8 | Similar to DNA-directed RNA polymerase | mt |
| 1 | IPI00359930 | 2.6 | 4 | Similar to down-regulated in metastasis | mt |
| 1 | IPI00187434 | 24 | 3 | Similar to dutpase | unknown |
| 1 | IPI00371428 IPI00126083 | 19.7 | 3 | Similar to EH-domain containing 1 EH-domain containing protein 1 | mt |
| 0.85 | IPI00366815 | 8.2 | 4 | Similar to Eif4g2 protein | unknown |
| 1 | IPI00211929 IPI00365506  IPI00126006 | 2.4 | 2 | Similar to Eif4g2 protein Similar to Eukaryotic translation initiation factor 4 gamma 2 (eif-4-gamma 2) (eif-4G 2) (eif4g 2) (p97) (Novel APOBEC-1 target 1) (Translation repressor NAT1) Eukaryotic translation initiation factor 4 gamma 2 | mt |
| 1 | IPI00322145 | 24 | 8 | Similar to EIF4GII | unknown |
| 1 | IPI00371508 | 21.7 | 4 | Similar to Elongation factor 1-alpha 1 (EF-1-alpha-1) (Elongation factor 1 A-1) (eef1a-1) (Elongation factor Tu) (EF-Tu) | mt |
| 1 | IPI00367441 | 11.7 | 2 | Similar to Elongation factor 1-alpha 1 (EF-1-alpha-1) (Elongation factor 1 A-1) (eef1a-1) (Elongation factor Tu) (EF-Tu) | mt |
| 0.98 | IPI00352095 | 29.2 | 2 | Similar to Elongation factor 1-alpha 1 (EF-1-alpha-1) (Elongation factor 1 A-1) (eef1a-1) (Elongation factor Tu) (EF-Tu) | nuleus |
| 0.6 | IPI00368268 | 6.3 | 2 | Similar to epidermis-specific serine protease-like protein precursor | unknown |
| 1 | IPI00230108 | 53.9 | 52 | Similar to ER-60 protease | unknown |
| 1 | IPI00130310 | 26.3 | 6 | Similar to Eukaryotic initiation factor 4A-like NUK-34 (DEAD-box protein 48) | unknown |
| 1 | IPI00366398  IPI00132250 | 11.9 | 3 | Similar to Eukaryotic translation initiation factor 3 subunit 6 (eif-3 p48) (eif3e) (Mammary tumor-associated protein INT-6) (Viral integration site protein INT-6) Eukaryotic translation initiation factor 3 subunit 6 | mt |
| 1 | IPI00211216  IPI00402766 (IPI00108125) | 43.1 | 11 | Similar to Eukaryotic translation initiation factor 5A (eif-5A) (eif-4D) (Rev-binding factor) Eukaryotic translation initiation factor 5A | mt |
| 1 | IPI00358404 IPI00312468 | 36.6 | 14 | Similar to eukaryotic translation termination factor 1 Eukaryotic translation termination factor 1 | unknown |
| 1 | IPI00366079 IPI00163011 | 6 | 2 | Similar to expressed sequence AL022641 Thioredoxin domain containing protein 5 precursor | mt |
| 1 | IPI00371146 IPI00224069 | 19.6 | 6 | Similar to expressed sequence AW557704 Similar to endonuclease G like 1 | unknown |
| 1 | IPI00372380 | 8.6 | 3 | Similar to fatty acid transport protein 3 | mt |
| 1 | IPI00364039 | 4 | 3 | Similar to Fc fragment of igg binding protein | mt |
| 0.97 | IPI00361297 | 4.4 | 5 | Similar to fetal Alzheimer antigen isoform 2 | unknown |
| 1 | IPI00379907  IPI00189992 IPI00327212 (IPI00369427) IPI00399483 IPI00310521 (IPI00382031) | 18.6 | 2 | Similar to Finkel-Biskis-Reilly murine sarcoma virus (FBR-musv) ubiquitously expressed (fox derived) Ensembl_locations(Chr-bp):1-157254232 Similar to fusion protein  40S ribosomal protein S30 Monoclonal non-specific suppressor factor beta | cytoplasm |
| 1 | IPI00371718 | 23.6 | 22 | Similar to FLJ00343 protein | ER |
| 1 | IPI00112366 | 45.1 | 8 | Similar to FLJ23469 protein | unknown |
| 0.99 | IPI00122362 | 12.8 | 2 | Similar to for protein disulfide isomerase-related | nuleus |
| 1 | IPI00367557 IPI00136201 IPI00389333 | 27.1 | 2 | Similar to frataxin Frataxin mitochondrial precursor Ensembl_locations(Chr-bp):1-227811261 | mt |
| 1 | IPI00372829 | 9.6 | 3 | Similar to Ga17 protein | mt |
| 0.98 | IPI00364754  IPI00131488 | 8.9 | 2 | Similar to GDP-fucose protein O-fucosyltransferase 1 precursor (Peptide O-fucosyltransferase) (O-fuct-1) GDP-fucose protein O-fucosyltransferase 1 precursor | synaptsome |
| 0.99 | IPI00369229 | 5.4 | 4 | Similar to gem (nuclear organelle) associated protein 5 | unknown |
| 0.9 | IPI00363429 IPI00129535 | 7.2 | 2 | Similar to gene trap locus-13 Nuclear pore complex protein Nup160 | unknown |
| 0.78 | IPI00354218 | 7.6 | 2 | Similar to GLE1-like RNA export mediator | unknown |
| 1 | IPI00387534 IPI00265352 | 16.5 | 8 | Similar to glutamic pyruvate transaminase (Alanine aminotransferase) 2 Weakly Similar to alanine aminotransferase | unknown |
| 0.99 | IPI00373262 | 12.1 | 2 | Similar to glyceraldehyde-3-phosphate dehydrogenase | unknown |
| 1 | IPI00351302 | 15.6 | 5 | Similar to glyceraldehyde-3-phosphate dehydrogenase (phosphorylating) (EC 1.2.1.12) - mouse | unknown |
| 0.99 | IPI00343558 | 16.8 | 3 | Similar to glyceraldehyde-3-phosphate dehydrogenase (phosphorylating) (EC 1.2.1.12) - mouse | unknown |
| 1 | IPI00341308 | 15.1 | 2 | Similar to glyceraldehyde-3-phosphate dehydrogenase (phosphorylating) (EC 1.2.1.12) - mouse | mt |
| 0.86 | IPI00353507 | 15.5 | 2 | Similar to glyceraldehyde-3-phosphate dehydrogenase (phosphorylating) (EC 1.2.1.12) - mouse | unknown |
| 1 | IPI00368095 | 7.5 | 3 | Similar to golgi phosphoprotein 4 | unknown |
| 0.98 | IPI00364707 (IPI00345591) IPI00112835 IPI00274004 | 4.8 | 2 | Similar to gp25l2 protein  2400003B06Rik protein Glycoprotein 25L2 precursor | mt |
| 1 | IPI00364208 | 32.1 | 19 | Similar to G-rich sequence factor-1 (GRSF-1) | unknown |
| 1 | IPI00192736 IPI00263313 | 9.8 | 2 | Similar to GTP-binding protein Developmentally regulated GTP-binding protein 1 | mt |
| 0.97 | IPI00405110 IPI00153425 IPI00371878 IPI00394154 | 9.5 | 2 | Similar to GTT1 Star-related lipid transfer protein 7 Similar to START domain containing 7 Ensembl_locations(Chr-bp):3-114674239 | unknown |
| 1 | IPI00369012 | 22.6 | 3 | Similar to guanine nucleotide-binding protein beta-1 subunit | cytoplasm |
| 1 | IPI00226521 | 17.7 | 2 | Similar to HCDI protein | mt |
| 1 | IPI00361689 | 29.5 | 10 | Similar to Heat shock 60 kd protein 1 | mt |
| 1 | IPI00362245 | 27.5 | 3 | Similar to Heat shock cognate 71 kda protein | unknown |
| 1 | IPI00350780 | 18.7 | 2 | Similar to heat shock protein 60 (liver) | mt |
| 0.96 | IPI00369183 | 23.2 | 2 | Similar to heat shock protein 60 (liver) | unknown |
| 1 | IPI00371348 | 22.1 | 6 | Similar to heat shock protein 65 | mt |
| 1 | IPI00358461 | 37.9 | 3 | Similar to heme-binding protein | unknown |
| 0.84 | IPI00409178 | 5.3 | 2 | Similar to hemicentin | unknown |
| 1 | IPI00367833 | 3.8 | 6 | Similar to Herc2 | unknown |
| 1 | IPI00370207 (IPI00359402) IPI00215244 IPI00124979 | 7 | 2 | Similar to heterogeneous nuclear ribonucleoprotein G - human  Ensembl_locations(Chr-bp):4-41341550 Heterogeneous nuclear ribonucleoprotein G | mt |
| 0.63 | IPI00359647 | 2.8 | 2 | Similar to hexose-6-phosphate dehydrogenase precursor | unknown |
| 1 | IPI00346804 | 26.7 | 2 | Similar to HIRA-interacting protein 5 (mhirip5) | mt |
| 1 | IPI00362846 | 15.4 | 4 | Similar to holocytochrome c-type synthetase | mt |
| 1 | IPI00361820 IPI00322440 | 14 | 2 | Similar to huntingtin interacting protein 2 Ubiquitin-conjugating enzyme E2-25 kda | mt |
| 0.64 | IPI00378581 | 4.9 | 2 | Similar to huntingtin interacting protein B isoform 1 | unknown |
| 0.74 | IPI00215495 IPI00138238 | 17.3 | 2 | Similar to hydrophilic protein Prefoldin subunit 6 | unknown |
| 1 | IPI00115607 | 46.1 | 29 | Similar to hydroxyacyl-coenzyme A dehydrogenase\3-ketoacyl-coenzyme A thiolase\enoyl-coenzyme A hydratase | mt |
| 1 | IPI00214242 | 7.1 | 3 | Similar to hypothetical protein | mt |
| 0.77 | IPI00358808 | 14.9 | 2 | Similar to hypothetical protein | unknown |
| 0.85 | IPI00362288 | 7.8 | 2 | Similar to hypothetical protein | unknown |
| 0.72 | IPI00367664 | 3 | 2 | Similar to hypothetical protein | unknown |
| 0.66 | IPI00360283 | 3.6 | 2 | Similar to hypothetical protein | unknown |
| 0.97 | IPI00120395 | 6 | 2 | Similar to hypothetical protein 5930437N14 | unknown |
| 0.79 | IPI00360576 | 9.6 | 3 | Similar to hypothetical protein A830048P05 | unknown |
| 1 | IPI00153640 | 11.9 | 4 | Similar to hypothetical protein BC004409 | unknown |
| 1 | IPI00353887 | 27.7 | 12 | Similar to hypothetical protein BC010682 | unknown |
| 1 | IPI00362537 | 11.2 | 2 | Similar to hypothetical protein BC011833 | mt |
| 1 | IPI00314204 | 19.8 | 3 | Similar to hypothetical protein clone Telethon(Italy_B41)_Strait02270_FL142 | mt |
| 1 | IPI00190858 | 7 | 3 | Similar to hypothetical protein D16Ertd454e | unknown |
| 0.99 | IPI00365864 | 10.8 | 2 | Similar to hypothetical protein DC50 | unknown |
| 0.83 | IPI00343024 IPI00359196 | 3.4 | 2 | Similar to hypothetical protein dkfzp434i1930 Similar to hypothetical protein | unknown |
| 0.94 | IPI00369668 | 12.7 | 2 | Similar to hypothetical protein dkfzp547c176 | mt |
| 0.62 | IPI00358961 | 15.7 | 2 | Similar to hypothetical protein FLJ10326 | unknown |
| 1 | IPI00165794 | 15.2 | 3 | Similar to hypothetical protein FLJ10407 | unknown |
| 0.97 | IPI00360643 | 11.1 | 2 | Similar to hypothetical protein FLJ11127 | unknown |
| 1 | IPI00115475 | 9.3 | 5 | Similar to hypothetical protein FLJ11200 | unknown |
| 1 | IPI00362402 | 13.1 | 3 | Similar to hypothetical protein FLJ11342 | unknown |
| 1 | IPI00362413 | 23.3 | 18 | Similar to hypothetical protein FLJ12442 | mt |
| 1 | IPI00128488 | 36.2 | 16 | Similar to hypothetical protein FLJ12442 | unknown |
| 1 | IPI00130331 | 10.3 | 3 | Similar to hypothetical protein FLJ12618 | unknown |
| 1 | IPI00312174 | 37.5 | 12 | Similar to hypothetical protein FLJ14038 | unknown |
| 1 | IPI00371704 | 33.8 | 9 | Similar to hypothetical protein FLJ20445 | mt |
| 1 | IPI00229776 IPI00387302 IPI00387520 | 11.8 | 4 | Similar to hypothetical protein FLJ20627 CDNA: FLJ22827 FIS Similar to hypothetical protein FLJ20627 | unknown |
| 0.83 | IPI00169888 | 12.6 | 2 | Similar to hypothetical protein FLJ21128 | unknown |
| 0.6 | IPI00366810 | 9.7 | 2 | Similar to hypothetical protein FLJ25348 | unknown |
| 0.95 | IPI00358550 | 6.8 | 2 | Similar to hypothetical protein FLJ25801 | unknown |
| 0.84 | IPI00357650 | 8.1 | 3 | Similar to hypothetical protein FLJ32779 | unknown |
| 0.63 | IPI00362817 | 5.2 | 2 | Similar to hypothetical protein FLJ39502 | unknown |
| 1 | IPI00224237 | 6.2 | 2 | Similar to hypothetical protein FLJ90492 | mt |
| 1 | IPI00360426 IPI00283630 IPI00319131 IPI00367228 (IPI00371516) | 10.4 | 2 | Similar to Hypothetical protein KIAA0427 PXF protein Peroxisomal farnesylated protein Similar to PXF protein | mt |
| 1 | IPI00360121 | 1.2 | 3 | Similar to hypothetical protein MGC19022 | unknown |
| 1 | IPI00221782 | 12.8 | 4 | Similar to hypothetical protein MGC24180 | mt |
| 0.9 | IPI00369050 | 3.9 | 2 | Similar to hypothetical protein MGC38936 | unknown |
| 0.75 | IPI00372410 | 5.6 | 3 | Similar to hypothetical protein MGC46719 | unknown |
| 1 | IPI00392594 IPI00226387 | 13.9 | 5 | Similar to hypothetical protein MGC5352 Hypothetical phosphoglycerate mutase family containing protein | unknown |
| 1 | IPI00123281 | 41.4 | 24 | Similar to hypothetical protein PRO1855 | unknown |
| 1 | IPI00262198 | 24.5 | 3 | Similar to hypothetical protein Similar to CG7943 | unknown |
| 0.97 | IPI00310713 IPI00368705 | 20.9 | 2 | Similar to hypothetical protein Similar to RIKEN cDNA 2610030N08 | unknown |
| 0.72 | IPI00366041 | 8.9 | 2 | Similar to I beta-1,6-N-acetylglucosaminyltransferase isoform C | unknown |
| 1 | IPI00226771 | 18.6 | 3 | Similar to I3 binding protein | mt |
| 1 | IPI00379787 | 35.3 | 10 | Similar to Ig E binding factor 10.2 | mt |
| 1 | IPI00365970 IPI00225209 IPI00393386 | 4.8 | 2 | Similar to ilvb (bacterial acetolactate synthase)-like isoform 1 Ilvb Ensembl_locations(Chr-bp):7-12593339 | mt |
| 0.63 | IPI00368909 | 4.6 | 2 | Similar to immunoglobulin superfamily member 2 | unknown |
| 1 | IPI00364895 | 35.2 | 6 | Similar to inner membrane protein mitochondrial (mitofilin) | mt |
| 0.99 | IPI00388742 | 16.6 | 2 | Similar to interferon kappa precursor | unknown |
| 1 | IPI00215473 IPI00394257 IPI00118834 | 13.6 | 5 | Similar to KE4 Ensembl_locations(Chr-bp):20-4961872 Zinc transporter SLC39A7 | unknown |
| 1 | IPI00222228 | 13.4 | 4 | Similar to keratin | unknown |
| 0.84 | IPI00370194 | 8.2 | 3 | Similar to keratin 5b | unknown |
| 1 | IPI00348328 | 12.5 | 3 | Similar to keratin 5b | unknown |
| 1 | IPI00124499 | 5.7 | 2 | Similar to keratin 6A | mt |
| 1 | IPI00361757 | 8.4 | 2 | Similar to Keratin type II cytoskeletal 4 (Cytokeratin 4) (Cytoskeletal 57 kda keratin) | mt |
| 1 | IPI00322295 IPI00222307 | 10.1 | 5 | Similar to KIAA0095 gene product DEAD EYE homolog | unknown |
| 0.69 | IPI00350413 | 4.7 | 3 | Similar to KIAA0453 protein | unknown |
| 0.65 | IPI00371105 | 3.3 | 3 | Similar to KIAA0540 protein | unknown |
| 0.76 | IPI00321421 | 12.6 | 2 | Similar to KIAA0599 protein | unknown |
| 0.94 | IPI00364802 | 7.5 | 4 | Similar to KIAA0980 protein | unknown |
| 0.96 | IPI00363648 | 3.6 | 2 | Similar to KIAA1025 protein | unknown |
| 0.87 | IPI00362594 | 2.5 | 2 | Similar to KIAA1202 protein | unknown |
| 1 | IPI00342603 | 29.5 | 14 | Similar to KIAA1290 protein | unknown |
| 1 | IPI00368082 | 4.1 | 3 | Similar to KIAA1409 protein | mt |
| 0.76 | IPI00129924 | 3.3 | 2 | Similar to KIAA1462 protein | unknown |
| 0.73 | IPI00365646 | 3.6 | 2 | Similar to KIAA1509 protein | unknown |
| 0.62 | IPI00355962 | 1.9 | 3 | Similar to KIAA1689 protein | unknown |
| 1 | IPI00361967 | 4.7 | 3 | Similar to KIAA1771 protein | unknown |
| 0.72 | IPI00380922 | 3.4 | 2 | Similar to KIAA1783 protein | unknown |
| 0.97 | IPI00358294 | 5.1 | 3 | Similar to KIAA2022 protein | unknown |
| 0.63 | IPI00358756 | 5.6 | 3 | Similar to Laminin alpha-3 chain precursor (Nicein alpha subunit) | unknown |
| 0.89 | IPI00365542 | 5.7 | 2 | Similar to laminin B1 | unknown |
| 1 | IPI00360075 | 44 | 81 | Similar to leucine rich protein mlrp130 | unknown |
| 1 | IPI00364777 | 35.3 | 25 | Similar to leucine zipper-EF-hand containing transmembrane protein 1 | unknown |
| 1 | IPI00331038 | 11.6 | 3 | Similar to likely ortholog of mouse ADP-ribosylation-like factor 6 interacting protein 2 | mt |
| 0.99 | IPI00371306 | 4.1 | 2 | Similar to LIM domains containing protein 1 | mt |
| 1 | IPI00123379 | 6.2 | 4 | Similar to lipoprotein-binding protein | unknown |
| 0.91 | IPI00351624 | 2.9 | 2 | Similar to low voltage-activated T-type calcium channel alpha-1 subunit (CA | unknown |
| 1 | IPI00225747 | 23.2 | 5 | Similar to M5-14 protein | mt |
| 1 | IPI00378999 IPI00409501 IPI00122073 (IPI00381304) | 21.6 | 2 | Similar to macrophage migration inhibitory factor Ensembl_locations(Chr-bp):7-23900019 Ensembl_locations(Chr-bp):9-15044006 | mt |
| 0.92 | IPI00359003 | 2.1 | 3 | Similar to macrophin 1 isoform 4 | unknown |
| 1 | IPI00114866 | 19.7 | 7 | Similar to malonyl-coa decarboxylase | mt |
| 0.64 | IPI00359652 | 8.9 | 2 | Similar to mannose receptor precursor-like isoform 4 | unknown |
| 1 | IPI00128450 | 26.1 | 28 | Similar to membrane bound C2 domain containing protein | unknown |
| 0.78 | IPI00365337 | 3.9 | 2 | Similar to membrane glycoprotein | unknown |
| 0.95 | IPI00364354 | 7.7 | 4 | Similar to membrane-bound transferrin-like protein p97 | unknown |
| 1 | IPI00153633 | 6.8 | 3 | Similar to meningioma expressed antigen 6 | mt |
| 1 | IPI00347815 | 22.3 | 3 | Similar to meningioma-expressed antigen 6\11 (MEA6) (MEA11) | unknown |
| 1 | IPI00190375 IPI00405351 (IPI00349285) | 18.3 | 4 | Similar to Mesoderm development candidate 2 Mesoderm development candiate 2 | unknown |
| 0.96 | IPI00368621 IPI00274717 | 6.7 | 2 | Similar to metaxin 1 Ensembl_locations(Chr-bp):6-128858812 | unknown |
| 1 | IPI00372898 | 33.3 | 17 | Similar to methylcrotonoyl-Coenzyme A carboxylase 2 (beta) | mt |
| 1 | IPI00365105 | 58.9 | 35 | Similar to methylenetetrahydrofolate dehydrogenase (NAD) (EC 1.5.1.15) \ methenyltetrahydrofolate cyclohydrolase (EC 3.5.4.9) precursor - mouse | unknown |
| 0.82 | IPI00372549 | 6.4 | 2 | Similar to MHC class I cell surface glycoprotein | unknown |
| 1 | IPI00372009 | 17.9 | 23 | Similar to Microtubule-associated protein 1B (MAP 1B) (Neuraxin) | unknown |
| 1 | IPI00128285 | 19 | 4 | Similar to MIPP65 protein | unknown |
| 1 | IPI00222514 | 33.7 | 13 | Similar to mitochondrial 28S ribosomal protein S27 | mt |
| 1 | IPI00222538 | 22.5 | 8 | Similar to mitochondrial 28S ribosomal protein S28 | mt |
| 0.99 | IPI00214903 | 23.4 | 2 | Similar to Mitochondrial 28S ribosomal protein S30 (s30mt) (MRP-S30) | mt |
| 1 | IPI00116074 | 47.8 | 8 | Similar to mitochondrial aconitase | mt |
| 1 | IPI00366434 | 39.6 | 8 | Similar to Mitochondrial import receptor subunit TOM22 homolog (Translocase of outer membrane 22 kda subunit homolog) | mt |
| 1 | IPI00362568 | 17.4 | 2 | Similar to mitochondrial inner membrane translocase component Tim17b | mt |
| 1 | IPI00403180 (IPI00378474) | 29.2 | 16 | Similar to mitochondrial isoleucine tRNA synthetase | mt |
| 1 | IPI00361793 | 39.3 | 11 | Similar to mitochondrial ribosomal protein L1 (l1mt) | mt |
| 1 | IPI00203438 | 26.7 | 6 | Similar to mitochondrial ribosomal protein L10 | mt |
| 1 | IPI00190153 IPI00109740 | 14.4 | 6 | Similar to mitochondrial ribosomal protein L2 Ribosomal protein mitochondrial L14 | mt |
| 1 | IPI00365066 | 29.1 | 6 | Similar to mitochondrial ribosomal protein L37 | mt |
| 1 | IPI00361536 | 14.2 | 4 | Similar to mitochondrial ribosomal protein L38 | mt |
| 1 | IPI00198811 (IPI00206483) (IPI00378520) | 25.4 | 4 | Similar to mitochondrial ribosomal protein L41 | membrane |
| 1 | IPI00371171 | 18 | 5 | Similar to mitochondrial ribosomal protein L48 | mt |
| 1 | IPI00348248 | 38.1 | 10 | Similar to mitochondrial ribosomal protein S10 | mt |
| 1 | IPI00362092 IPI00132147 | 34.1 | 6 | Similar to mitochondrial ribosomal protein S16 28S ribosomal protein S16 mitochondrial precursor | mt |
| 1 | IPI00191107 IPI00391281 | 29.9 | 3 | Similar to mitochondrial ribosomal protein S21 Ensembl_locations(Chr-bp):2-190609002 | mt |
| 1 | IPI00364745 | 22.5 | 7 | Similar to mitochondrial ribosomal protein S35 | mt |
| 1 | IPI00343017 | 10.2 | 6 | Similar to mkiaa0225 protein | unknown |
| 0.94 | IPI00215569 IPI00408228 IPI00117803 IPI00214329 | 9.2 | 2 | Similar to mkiaa0289 protein Hypothetical protein Astrotactin 1 Ensembl_locations(Chr-bp):13-73665039 | unknown |
| 0.92 | IPI00330232 | 4.8 | 3 | Similar to mkiaa0316 protein | unknown |
| 0.62 | IPI00372846 IPI00229645 | 2.6 | 2 | Similar to mkiaa0376 protein MKIAA0376 protein | unknown |
| 0.64 | IPI00371673 | 3.5 | 2 | Similar to mkiaa0595 protein | unknown |
| 1 | IPI00362620 | 29.8 | 4 | Similar to mkiaa0719 protein | unknown |
| 0.7 | IPI00370974 | 4.9 | 2 | Similar to mkiaa1136 protein | unknown |
| 0.9 | IPI00359317 IPI00222107 | 7.4 | 3 | Similar to mkiaa1294 protein RIKEN 2700017I06 | unknown |
| 1 | IPI00169711 | 15.2 | 7 | Similar to monoamine oxidase A | mt |
| 1 | IPI00193245 | 24.5 | 3 | Similar to Mrpl50 protein | unknown |
| 1 | IPI00371766 | 18 | 7 | Similar to Mut protein | unknown |
| 1 | IPI00365938 | 2.5 | 3 | Similar to myosin-like protein | mt |
| 1 | IPI00282911 IPI00124221 | 13.8 | 3 | Similar to Na/K-atpase beta 3 subunit Sodium\potassium-transporting atpase beta-3 chain | membrane |
| 1 | IPI00177100 (IPI00128023) | 40.8 | 20 | Similar to NADH dehydrogenase | unknown |
| 1 | IPI00130460 | 27.6 | 9 | Similar to NADH dehydrogenase | mt |
| 1 | IPI00358441 | 42.2 | 18 | Similar to NADH dehydrogenase (ubiquinone) 1 alpha subcomplex 9 | mt |
| 1 | IPI00372179 | 12.7 | 4 | Similar to NADH dehydrogenase (ubiquinone) 1 beta subcomplex 5 | mt |
| 1 | IPI00364850 (IPI00341322) | 14.1 | 2 | Similar to NADH dehydrogenase | mt |
| 1 | IPI00117300 IPI00378672 | 22.6 | 3 | Similar to NADH dehydrogenase Similar to NADH dehydrogenase (ubiquinone) Fe-S protein 5 | mt |
| 0.82 | IPI00372072 | 1.7 | 2 | Similar to Nebulin | unknown |
| 1 | IPI00366877 | 9.4 | 5 | Similar to neuroblastoma-amplified protein | mt |
| 0.98 | IPI00372556 IPI00130339 | 7.2 | 2 | Similar to NG26 Protein BAT5 | mt |
| 0.75 | IPI00370029 | 3.7 | 2 | Similar to NG28 | mt |
| 1 | IPI00364830 IPI00321895 IPI00331145 | 10.1 | 2 | Similar to non-canonical ubquitin conjugating enzyme 1 0710008M05Rik protein Non-Canonical ubiquitin conjugating enzyme 1 | unknown |
| 0.85 | IPI00352916 | 16.9 | 2 | Similar to nuclear pore-targeting complex component of 58 kda | unknown |
| 1 | IPI00366752 | 23.8 | 24 | Similar to nuclear transplantation upregulated protein 1 | unknown |
| 0.94 | IPI00311453 | 7.2 | 3 | Similar to nucleolar protein 1 | unknown |
| 0.98 | IPI00368476 (IPI00136051) | 5.2 | 2 | Similar to nucleoporin 98kd isoform 1 | unknown |
| 0.98 | IPI00372183 IPI00265371 | 7.2 | 2 | Similar to osmotic stress protein RIKEN cDNA 1700034J06 | unknown |
| 0.83 | IPI00366554 | 4.9 | 3 | Similar to oxygen-regulated photoreceptor protein 1 | unknown |
| 1 | IPI00369693 | 14.1 | 9 | Similar to oxysterol-binding protein-like protein 8 | unknown |
| 1 | IPI00355970 | 10.5 | 3 | Similar to P38IP protein | cytoplasm |
| 1 | IPI00358443 IPI00230139 | 13.3 | 2 | Similar to p59 immunophilin FK506 binding protein 4 | mt |
| 0.99 | IPI00362021 | 4.2 | 2 | Similar to parvin gamma | mt |
| 0.81 | IPI00367751 | 2.6 | 3 | Similar to pecanex 1 | unknown |
| 1 | IPI00208203 | 17.3 | 5 | Similar to Peci protein | mt |
| 1 | IPI00343301 | 35.6 | 4 | Similar to Peptidyl-prolyl cis-trans isomerase A (ppiase) (Rotamase) (Cyclophilin A) (Cyclosporin A-binding protein) (SP18) | mt |
| 0.99 | IPI00340441 | 32.3 | 3 | Similar to Peptidyl-prolyl cis-trans isomerase A (ppiase) (Rotamase) (Cyclophilin A) (Cyclosporin A-binding protein) (SP18) | unknown |
| 0.63 | IPI00362536 IPI00391557 | 12.6 | 2 | Similar to Peptidyl-prolyl cis-trans isomerase A (ppiase) (Rotamase) (Cyclophilin A) (Cyclosporin A-binding protein) (SP18) Ensembl_locations(Chr-bp):X-21940572 | unknown |
| 1 | IPI00201417 IPI00132966 (IPI00373218) | 21.4 | 3 | Similar to peptidylprolyl isomerase D (cyclophilin D) 40 kda peptidyl-prolyl cis-trans isomerase | Peroxisome |
| 1 | IPI00128976 | 30.7 | 6 | Similar to peroxisomal biogenesis factor 16 | unknown |
| 1 | IPI00131342 | 8.1 | 2 | Similar to Peroxisome assembly protein 10 (Peroxin-10) | Peroxisome |
| 0.69 | IPI00365997 | 8.3 | 3 | Similar to PHD finger protein 2 | unknown |
| 0.77 | IPI00361551 | 11.5 | 2 | Similar to phosphatidylglycerophosphate synthase | unknown |
| 1 | IPI00358757 IPI00133034 | 21.5 | 5 | Similar to PKCI-1-related HIT protein 1190005L05Rik protein | unknown |
| 0.99 | IPI00358940 | 7.4 | 3 | Similar to placenta-specific 3 isoform 1 | unknown |
| 1 | IPI00364151 | 28.6 | 23 | Similar to polynucleotide phosphorylase-like protein | unknown |
| 0.79 | IPI00118704 IPI00207671 | 5.8 | 2 | Similar to potassium voltage-gated channel subfamily H member 4 Potassium voltage-gated channel subfamily H member 4 | unknown |
| 1 | IPI00367287 | 11 | 2 | Similar to PR-domain zinc finger protein 6 | mt |
| 1 | IPI00370490 IPI00392898 | 19.7 | 3 | Similar to presenilins associated rhomboid-like protein Ensembl_locations(Chr-bp):11-82894297 | unknown |
| 1 | IPI00359246 | 25.5 | 4 | Similar to probable transmembrane protein FT27 - mouse | unknown |
| 1 | IPI00372370 IPI00227966 (IPI00120100) | 13.3 | 7 | Similar to Prolyl 4-hydroxylase alpha iia subunit Splice isoform iia of Q60716 Prolyl 4-hydroxylase alpha-2 subunit precursor | ER |
| 1 | IPI00308959 | 15.1 | 2 | Similar to proteasome (Prosome macropain) 26S subunit non-atpase 5 | Golgi |
| 1 | IPI00362105 IPI00125971 | 13.4 | 4 | Similar to proteasome 26S atpase subunit 6 26S protease regulatory subunit S10B | unknown |
| 1 | IPI00360232 IPI00109122 IPI00131406 IPI00215243 | 13.7 | 2 | Similar to Proteasome subunit alpha type 7-like Proteasome subunit alpha type 7-like Proteasome subunit alpha type 7 Splice isoform RC6-IL of P48004 | mt |
| 0.99 | IPI00367633 | 16.4 | 2 | Similar to proteasome subunit iota | unknown |
| 1 | IPI00111272  IPI00126010  IPI00215107 | 25.8 | 6 | Similar to protein 40kd 13 days embryo liver cDNA RIKEN full-length enriched library clone:2510038E09 full insert sequence 40S ribosomal protein SA | mt |
| 1 | IPI00372941 | 37.7 | 23 | Similar to Protein CGI-51 | unknown |
| 1 | IPI00365929 | 35.3 | 16 | Similar to Protein disulfide isomerase A6 precursor (Protein disulfide isomerase P5) (Calcium-binding protein 1) (cabp1) | unknown |
| 0.89 | IPI00201274 | 5.4 | 2 | Similar to protein kinase A binding protein AKAP110 | unknown |
| 1 | IPI00343799 IPI00133490 (IPI00394038) | 13.9 | 2 | Similar to protein tyrosine phosphatase 4a1 Protein tyrosine phosphatase 4a1 | mt |
| 0.96 | IPI00369190 | 4.2 | 3 | Similar to PTPL1-associated rhogap 1 | mt |
| 1 | IPI00361164 | 4.1 | 5 | Similar to putative homeobox protein | unknown |
| 1 | IPI00360954 | 36.2 | 3 | Similar to putative NAD(P)H steroid dehydrogenase | unknown |
| 0.61 | IPI00358974 | 11.4 | 2 | Similar to putative pheromone receptor | unknown |
| 1 | IPI00127050 | 35.5 | 13 | Similar to pyrophosphatase | unknown |
| 1 | IPI00123278 | 50.3 | 15 | Similar to pyrroline 5-carboxylate reductase isoform | unknown |
| 1 | IPI00123510 | 49.5 | 17 | Similar to pyrroline-5-carboxylate reductase 1 | unknown |
| 1 | IPI00123004 (IPI00192133) | 12.8 | 5 | Similar to pyruvate dehydrogenase kinase isoenzyme 3 | mt |
| 1 | IPI00169589 | 8.3 | 2 | Similar to quiescin | mt |
| 1 | IPI00362953 | 47.3 | 9 | Similar to Rab18 | mt |
| 1 | IPI00198316 IPI00116770 | 42.7 | 8 | Similar to Rab18 Ras-related protein Rab-18 | mt |
| 1 | IPI00364708 IPI00116760 | 15.8 | 4 | Similar to Rab24 protein Ras-related protein Rab-24 | mt |
| 1 | IPI00371269 IPI00116563 | 27.9 | 3 | Similar to RAB5B member RAS oncogene family Ras-related protein Rab-5B | mt |
| 1 | IPI00357999 IPI00225719 | 7.6 | 3 | Similar to RAE1 RNA export 1 homolog RAE1 RNA export 1 homolog | unknown |
| 0.85 | IPI00368816 | 8.8 | 2 | Similar to Ras-like GTP-binding protein Rem | unknown |
| 1 | IPI00366221 | 49.3 | 13 | Similar to Ras-related protein Rab-21 | unknown |
| 0.72 | IPI00356758 | 7.4 | 2 | Similar to Rbm6 protein | unknown |
| 0.99 | IPI00369409 IPI00311827 IPI00109932 | 7.5 | 2 | Similar to RCK DEAD Probable ATP-dependent RNA helicase p54 | mt |
| 1 | IPI00202231 IPI00133119 | 9.2 | 2 | Similar to RER1 homolog 1110060F11Rik protein | mt |
| 1 | IPI00378680 | 9.9 | 2 | Similar to retinoblastoma-associated factor 600 | mt |
| 0.83 | IPI00359416 | 3.5 | 2 | Similar to retinoblastoma-associated factor 600 | unknown |
| 1 | IPI00379739 | 25.3 | 16 | Similar to Retrovirus-related POL polyprotein | mt |
| 0.61 | IPI00366572 | 5.1 | 2 | Similar to Rho gtpase activating protein 12 | unknown |
| 1 | IPI00347262 IPI00123007 (IPI00370569) (IPI00213988) (IPI00361677) (IPI00231042) (IPI00378679) IPI00392363 (IPI00361934) IPI00192995 (IPI00392307) (IPI00362828) IPI00207486 (IPI00361628) IPI00390235 IPI00388786 (IPI00348567) IPI00393887 (IPI00390860) | 13.5 | 2 | Similar to ribosomal protein L31 60S ribosomal protein L31  Ensembl_locations(Chr-bp):4-118853380  Ensembl_locations(Chr-bp):12-17443213  Ensembl_locations(Chr-bp):5-79535139  Ensembl_locations(Chr-bp):4-31024963 Ensembl_locations(Chr-bp):18-40371973  Ensembl_locations(Chr-bp):4-80667909 | mt |
| 0.99 | IPI00373522 | 31.5 | 2 | Similar to ribosomal protein L7 cytosolic | cytoplasm |
| 1 | IPI00369061 | 46.4 | 4 | Similar to ribosomal protein S12 | mt |
| 1 | IPI00210964 | 28 | 5 | Similar to ribosomal protein S5 | unknown |
| 1 | IPI00368950 | 18.6 | 7 | Similar to ribosomal protein S9 | mt |
| 1 | IPI00195374 IPI00133211 (IPI00315230) | 11.8 | 2 | Similar to RIKEN cDNA 0610009E20 0610009E20Rik protein | mt |
| 1 | IPI00121440 | 63.9 | 29 | Similar to RIKEN cDNA 0610009I16 gene | mt |
| 1 | IPI00366610 | 32.9 | 13 | Similar to RIKEN cDNA 0610027A18 | mt |
| 1 | IPI00206743 IPI00116077 | 34.5 | 9 | Similar to RIKEN cDNA 0610039A15 Hypothetical protein | unknown |
| 1 | IPI00365087 IPI00133110 | 17.6 | 2 | Similar to RIKEN cDNA 0610040B21 Thioredoxin-like protein p19 precursor | mt |
| 1 | IPI00372027 | 20 | 15 | Similar to RIKEN cDNA 0610041L09 | unknown |
| 1 | IPI00364520 | 13.7 | 4 | Similar to RIKEN cDNA 0710001P09 | mt |
| 1 | IPI00373757 IPI00133778 | 29.7 | 7 | Similar to RIKEN cDNA 1110006I11 1110006I11Rik protein | unknown |
| 0.87 | IPI00364878 IPI00315974 | 9.8 | 2 | Similar to RIKEN cDNA 1110007C09 RIKEN cDNA 1110007C09 | mt |
| 1 | IPI00212516 IPI00153468 | 27.3 | 6 | Similar to RIKEN cDNA 1110014L17 P26 protein | unknown |
| 1 | IPI00372007 | 45.6 | 2 | Similar to RIKEN cDNA 1110018B13 | mt |
| 0.72 | IPI00361787 | 14.1 | 3 | Similar to RIKEN cDNA 1110031I02 | unknown |
| 0.97 | IPI00360295 | 8.9 | 2 | Similar to RIKEN cDNA 1190005P08 gene | unknown |
| 1 | IPI00369308 IPI00321744 | 10.2 | 3 | Similar to RIKEN cDNA 1200007D18 RIKEN cDNA 1200007D18 | mt |
| 1 | IPI00362579 IPI00310692 IPI00119695 IPI00390681 | 13 | 6 | Similar to RIKEN cDNA 1300011P19 RIKEN cDNA 1300011P19 gene 1300011P19Rik protein Ensembl_locations(Chr-bp):8-35001063 | mt |
| 1 | IPI00199324 | 27.4 | 5 | Similar to RIKEN cDNA 1500009M05 | unknown |
| 0.9 | IPI00367367 | 8.5 | 3 | Similar to RIKEN cDNA 1700001E04 | unknown |
| 1 | IPI00359018 IPI00322562 (IPI00201500) | 30.5 | 4 | Similar to RIKEN cDNA 1810007P19 40S ribosomal protein S14 | unknown |
| 1 | IPI00369654 IPI00111884 | 14 | 2 | Similar to RIKEN cDNA 1810012H11 1810012H11Rik protein | mt |
| 1 | IPI00189852 (IPI00188291) | 19.8 | 2 | Similar to RIKEN cDNA 2010110M21 | mt |
| 1 | IPI00208184 IPI00133411 | 13 | 2 | Similar to RIKEN cDNA 2010309E21 2010309E21Rik protein | mt |
| 1 | IPI00362609 IPI00132055 | 10.7 | 4 | Similar to RIKEN cDNA 2010315L10 2010315L10Rik protein | unknown |
| 0.86 | IPI00358813 IPI00318505 IPI00119169 | 8.1 | 2 | Similar to RIKEN cDNA 2210021A15 Mediator of RNA polymerase II transcription subunit 8 homolog 2210021A15Rik protein | unknown |
| 1 | IPI00362920 | 27.1 | 14 | Similar to RIKEN cDNA 2210404D11 | unknown |
| 1 | IPI00365572 IPI00394116 IPI00280867 (IPI00230098) | 4.5 | 2 | Similar to RIKEN cDNA 2310034L04 Ensembl_locations(Chr-bp):6-20889353 Hypothetical protein | mt |
| 1 | IPI00213015 IPI00116112 | 11.7 | 3 | Similar to RIKEN cDNA 2310042E05 Dynactin complex 50 kda subunit | mt |
| 1 | IPI00195673 IPI00122928 | 30.6 | 9 | Similar to RIKEN cDNA 2310057H16 Hypothetical protein | unknown |
| 1 | IPI00362229 IPI00153990 | 30.6 | 5 | Similar to RIKEN cDNA 2310066K23 Similar to ARP1 actin-related protein 1 homolog B centractin beta | Nuleus |
| 1 | IPI00188468 IPI00109033 | 18.7 | 8 | Similar to RIKEN cDNA 2410166I05 2410166I05Rik protein | mt |
| 1 | IPI00190482 IPI00113239 | 17.1 | 3 | Similar to RIKEN cDNA 2610319K07 2610319K07Rik protein | unknown |
| 1 | IPI00357893 | 19 | 11 | Similar to RIKEN cDNA 2610509I15 | mt |
| 1 | IPI00116102 IPI00360108 | 9.8 | 2 | Similar to RIKEN cDNA 2610510H03 gene Similar to RIKEN cDNA 2810405J04 | mt |
| 0.99 | IPI00364789 | 3.8 | 2 | Similar to RIKEN cDNA 2810013E07 | mt |
| 1 | IPI00208337 IPI00124389 | 26.6 | 2 | Similar to RIKEN cDNA 2810014D17 Hypothetical protein | unknown |
| 0.7 | IPI00358031 | 8.8 | 2 | Similar to RIKEN cDNA 2810421I24 | unknown |
| 0.74 | IPI00370853 | 6.4 | 2 | Similar to RIKEN cDNA 2810429C13 | unknown |
| 1 | IPI00364713 | 19.3 | 4 | Similar to RIKEN cDNA 2900010M23 | unknown |
| 0.76 | IPI00366031 | 11.7 | 2 | Similar to RIKEN cDNA 3230401I01 | unknown |
| 1 | IPI00362949 | 36.1 | 19 | Similar to RIKEN cDNA 4430402G14 | unknown |
| 1 | IPI00197896 IPI00128201 | 21.3 | 3 | Similar to RIKEN cDNA 4633402N23 gene RIKEN cDNA 4633402N23 gene | mt |
| 1 | IPI00364385 IPI00228236 | 21.2 | 5 | Similar to RIKEN cDNA 5730406I15 Microsomal signal peptidase 25 kda subunit | mt |
| 1 | IPI00361819 | 48 | 3 | Similar to RIKEN cDNA 6030432N09 | mt |
| 1 | IPI00361738 | 50.9 | 12 | Similar to RIKEN cDNA 9430083G14 | mt |
| 1 | IPI00358033 | 33.7 | 22 | Similar to RIKEN cDNA 9930026A05 | unknown |
| 1 | IPI00372804 | 16.1 | 7 | Similar to RIKEN cDNA C330023F11 | unknown |
| 1 | IPI00361193 | 39.5 | 16 | Similar to RIKEN cDNA D330038I09 | unknown |
| 1 | IPI00366249 IPI00339468 | 4.3 | 4 | Similar to RNA helicase A ATP-dependent RNA helicase A | mt |
| 1 | IPI00364603 | 5.8 | 3 | Similar to RNA-binding protein EWS | mt |
| 1 | IPI00209905  IPI00132397 | 14.1 | 2 | Similar to Sar1a protein promoting vesicle budding from the endoplasmic reticulum GTP-binding protein SAR1b | membrane |
| 0.99 | IPI00116752 | 7.1 | 2 | Similar to Sec23-interacting protein p125 | mt |
| 1 | IPI00331016 IPI00372727 | 4.8 | 5 | Similar to SEC24 related gene family member B Similar to Protein transport protein Sec24B (SEC24-related protein B) | unknown |
| 1 | IPI00364926 IPI00227631 | 13.9 | 3 | Similar to Sec61 alpha isoform 2 Sec61 alpha subunit 2 | mt |
| 0.9 | IPI00372530 | 11.4 | 3 | Similar to semaphorin subclass 4 member G | unknown |
| 1 | IPI00224178 IPI00363930 | 17.9 | 3 | Similar to SEPTIN6 type II Similar to hypothetical protein FLJ10849 | mt |
| 1 | IPI00377750 | 19.2 | 5 | Similar to septin-like protein | unknown |
| 1 | IPI00195109 | 65 | 43 | Similar to serine hydroxymethyl transferase 2 (mitochondrial) | mt |
| 1 | IPI00408085 | 4.8 | 5 | Similar to SI:zc220f6.1 (novel protein Similar to human dynein heavy chain (DHC)) | mt |
| 0.61 | IPI00391713 | 25.9 | 3 | Similar to sialic acid binding Ig-like lectin 5 | unknown |
| 1 | IPI00408243 | 48 | 5 | Similar to single-stranded DNA binding protein | mt |
| 0.8 | IPI00381529 | 10.4 | 2 | Similar to Small nuclear ribonucleoprotein associated protein N (snrnp-N) (Sm protein N) (Sm-N) (smn) (Sm-D) (Tissue-specific splicing protein) | unknown |
| 0.72 | IPI00379321 | 6.8 | 2 | Similar to small-conductance calcium-activated potassium channel | unknown |
| 0.99 | IPI00358733 | 5 | 3 | Similar to SMC2 protein | unknown |
| 1 | IPI00345966 | 7.7 | 3 | Similar to Smith-Magenis syndrome chromosome region, candidate 7 | mt |
| 0.93 | IPI00365570 | 8.3 | 3 | Similar to smooth muscle myosin heavy chain 11 isoform SM1-like | unknown |
| 1 | IPI00379181 | 8.6 | 2 | Similar to solute carrier family 25 member 5 | mt |
| 0.78 | IPI00363032 (IPI00393035) | 5.8 | 2 | Similar to Sorbitol dehydrogenase (L-iditol 2-dehydrogenase) | unknown |
| 1 | IPI00373419 | 18.5 | 6 | Similar to Spectrin beta chain brain 1 (Spectrin non-erythroid beta chain 1) (Beta-II spectrin) (Fodrin beta chain) | unknown |
| 0.77 | IPI00361140 | 6.8 | 2 | Similar to sphingomyelin phosphodiesterase 3 neutral | Golgi |
| 0.64 | IPI00368841 | 9.5 | 2 | Similar to Src-related intestinal kinase | unknown |
| 1 | IPI00203528 | 55.8 | 7 | Similar to stomatin-like protein 2 | mt |
| 0.88 | IPI00407571 IPI00126902 (IPI00231263) | 6.8 | 2 | Similar to structure specific recognition protein 1 Structure-specific recognition protein 1 | unknown |
| 1 | IPI00358377 | 53.7 | 4 | Similar to Succinyl-coa ligase | mt |
| 1 | IPI00314919 | 12.1 | 7 | Similar to suppressor of var1 3-like 1 | unknown |
| 1 | IPI00213458 IPI00133249 | 8.6 | 3 | Similar to Surf-4 protein - mouse Surfeit locus protein 4 | membrane |
| 0.75 | IPI00352983 | 6.2 | 2 | Similar to SWI\SNF related matrix associated actin dependent regulator of chromatin subfamily a member 3 | unknown |
| 1 | IPI00360156 | 19 | 7 | Similar to synaptic glycoprotein SC2 | mt |
| 0.96 | IPI00360322 | 3.3 | 2 | Similar to tangerin | unknown |
| 0.63 | IPI00188528 | 17.5 | 2 | Similar to testis and spermatogenesis cell apoptosis related protein 1 | unknown |
| 1 | IPI00371036 IPI00132217 | 15.8 | 2 | Similar to tetratricopeptide repeat domain 11 Tetratricopeptide repeat protein 11 | mt |
| 1 | IPI00194851 | 30.7 | 4 | Similar to thioesterase superfamily member 2 | mt |
| 1 | IPI00364866 IPI00134058 | 16.5 | 6 | Similar to thioredoxin domain containing 4 Thioredoxin domain containing protein 4 precursor | unknown |
| 1 | IPI00365481 | 41.2 | 33 | Similar to TOB3 | unknown |
| 1 | IPI00197411  IPI00118447 | 8.1 | 2 | Similar to transcriptional activator protein pure-alpha (purine-rich single-stranded DNA-binding protein alpha)  Transcriptional activator protein PUR-alpha | mt |
| 0.99 | IPI00128071 | 5 | 2 | Similar to transcytosis associated protein | nuleus |
| 1 | IPI00319369 | 41.6 | 16 | Similar to transducin (Beta)-like 2 | unknown |
| 1 | IPI00364138 | 17.5 | 13 | Similar to transforming growth factor beta regulated gene 4 | mt |
| 1 | IPI00358634 | 14.5 | 3 | Similar to Translationally controlled tumor protein (TCTP) (p23) (21 kda polypeptide) (p21) (Lens epithelial protein) | mt |
| 1 | IPI00387579 | 51.7 | 3 | Similar to translocase of inner mitochondrial membrane 23 homolog | mt |
| 1 | IPI00365982 | 44.2 | 41 | Similar to transmembrane protein (63kd) endoplasmic reticulum\Golgi interm | ER |
| 1 | IPI00366450 | 7.3 | 2 | Similar to tRNA (5-methylaminomethyl-2-thiouridylate)-methyltransferase | mt |
| 1 | IPI00330464 | 10 | 3 | Similar to tudor and KH domain containing protein | unknown |
| 1 | IPI00153381 | 26.6 | 2 | Similar to ubiquinol-cytochrome c reductase complex | mt |
| 1 | IPI00369093  IPI00129516 | 47.2 | 10 | Similar to Ubiquinol-cytochrome C reductase complex 11 kda protein itochondrial precursor (Mitochondrial hinge protein) (Cytochrome C1 nonheme 11 kda protein) (Complex III subunit VIII) Ubiquinol-cytochrome C reductase complex 11 kda protein mitochondrial precursor | unknown |
| 1 | IPI00368347 IPI00123313 | 10.5 | 5 | Similar to ubiquitin-protein ligase (EC 6.3.2.19) E1 - mouse Ubiquitin-activating enzyme E1 1 | mt |
| 0.99 | IPI00381159 (IPI00365637) IPI00310557 | 5.6 | 2 | Similar to UDP-galnac:polypeptide N-acetylgalactosaminyltransferase T10  Similar to human KIAA1130 protein | mt |
| 1 | IPI00126072 | 28.3 | 10 | Similar to vesicle amine transport protein 1 | unknown |
| 1 | IPI00193522 IPI00114368 | 39.5 | 12 | Similar to vesicle trafficking protein sec22b Vesicle trafficking protein SEC22b | mt |
| 1 | IPI00230453 IPI00357887 | 21.8 | 5 | Similar to VIP36-like protein precursor (Lectinmannose-binding 2-like) Similar to lectin mannose-binding 2-like | mt |
| 0.73 | IPI00372689 | 5.7 | 3 | Similar to VLA-3 alpha subunit | unknown |
| 1 | IPI00370315 IPI00222560 IPI00124742 | 12.7 | 2 | Similar to Wbscr1 Splice isoform Short of Q9WUK2 Eukaryotic translation initiation factor 4H Splice isoform Long of Q9WUK2 Eukaryotic translation initiation factor 4H | mt |
| 0.74 | IPI00362896 | 6.2 | 3 | Similar to WDR9 protein form A | unknown |
| 0.84 | IPI00370343 | 8.1 | 2 | Similar to WSB-1 | unknown |
| 0.66 | IPI00349122 | 4.2 | 2 | Similar to zinc finger 5 protein | unknown |
| 0.6 | IPI00191045 | 16.4 | 2 | Similar to zinc finger protein 296 | unknown |
| 0.97 | IPI00360294 (IPI00342123) | 5.1 | 2 | Similar to zinc finger protein 366 | unknown |
| 0.64 | IPI00343936 | 1.7 | 2 | Similar to zinc finger protein 407 | unknown |
| 0.87 | IPI00371741 | 9.4 | 2 | Similar to zinc finger protein TZF-L | unknown |
| 0.61 | IPI00213355 | 6.7 | 2 | Similar to zinedin | unknown |
| 1 | IPI00196750 | 47.7 | 2 | Single-stranded DNA-binding protein mitochondrial precursor | mt |
| 1 | IPI00210733 IPI00132410 | 33.5 | 4 | Small GTP-binding protein rab5 Ras-related protein Rab-5A | membrane |
| 0.96 | IPI00309724 | 5 | 2 | Smap-1b | unknown |
| 1 | IPI00200392 IPI00177183 | 8.1 | 2 | SMHS2 RIKEN cDNA 4833427E09 gene | mt |
| 0.93 | IPI00201085 | 13.8 | 2 | Socius | unknown |
| 1 | IPI00308162 | 49.3 | 30 | Solute carrier family 25 | mt |
| 1 | IPI00230754 | 51 | 32 | Solute carrier family 25 (mitochondrial carrier | mt |
| 0.72 | IPI00208799 | 10 | 2 | Solute carrier organic anion transporter family member 1A5 | unknown |
| 0.79 | IPI00123314 | 9.4 | 2 | Sonic hedgehog protein precursor | mt |
| 0.99 | IPI00198369 (IPI00125441) | 7.7 | 2 | Sorting nexin 1 | mt |
| 1 | IPI00210090 IPI00177229 | 19.7 | 13 | SP120 Similar to transporter protein system N1 Na+ and H+-coupled glutamine transporter | mt |
| 1 | IPI00319830 | 18.1 | 5 | Spectrin beta chainbrain 1 | mt |
| 1 | IPI00320480 | 21.1 | 8 | Sphingosine-1-phosphate lyase 1 | unknown |
| 1 | IPI00116697 IPI00361149 | 42.3 | 6 | Splice isoform 1 of P35279 Ras-related protein Rab-6A Similar to RAB6 member RAS oncogene family | mt |
| 1 | IPI00118875 | 26.7 | 7 | Splice isoform 1 of P57776 Elongation factor 1-delta | unknown |
| 1 | IPI00400401 | 5.7 | 4 | Splice isoform 1 of Q80Y81 Zinc phosphodiesterase ELAC protein 2 | unknown |
| 1 | IPI00131138 | 24.3 | 23 | Splice isoform 1 of Q8BTM8 Filamin A | mt |
| 1 | IPI00228150 | 54 | 6 | Splice isoform 1 of Q8CAQ8 Mitochondrial inner membrane protein | mt |
| 1 | IPI00126115 IPI00200069 | 24.3 | 2 | Splice isoform 1 of Q91V61 Sideroflexin 3 Sideroflexin 3 | mt |
| 1 | IPI00118193 | 33.9 | 5 | Splice isoform 1 of Q99N84 28S ribosomal protein s18b mitochondrial precursor | mt |
| 1 | IPI00109603 | 21.7 | 7 | Splice isoform 1 of Q9CWU6 Basic FGF-repressed Zic binding protein | mt |
| 1 | IPI00275050 | 26.1 | 3 | Splice isoform 1 of Q9ER88 Mitochondrial 28S ribosomal protein S29 | unknown |
| 1 | IPI00119846 | 22.2 | 15 | Splice isoform 1 of Q9JI39 ATP-binding cassette sub-family B member 10 mitochondrial precursor | mt |
| 0.76 | IPI00200928 IPI00230819 | 9.2 | 2 | Splice isoform 1 of Q9JJ22 Splice isoform 2 of Q9JJ22 | unknown |
| 1 | IPI00400079 IPI00124699 IPI00403407  IPI00350590 | 20.1 | 3 | Splice isoform 1 of Q9JLT4 Thioredoxin reductase 2 mitochondrial precursor Thioredoxin reductase 2 Adult male stomach cDNA RIKEN full-length enriched library clone:2210009O12 product:thioredoxin reductase 2 full insert sequence Splice isoform 4 of Q9JLT4 Thioredoxin reductase 2 mitochondrial precursor | unknown |
| 1 | IPI00124900  IPI00364715 | 15 | 4 | Splice isoform 1 of Q9JLZ3 Methylglutaconyl-coa hydratase mitochondrial precursor Similar to Auh protein | mt |
| 0.72 | IPI00214174 | 4.5 | 2 | Splice isoform 1 of Q9QYJ4 | unknown |
| 0.99 | IPI00323481 IPI00323483 | 4 | 2 | Splice isoform 1 of Q9WU78 Programmed cell death 6 interacting protein Splice isoform 3 of Q9WU78 Programmed cell death 6 interacting protein | mt |
| 1 | IPI00230590 IPI00230589 IPI00230593 IPI00115506 IPI00230592 IPI00230591 | 21 | 4 | Splice isoform 2 of O08715 A kinase anchor protein 1 mitochondrial precursor Splice isoform 1 of O08715 A kinase anchor protein 1 mitochondrial precursor Splice isoform 6 of O08715 A kinase anchor protein 1 mitochondrial precursor Splice isoform 3 of O08715 A kinase anchor protein 1 mitochondrial precursor Splice isoform 5 of O08715 A kinase anchor protein 1 mitochondrial precursor Splice isoform 4 of O08715 A kinase anchor protein 1 mitochondrial precursor | Nuleus |
| 1 | IPI00194958 IPI00404438  IPI00404434  IPI00194959  IPI00404436  IPI00264501  IPI00321378  IPI00193580  IPI00404435 | 8.7 | 2 | Splice isoform 2 of O55012 Splice isoform 6 of Q7M6Y3 Phosphatidylinositol-binding clathrin assembly protein Splice isoform 2 of Q7M6Y3 Phosphatidylinositol-binding clathrin assembly protein Splice isoform 1 of O55012 Splice isoform 4 of Q7M6Y3 Phosphatidylinositol-binding clathrin assembly protein Splice isoform 1 of Q7M6Y3 Phosphatidylinositol-binding clathrin assembly protein Splice isoform 5 of Q7M6Y3 Phosphatidylinositol-binding clathrin assembly protein Ensembl_locations(Chr-bp):1-146832515 Splice isoform 3 of Q7M6Y3 Phosphatidylinositol-binding clathrin assembly protein | membrane |
| 1 | IPI00402981 IPI00214654 IPI00378082 IPI00408720 (IPI00360855) IPI00318368 (IPI00231024) | 20.3 | 4 | Splice isoform 2 of P16632 40S ribosomal protein S24 Splice isoform 1 of P16632 Similar to ribosomal protein S24 Ensembl_locations(Chr-bp):X-69182520  Ribosomal protein S24 | Mt |
| 1 | IPI00230044 IPI00372259 | 20.6 | 5 | Splice isoform 2 of P21107 Tropomyosin alpha 3 chain Tropomyosin isoform 6 | mt |
| 1 | IPI00230640  IPI00329913 | 31.3 | 6 | Splice isoform 2 of P23506 Protein-L-isoaspartate(D-aspartate) O-methyltransferase Protein-L-isoaspartate | mt |
| 0.99 | IPI00230492 IPI00388257 IPI00132067 | 6.5 | 3 | Splice isoform 2 of P37889 Fibulin-2 precursor Fibulin-2 Splice isoform 1 of P37889 Fibulin-2 precursor | mt |
| 1 | IPI00336282 IPI00336281 | 8 | 6 | Splice isoform 2 of P55937 Golgi autoantigen golgin subfamily A member 3 Splice isoform 1 of P55937 Golgi autoantigen golgin subfamily A member 3 | mt |
| 1 | IPI00229669 IPI00370028 IPI00113660 | 16.1 | 3 | Splice isoform 2 of Q12920 Proteasome activator complex subunit 3 Similar to Ki antigen Splice isoform 1 of Q12920 Proteasome activator complex subunit 3 | unknown |
| 1 | IPI00409936 IPI00308709 | 16.2 | 11 | Splice isoform 2 of Q8BKC5 Importin beta-3 Splice isoform 1 of Q8BKC5 Importin beta-3 | mt |
| 1 | IPI00381412 | 53.4 | 5 | Splice isoform 2 of Q8CAQ8 Mitochondrial inner membrane protein | unknown |
| 1 | IPI00279858 | 45 | 17 | Splice isoform 2 of Q99M87 DNAj homolog subfamily A member 3 mitochondrial precursor | mt |
| 0.68 | IPI00407425 IPI00338519 | 3.2 | 3 | Splice isoform 2 of Q9JMH9 Myosin XVIIIA Splice isoform 1 of Q9JMH9 Myosin XVIIIA | unknown |
| 0.77 | IPI00225140 IPI00400052 IPI00136902 (IPI00408623) | 1.6 | 2 | Splice isoform 2 of Q9QYX7 Piccolo protein Ensembl_locations(Chr-bp):5-13231557 Splice isoform 1 of Q9QYX7 Piccolo protein | unknown |
| 1 | IPI00351315 | 6.1 | 2 | Splice isoform 2 of Q9R049 Autocrine motility factor receptor | mt |
| 1 | IPI00231067 | 56 | 14 | Splice isoform 2 of Q9R1Z0 Voltage-dependent anion-selective channel protein 3 | mt |
| 1 | IPI00284272 IPI00230690 IPI00230692 IPI00113690 | 3.5 | 3 | Splice isoform 3 of Q91ZU6 Bullous pemphigoid antigen 1 isoforms 1\2\3\4 Splice isoform 2 of Q91ZU6 Bullous pemphigoid antigen 1 isoforms 1\2\3\4 Splice isoform 4 of Q91ZU6 Bullous pemphigoid antigen 1 isoforms 1\2\3\4 Ensembl_locations(Chr-bp):1-34597334 | unknown |
| 1 | IPI00230444 IPI00129527 IPI00230445 | 22.6 | 9 | Splice isoform 3u of P15331 Peripherin Splice isoform 5g of P15331 Peripherin Splice isoform 5b of P15331 Peripherin | mt |
| 0.64 | IPI00407014 IPI00407013 IPI00407012 IPI00121608 | 6.2 | 2 | Splice isoform 4 of Q99PV8 B-cell lymphoma\leukemia 11B Splice isoform 3 of Q99PV8 B-cell lymphoma\leukemia 11B Splice isoform 2 of Q99PV8 B-cell lymphoma\leukemia 11B Splice isoform 1 of Q99PV8 B-cell lymphoma\leukemia 11B | unknown |
| 1 | IPI00229538 IPI00229535 IPI00229536 IPI00113686 IPI00395163 IPI00229537 | 16.9 | 10 | Splice isoform 5 of Q9ESZ8 General transcription factor II-I Splice isoform 2 of Q9ESZ8 General transcription factor II-I Splice isoform 3 of Q9ESZ8 General transcription factor II-I Splice isoform 1 of Q9ESZ8 General transcription factor II-I Splice isoform 6 of Q9ESZ8 General transcription factor II-I Splice isoform 4 of Q9ESZ8 General transcription factor II-I | unknown |
| 1 | IPI00314806 IPI00338150 | 7.9 | 4 | Splice isoform A of Q9WTI7 Myosin Ic Splice isoform B of Q9WTI7 Myosin Ic | mt |
| 0.98 | IPI00231480 IPI00113866 IPI00191787 IPI00191753 IPI00191760 IPI00325778 IPI00264215 IPI00231479 (IPI00131593) IPI00231481 IPI00113865 IPI00199399 IPI00231482 IPI00231483 | 8.4 | 2 | Splice isoform Alpha2B of P43322 Neuregulin 1 SMDF neuregulin beta 1a Glial growth factor beta 1a SMDF neuregulin alpha 2b Splice isoform beta2a of P43322 Ensembl_locations(Chr-bp):8-30806215 Splice isoform Alpha2A of P43322  Splice isoform Alpha2C of P43322 Neuregulin 1-beta 1 Splice isoform Beta4 of P43322 Splice isoform Beta1 of P43322 Splice isoform Beta2 of P43322 | unknown |
| 1 | IPI00231268 IPI00194873 IPI00231271 IPI00231269 | 14.3 | 10 | Splice isoform B of P11505 Splice isoform D of P11505 Splice isoform K of P11505 Splice isoform C of P11505 | membrane |
| 1 | IPI00230037 IPI00127408 IPI00358641 | 19.3 | 6 | Splice isoform B of P15154 Ras-related C3 botulinum toxin substrate 1 Splice isoform A of P15154 Ras-related C3 botulinum toxin substrate 1 Similar to ras-related C3 botulinum toxin substrate 1 isoform Rac1b | unknown |
| 0.99 | IPI00125058 | 5.6 | 4 | Splice isoform B of Q61789 Laminin alpha-3 chain precursor | mt |
| 1 | IPI00197553 IPI00127415 | 21.6 | 12 | Splice isoform B23.1 of P13084 Nucleophosmin | mt |
| 1 | IPI00406106 | 21.4 | 17 | Splice isoform HK1 of P17710 Hexokinase type I | mt |
| 1 | IPI00212980 IPI00131896 IPI00232012 | 25 | 7 | Splice isoform Long of P38718 Brain protein 44 Splice isoform Short of P38718 | mt |
| 1 | IPI00352793 IPI00129808 IPI00224251  IPI00231751 | 18.4 | 5 | Splice isoform Long of P49312 Heterogeneous nuclear ribonucleoprotein A1 Ensembl_locations(Chr-bp):9-126073320 Similar to heterogeneous nuclear ribonucleoprotein A1 (Helix-destabilizing protein) (Single-strand binding protein) (hnrnp core protein A1) (HDP-1) (Topoisomerase-inhibitor suppressed) Heterogeneous nuclear ribonucleoprotein A1 | mt |
| 1 | IPI00129350 | 44.8 | 51 | Splice isoform Long of Q9Z110 Delta 1-pyrroline-5-carboxylate synthetase | mt |
| 1 | IPI00112822  IPI00230512 | 21.4 | 6 | Splice isoform Mitochondrial of P40630 Transcription factor A mitochondrial precursor Splice isoform Nuclear of P40630 Transcription factor A mitochondrial precursor | mt |
| 1 | IPI00230540 (PI00122549) | 79.9 | 14 | Splice isoform Mt-VDAC1 of Q60932 Voltage-dependent anion-selective channel protein 1 | mt |
| 1 | IPI00227866  IPI00231369 (IPI00325441) | 31.6 | 22 | Splice isoform SERCA2A of O55143 Sarcoplasmic\endoplasmic reticulum calcium atpase 2 Splice isoform SERCA2A of P11507 | unknown |
| 1 | IPI00338964 | 31.5 | 23 | Splice isoform SERCA2B of O55143 Sarcoplasmic\endoplasmic reticulum calcium atpase 2 | ER |
| 0.92 | IPI00388681 IPI00114869 IPI00194555 IPI00119714 IPI00231187 IPI00223407 | 4.5 | 2 | Splice isoform UBF2 of P25977 Splice isoform UBF1 of P25976 Nucleolar transcription factor 1 Splice isoform UBF1 of P25977 Transcription factor UBF Ensembl_locations(Chr-bp):10-91400388 Splice isoform UBF2 of P25976 Nucleolar transcription factor 1 | unknown |
| 1 | IPI00330207 IPI00366952 | 6.5 | 4 | Splicing factor 3B subunit 1 Splicing factor 3B subunit 1 155kd | mt |
| 1 | IPI00122217 | 12.9 | 5 | Squalene monooxygenase | mt |
| 1 | IPI00123709 | 44.4 | 47 | Ssecks | mt |
| 1 | IPI00153959 | 6.3 | 6 | Stabilin-1 | membrane |
| 1 | IPI00225301 | 37.9 | 7 | Steroid dehydrogenase-like protein homolog | mt |
| 1 | IPI00133903 | 51.1 | 80 | Stress-70 protein mitochondrial precursor | mt |
| 1 | IPI00121514 IPI00213013 | 28.7 | 10 | Stress-induced phosphoprotein 1 P60 protein | mt |
| 1 | IPI00227657 | 14.7 | 2 | Stromal cell-derived factor 2-like protein 1 precursor | ER |
| 1 | IPI00201289 (IPI00132122) | 3.1 | 2 | Structural maintenance of chromosome 3 | mt |
| 1 | IPI00319111 | 31.4 | 8 | Succinate dehydrogenase cytochrome b560 subunit mitochondrial precursor | mt |
| 1 | IPI00357872 | 45.3 | 4 | Succinate-Coenzyme A ligase GDP-forming beta subunit | mt |
| 1 | IPI00131183 IPI00261627 | 45.8 | 17 | Succinyl-coa ligase [ADP-forming] beta-chain mitochondrial precursor Succinate-coenzyme A ligase | mt |
| 1 | IPI00406442 IPI00124821 | 26.1 | 12 | Succinyl-coa ligase [GDP-forming] alpha-chain mitochondrial precursor Succinate-coa ligase GDP-forming alpha subunit | mt |
| 1 | IPI00197555 | 32.7 | 10 | Succinyl-coa ligase [GDP-forming] alpha-chain mitochondrial precursor | unknown |
| 1 | IPI00313998 | 14.7 | 3 | Sulfide:quinone oxidoreductase mitochondrial precursor | mt |
| 1 | IPI00109109 | 57.2 | 10 | Superoxide dismutase [Mn] mitochondrial precursor | mt |
| 1 | IPI00231643 IPI00130589 | 15.6 | 2 | Superoxide dismutase 1 Superoxide dismutase 1 soluble | unknown |
| 1 | IPI00119097 | 23.2 | 13 | Suppressor of potassium transport defect 3 | unknown |
| 1 | IPI00319135 | 18.9 | 4 | Surfeit gene 1 | mt |
| 1 | IPI00205132 (IPI00109206) IPI00170212 | 15.2 | 2 | Synaptojanin 2 binding protein  Activin receptor interacting protein 2 | mt |
| 0.99 | IPI00123505 (IPI00211360) | 7.5 | 2 | Synaptophysin | nuleus |
| 1 | IPI00111416 | 11.3 | 2 | Syntaxin 12 | mt |
| 0.99 | IPI00117333 | 9.7 | 2 | Taipoxin-associated calcium binding protein 49 | mt |
| 1 | IPI00110560 IPI00362014 | 5.7 | 9 | Talin 1 Similar to talin | mt |
| 1 | IPI00118677 | 48.7 | 19 | T-complex protein 1 alpha subunit B | mt |
| 1 | IPI00320217 | 60.4 | 37 | T-complex protein 1 beta subunit | mt |
| 1 | IPI00116279 | 30.9 | 13 | T-complex protein 1 epsilon subunit | mt |
| 1 | IPI00331174 IPI00364286 | 28.9 | 15 | T-complex protein 1 eta subunit Similar to ccteta eta subunit of the chaperonin containing TCP-1 (CCT) | mt |
| 1 | IPI00331185 | 26.8 | 16 | T-complex protein 1 theta subunit | mt |
| 1 | IPI00116281 IPI00188111 | 32.4 | 15 | T-complex protein 1 zeta subunit Similar to CCT (chaperonin containing TCP-1) zeta subunit | membrane |
| 1 | IPI00116277 | 59.2 | 33 | T-complex protein 1 delta subunit | mt |
| 0.71 | IPI00405386 | 7 | 2 | T-complex protein 10a | mt |
| 1 | IPI00127989 IPI00379563 IPI00365935 | 18.8 | 2 | Telomerase-binding protein p23 Similar to Sid3177p Similar to Sid3177p | mt |
| 0.93 | IPI00404334 IPI00130794 | 2.4 | 3 | Tenascin-x Tenascin x | unknown |
| 1 | IPI00123783 | 4.5 | 4 | Ten-m3 | unknown |
| 0.87 | IPI00125705 | 7.6 | 2 | Tes101rp | unknown |
| 1 | IPI00311576 | 7.3 | 2 | Testis expressed gene 10 | mt |
| 1 | IPI00132958 | 30.7 | 5 | Thioesterase superfamily member 2 | mt |
| 1 | IPI00206399 (IPI00125652) | 36.7 | 5 | Thioredoxin mitochondrial precursor | mt |
| 1 | IPI00116192 | 36.6 | 15 | Thioredoxin-dependent peroxide reductase mitochondrial precursor | mt |
| 1 | IPI00366293 | 27.3 | 7 | Thiosulfate sulfurtransferase | mt |
| 1 | IPI00223216 | 21.2 | 5 | Thiosulfate sulfurtransferase mitochondrial | mt |
| 0.89 | IPI00118413 | 10.7 | 3 | Thrombospondin 1 precursor | unknown |
| 1 | IPI00205466 IPI00137811 | 9.9 | 11 | TIP120 TBP-interacting protein | nucleus |
| 0.73 | IPI00310128 | 22.7 | 2 | Tissue inhibitor of metalloproteinase 2 | mt |
| 0.92 | IPI00373635 | 2 | 4 | Titin | unknown |
| 0.79 | IPI00408182 | 2.2 | 2 | Tmc3 protein | unknown |
| 1 | IPI00126913 | 44.2 | 34 | Tob3 | mt |
| 1 | IPI00207732 | 28.7 | 3 | Topoisomerase (DNA) 2 alpha | nucleus |
| 1 | IPI00125907 | 7 | 2 | Tram1 | mt |
| 1 | IPI00323130 IPI00389386 IPI00389443 IPI00393533 (IPI00206015) IPI00188572 | 18 | 2 | Transcription elongation factor B polypeptide 1 Ensembl_locations(Chr-bp):12-17543109 Ensembl_locations(Chr-bp):16-21611476 Ensembl_locations(Chr-bp):16-20785713  Ensembl_locations(Chr-bp):16-257901 | mt |
| 0.9 | IPI00133262 | 3.6 | 2 | Transcription factor 8 | unknown |
| 0.88 | IPI00312128 | 5.4 | 2 | Transcription intermediary factor 1-beta | unknown |
| 1 | IPI00344360 | 8.4 | 2 | Transcription termination factor mitochondrial | mt |
| 1 | IPI00124700 | 5.4 | 3 | Transferrin receptor protein 1 | mt |
| 1 | IPI00315100 IPI00201699 | 51.8 | 6 | Transforming protein rhoa Aplysia ras-related homolog A2 | mt |
| 1 | IPI00122342 (IPI00212014) IPI00318619 | 25.8 | 20 | Transitional endoplasmic reticulum atpase   Valosin containing protein | mt |
| 1 | IPI00130227 | 9.1 | 6 | Translation initiation factor IF-2 mitochondrial precursor | mt |
| 1 | IPI00201932 (IPI00122346) | 25 | 8 | Translocon-associated protein delta subunit precursor | ER |
| 1 | IPI00115787 | 7.7 | 4 | Transmembrane 9 superfamily protein member 2 precursor | mt |
| 1 | IPI00336538 IPI00373155 (IPI00221697) | 11 | 10 | Transmembrane 9 superfamily protein member 4 homolog Similar to Transmembrane 9 superfamily protein member 4 | mt |
| 0.89 | IPI00189652 | 7 | 3 | Transmembrane protein tmdc I precursor | membrane |
| 0.97 | IPI00221523 IPI00360300 | 3.8 | 2 | Transportin 1 Similar to karyopherin beta 2 | unknown |
| 1 | IPI00199421 IPI00137296 | 14.8 | 2 | Trax Translin associated protein X | mt |
| 1 | IPI00126253 | 4.2 | 2 | Trh3 | mt |
| 1 | IPI00327694 | 40.8 | 24 | Tricarboxylate transport protein mitochondrial precursor | unknown |
| 1 | IPI00223092 | 58.8 | 54 | Trifunctional enzyme alpha subunit | mt |
| 1 | IPI00212622 | 42.5 | 32 | Trifunctional enzyme alpha subunit mitochondrial precursor | mt |
| 1 | IPI00330760 | 30.2 | 4 | Triosephosphate isomerase | mt |
| 1 | IPI00187402 | 28.6 | 12 | TRNA-nucleotidyltransferase 1 mitochondrial precursor | mt |
| 1 | IPI00110753 (IPI00189795) | 69.6 | 16 | Tubulin alpha-1 chain |  |
| 1 | IPI00117350 IPI00362927 | 53.1 | 5 | Tubulin alpha-4 chain Similar to Tubulin alpha-4 chain (Alpha-tubulin 4) | mt |
| 1 | IPI00197579 (PI00117352) | 69.1 | 9 | Tubulin beta-5 chain | mt |
| 1 | IPI00131309 (IPI00210181) | 21.7 | 4 | Tubulin gamma-1 chain | mt |
| 1 | IPI00126635 | 44.5 | 37 | Tumor-related protein | mt |
| 0.65 | IPI00113477 | 6.2 | 2 | Type IV collagen alpha 5 chain | unknown |
| 1 | IPI00230835  IPI00230707 | 38.9 | 7 | Tyrosine 3-monooxgenase\tryptophan 5-monooxgenase activation protein gamma polypeptide 3-monooxgenase\tryptophan 5-monooxygenase activation protein gamma polypeptide | mt |
| 1 | IPI00138131 | 38 | 15 | Tyrosine 3-monooxygenase | mt |
| 1 | IPI00230682 | 35.8 | 6 | Tyrosine 3-monooxygenase\tryptophan 5-monooxygenase activation protein beta polypeptide | mt |
| 1 | IPI00378177 IPI00314153 (IPI00366785) | 10.4 | 2 | Tyrosyl-tRNA synthetase Similar to tyrosyl-tRNA synthetase | mt |
| 1 | IPI00119138 | 41.9 | 39 | Ubiquinol-cytochrome C reductase complex core protein 2 mitochondrial precursor | mt |
| 1 | IPI00111885 | 40.8 | 37 | Ubiquinol-cytochrome C reductase complex core protein I mitochondrial precursor | mt |
| 1 | IPI00406482 IPI00170018 IPI00407382 IPI00224210 | 28.4 | 6 | Ubiquinol-cytochrome C reductase complex ubiquinone-binding protein QP-C Similar to RIKEN cDNA 1100001F06 gene Ubiquinol-cytochrome c reductase binding protein Low molecular mass ubiquinone-binding protein | mt |
| 1 | IPI00125592 | 41 | 7 | Ubiquinone biosynthesis protein COQ7 homolog | mt |
| 1 | IPI00113214 | 9 | 3 | Ubiquitin carboxyl-terminal hydrolase 5 | membrane |
| 1 | IPI00313962 (IPI00204375) | 24.2 | 6 | Ubiquitin carboxyl-terminal hydrolase isozyme L1 | mt |
| 1 | IPI00165854 IPI00367069 IPI00190559 | 28.3 | 5 | Ubiquitin-conjugating enzyme E2 N Similar to hypothetical protein FLJ36991 Ubiquitin-conjugating enzyme E2N (homologous to yeast UBC13) | mt |
| 1 | IPI00199424 IPI00407540 IPI00364537 (IPI00119227) IPI00359385 | 12.9 | 2 | Ubiquitin-like protein SUMO-1 conjugating enzyme Similar to ubiquitin conjugating enzyme E2I Similar to UBE2I protein  Similar to iroquois homeobox protein 6 | mt |
| 0.76 | IPI00112716 | 2.9 | 2 | Ubiquitin-protein ligase UBE3B | unknown |
| 1 | IPI00119478 | 15.6 | 2 | Ubiquitous tropomodulin | cytoplasm |
| 0.97 | IPI00165799 IPI00371952 | 4.2 | 2 | UBX domain-containing protein 2 Similar to RIKEN cDNA 1300013G12 | mt |
| 0.99 | IPI00330383 | 9.3 | 2 | UDP-galnac:polypeptide N-acetylgalactosaminyltransferase-T3 | mt |
| 1 | IPI00409907 | 41.5 | 62 | UDP-glucose ceramide glucosyltransferase-like 1 | mt |
| 1 | IPI00202664 | 18.3 | 3 | UDP-glucuronate decarboxylase | unknown |
| 1 | IPI00117611  IPI00227374 | 5.5 | 2 | UDP-N-acetyl-alpha-D-galactosamine:polypeptide N-acetylgalactosaminyltransferase 7 UDP-N-acetyl-alpha-D-GALACTOSAMIN E:polypeptide N-acetylgalactosaminyltransferase 7 homolog | ER |
| 0.6 | IPI00405720 | 8.2 | 2 | Unknown protein | unknown |
| 1 | IPI00137601 | 21.1 | 5 | UPF0082 protein | unknown |
| 1 | IPI00400078 IPI00202111 | 17.2 | 2 | Upregulated during skeletal muscle growth 5 DAPIT protein | mt |
| 1 | IPI00313841 | 22.2 | 4 | Vacuolar ATP synthase subunit d | mt |
| 1 | IPI00198291 | 45.4 | 5 | Vacuolar ATP synthase subunit F | unknown |
| 1 | IPI00387192 IPI00221838 | 7.4 | 3 | Vacuole membrane protein 1 homolog Similar to RIKEN cDNA 4930579A11 gene | mt |
| 1 | IPI00320462 | 17.7 | 3 | Valacyclovir hydrolase precursor | unknown |
| 1 | IPI00130353 | 15.4 | 14 | Valyl-tRNA synthetase 2 | mt |
| 1 | IPI00132276 (IPI00210971) | 38.8 | 5 | Vesicle-associated membrane protein 3 | mt |
| 1 | IPI00209290 (PI00125267) | 35.5 | 10 | Vesicle-associated membrane protein-associated protein A | mt |
| 1 | IPI00135655 IPI00308187 | 30.9 | 8 | Vesicle-associated membrane protein-associated protein B Unknown EST | mt |
| 0.98 | IPI00321634 IPI00210524 (IPI00308609) | 13.7 | 2 | Vesicular integral-membrane protein VIP36 precursor Similar to Vesicular integral-membrane protein VIP36 precursor | unknown |
| 1 | IPI00330862 | 10.6 | 2 | Villin 2 | mt |
| 1 | IPI00227299 | 39.5 | 18 | Vimentin | mt |
| 0.69 | IPI00210089 | 4 | 2 | Volatage-gated sodium channel | mt |
| 1 | IPI00210000 | 69 | 7 | Voltage-dependent anion-selective channel protein 1 | mt |
| 1 | IPI00122547 | 52.2 | 20 | Voltage-dependent anion-selective channel protein 2 | mt |
| 1 | IPI00122548 IPI00115160 | 57.2 | 18 | Voltage-dependent anion-selective channel protein 3 Ensembl_locations(Chr-bp):13-17849781 | unknown |
| 0.89 | IPI00130878 IPI00388311 (IPI00189595) | 2.7 | 2 | Voltage-dependent T-type calcium channel alpha-1H subunit Ensembl_locations(Chr-bp):10-14622136 | unknown |
| 0.99 | IPI00121851 | 14.8 | 2 | Von Hippel-Lindau binding protein 1 | mt |
| 0.78 | IPI00210224 | 9.3 | 2 | Von Willebrand factor | unknown |
| 1 | IPI00405552 IPI00170013 | 8.2 | 4 | Weakly Similar to acyl-coa dehydrogenase Hypothetical protein | mt |
| 1 | IPI00228113 | 43.2 | 59 | Weakly Similar to C1-tetrahydrofolate synthase | mt |
| 1 | IPI00350143 (IPI00403110) IPI00279051 | 4.9 | 2 | Weakly Similar to CG17660 protein  RIKEN cDNA A930025J12 | Golgi |
| 1 | IPI00221828 | 56.3 | 22 | Weakly Similar to DEPHOSPHO-coa kinase | mt |
| 1 | IPI00226656 | 25.1 | 7 | Weakly Similar to GENETHONIN 1 | mt |
| 0.7 | IPI00222063 | 8.1 | 2 | Weakly Similar to hypothetical 71.7 kda protein | unknown |
| 0.99 | IPI00108253 IPI00324585 | 3.8 | 2 | Weakly Similar to integrin alpha 5 Integrin alpha-1 precursor | mt |
| 0.74 | IPI00227736 | 4.1 | 2 | Weakly Similar to NCK-associated protein NAP5 | unknown |
| 1 | IPI00222037 | 18.3 | 22 | Weakly Similar to NPIP-like protein | mt |
| 1 | IPI00222679 | 20.2 | 5 | Weakly Similar to NPIP-like protein | mt |
| 1 | IPI00222125 | 45.8 | 6 | Weakly Similar to putative O-methyltransferase | mt |
| 0.65 | IPI00226216 | 5.6 | 2 | Weakly Similar to RHO guanine nucleotide exchange factor 5 | unknown |
| 0.87 | IPI00403493 | 53.9 | 2 | Weakly Similar to Similar to protein phosphatase 1 | unknown |
| 0.91 | IPI00223432 | 7.5 | 2 | Weakly Similar to SNAP190 | mt |
| 1 | IPI00111460 IPI00189798 | 9.5 | 3 | Williams-Beuren syndrome chromosome region 16 protein homolog Similar to Williams-Beuren syndrome chromosome region 16 homolog | mt |
| 1 | IPI00170213 | 53.7 | 13 | Williams-Beuren syndrome critical region protein 21 | mt |
| 1 | IPI00207864 | 4.5 | 3 | Xrcc5 | lysosome |
| 0.65 | IPI00283229 | 17 | 2 | Zinc finger OVO2 isoform B | unknown |
| 1 | IPI00322820 IPI00222412 | 5.7 | 2 | Zmpste24 Inferred: CAAX prenyl protease 1 homolog | membrane |
| 0.93 | IPI00192475 | 11.4 | 2 | Zona pellucida B glycoprotein | unknown |
| 0.78 | IPI00352528 | 5.6 | 2 | Zonadhesin precursor | unknown |
